# Geology defines microbiome structure and composition in nunataks and valleys of the Sør Rondane Mountains, East Antarctica

**DOI:** 10.3389/fmicb.2024.1316633

**Published:** 2024-02-06

**Authors:** Valentina Savaglia, Sam Lambrechts, Bjorn Tytgat, Quinten Vanhellemont, Josef Elster, Anne Willems, Annick Wilmotte, Elie Verleyen, Wim Vyverman

**Affiliations:** ^1^InBioS Research Unit, Department of Life Sciences, University of Liège, Liège, Belgium; ^2^Laboratory of Protistology and Aquatic Ecology, Department of Biology, Ghent University, Ghent, Belgium; ^3^Laboratory of Microbiology, Department of Biochemistry and Microbiology, Ghent University, Ghent, Belgium; ^4^Institute for Natural Sciences, Brussels, Belgium; ^5^Faculty of Science, Centre for Polar Ecology, University of South Bohemia České Budějovice and Institute of Botany, Třeboň, Czechia

**Keywords:** microbial ecology, Antarctica, bedrock, rRNA, bacteria, eukaryotes, metabarcoding

## Abstract

Understanding the relation between terrestrial microorganisms and edaphic factors in the Antarctic can provide insights into their potential response to environmental changes. Here we examined the composition of bacterial and micro-eukaryotic communities using amplicon sequencing of rRNA genes in 105 soil samples from the Sør Rondane Mountains (East Antarctica), differing in bedrock or substrate type and associated physicochemical conditions. Although the two most widespread taxa (Acidobacteriota and Chlorophyta) were relatively abundant in each sample, multivariate analysis and co-occurrence networks revealed pronounced differences in community structure depending on substrate type. In moraine substrates, Actinomycetota and Cercozoa were the most abundant bacterial and eukaryotic phyla, whereas on gneiss, granite and marble substrates, Cyanobacteriota and Metazoa were the dominant bacterial and eukaryotic taxa. However, at lower taxonomic level, a distinct differentiation was observed within the Cyanobacteriota phylum depending on substrate type, with granite being dominated by the Nostocaceae family and marble by the Chroococcidiopsaceae family. Surprisingly, metazoans were relatively abundant according to the 18S rRNA dataset, even in samples from the most arid sites, such as moraines in Austkampane and Widerøefjellet (“Dry Valley”). Overall, our study shows that different substrate types support distinct microbial communities, and that mineral soil diversity is a major determinant of terrestrial microbial diversity in inland Antarctic nunataks and valleys.

## Introduction

Ice free-regions cover only a minute fraction of the Antarctic continent (0.4% of its surface area; Brooks et al., 2019), and occur as geographically isolated patches along the coastline, inland nunataks and mountain chains. They represent some of the most extreme environments on Earth, with a general lack of moisture due to very low amounts of precipitation and unpredictable and sporadic snow and ice melt, strong katabatic winds that scour surfaces and result in the build-up of snow banks, high levels of ultraviolet radiation, and extremely low and daily/seasonally fluctuating temperatures (Bockheim and Hall, 2002; Cowan and Tow, 2004). It is now well known that despite these extreme conditions, the arid and hyper-arid Antarctic soils host a much higher microbial diversity than previously thought (Cowan et al., 2014; Lukashanets et al., 2021; Ortiz et al., 2021). In addition, there is cumulative evidence that similarly to macroscopic organisms, a relatively high number of microbial taxa is endemic to the Antarctic as a result of evolution in isolation on multi-million-year timescales (Convey et al., 2020; Pinseel et al., 2020; Ortiz et al., 2021; Verleyen et al., 2021; Tytgat et al., 2023). This implies that some regions must have been ice free during the multiple glacial (re-)advances that took place since the formation of the Antarctic Ice Sheets c. 35 Mya, and could act as refugia for terrestrial and lacustrine biota (Convey et al., 2008; Pugh and Convey, 2008; Stevens and D’Haese, 2014; Convey et al., 2020; Pinseel et al., 2021).

In addition to their high number of endemic taxa, Antarctic terrestrial food webs are characterized by the absence of any vertebrates, and nearly all invertebrate and plant groups. These soil ecosystems are therefore mainly driven by microorganisms, and have truncated and simple food webs. Similarly to other deserts, edaphic microbial communities represent the dominant players in biogeochemical cycling (Pointing and Belnap, 2012; Makhalanyane et al., 2015; Ramond and Cowan, 2022). Despite this, the relation between environmental factors and microbial community structure in Antarctic ice-free regions is still poorly studied, with the exception of those in the arid deserts of the McMurdo Dry Valleys and the Transantarctic region of Victoria Land (Thompson et al., 2020; Coleine et al., 2021; Severgnini et al., 2021; Solon et al., 2021; Dragone et al., 2022). This lack of knowledge is particularly important for microbial eukaryotes (Obbels et al., 2016; Franco et al., 2021; Cucini et al., 2022), which is in fact also the case for the non-fungal microbial eukaryotes in soils worldwide (Oliverio et al., 2020; Ramond and Cowan, 2022). The few existing studies for Antarctic microbial soil communities revealed that soil moisture, pH, salinity, nitrogen concentration, soil physical stability and to a lesser degree temperature and organic matter content of the soils can explain a significant portion of the spatial variability (Niederberger et al., 2008; Goordial et al., 2016; Tytgat et al., 2016; Alekseev et al., 2020; Severgnini et al., 2021; Dragone et al., 2022). In top-soils of hyper-arid areas in the Antarctic, Chlorophyta, Ciliophora and Cercozoa are the most abundant eukaryotic phyla recorded, while Actinomycetota, Acidobacteriota, Bacillota and Chloroflexota are often the dominant bacterial phyla, similarly to edaphic niches of hot deserts (Ramond and Cowan, 2022). Some phylotypes of these bacterial phyla were recently found to be genetically equipped for the consumption of molecular hydrogen (H_2_), CO_2_ and CO from the atmosphere as energy and carbon sources (Ji et al., 2017; Ortiz et al., 2021; Greening et al., 2022; Ray et al., 2022). Actinomycetota are also abundant in environments with a higher organic carbon and moisture content, such as biological soil crusts (BSCs) or lithic niche refugia (i.e., hypolithic, endolithic habitats or wind protected rock cavities). However, in these sheltered habitats, other bacterial taxa such as Cyanobacteriota, Pseudomonadota (formerly Proteobacteria) and Bacteroidota are also abundant, similarly to lithic-associated niches of hot deserts (Ramond and Cowan, 2022). Among the eukaryotes, lichen-symbiotic and free-living Fungi, and Chlorophyta may be abundant, as well as the metazoan Rotifera, Tardigrada, Nematoda and Arthropoda. Cryptoendolithic communities of both hot and cold deserts have been extensively studied, with results showing the importance of the lithologic diversity as one of the main drivers of their structure (Archer et al., 2016; Meslier et al., 2018). Nonetheless, little attention has been given to the influence of the substrate type to the open soil communities in both environments (but see Tytgat et al., 2016; Lulákova et al., 2023 for Antarctic soils).

Along with the identification of the structuring role of abiotic factors on microbial communities, co-occurrence networks between bacterial and (micro)eukaryotic phylotypes can reveal potential biotic interactions which may exert an unexpected, yet critical control on the complexity of these abiotically driven polar ecosystems (Lee et al., 2019). Co-occurrence networks can also be used to identify keystone taxa, which are those phylotypes that form a link between two hubs of taxa and/or that highly co-occur with other taxa, and are expected to play a pivotal role in the ecological cluster (or module) to which they belong. Identifying the key taxa performing crucial ecosystem functions such as nitrogen and carbon fixation is crucial to understand the threats these communities may face in response to anthropogenic and climate disturbances.

**Figure 1:**
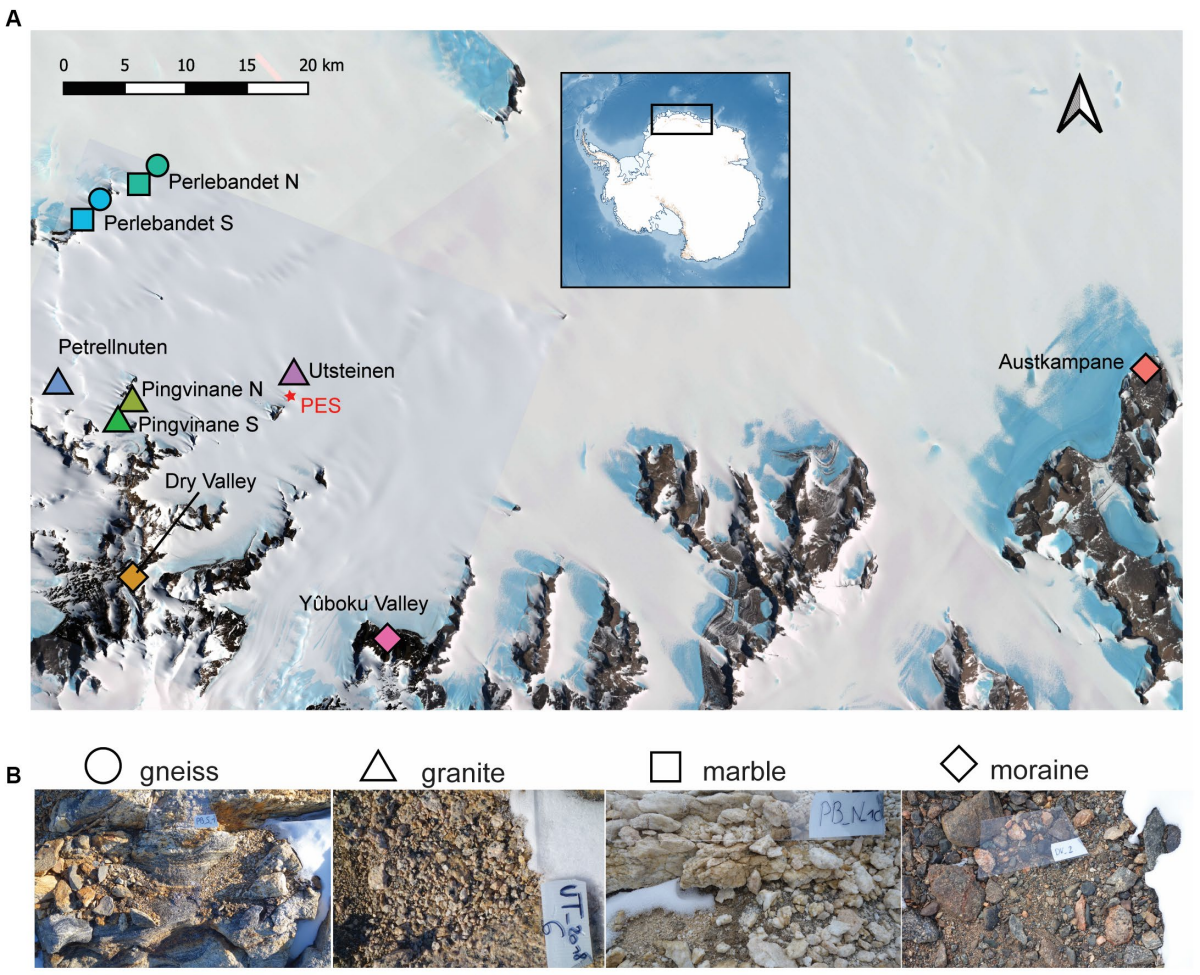
**(A)** Satellite image of Sør Rondane Mountains and sampled sites. **(B)** *In situ* photographs of the four types of soil ecosystems investigated. More information about the samples can be found and in Supplementary Figure 1. Contains modified Copernicus Sentinel-2 data [2020] and Landsat Image Mosaic of Antarctica (LIMA). Colors differ by sampled site. AU, Austkampane; DV, Dry Valley; PB_N, Perlebandet North; PB_S, Perlebandet South; PA4, Pingvinane 4th nunatak; PA6, Pingvinane 6th nunatak; PT, Petrellnuten; UT, Utsteinen; YO, Yûboku-dani Valley.

Here we analyzed environmental conditions and the edaphic microbial community structure in 105 soil samples collected from 9 different sites in the Sør Rondane Mountains, East Antarctica (Figure 1; Supplementary Figure S1). This region includes several isolated ice-free nunataks and associated moraines, encompassing a variety of substrate types and local environmental conditions (Tytgat et al., 2016). We hypothesized that (i) different substrate types support particular bacterial and micro-eukaryotic communities [i.e., a higher abundance of filamentous cyanobacteria within granitic compared to gneiss substrates, as was already observed in Tytgat et al. (2016) and Pushkareva et al. (2018)], and different keystone phylotypes possessing alternative primary production strategies (i.e., photosynthetic cyanobacteria in the most wet soils versus chemosynthetic Actinobacteriota in the driest soils), and (ii) metazoans are restricted to environments with relatively high primary production by photosynthetic Cyanobacteriota and micro-eukaryotes.

## Materials and methods

### Sampling and site descriptions

The Sør Rondane Mountains (SRM; 22–28° E, 71°30′–72°40′ S) encompass a large number of nunataks covering an area of ca 2,000 km^2^ (Suganuma et al., 2014) within Dronning Maud Land, East Antarctica. The SRM are geologically situated in the East African-Antarctic Orogen (EAAO; Jacobs and Thomas, 2004) that was formed by the collision of the African plate assembly and the Antarctic Coats Land Block around 650–540 Ma, causing the accretion of multiple arc terranes (Ruppel et al., 2015). In between these two plates, the Main Shear Zone separates the northern SRM with ocean affinity (*viz.* the presence of marble veins) and characterized by metamorphic rocks (i.e., gneiss), from the southern metamorphic rocks that exhibit island arc or continental margin arc features (igneous intrusions, e.g., granite outcrops). In this study, nine sites originating from these two plates were sampled to cover the main geological features in the western SRM (Figure 1). The granitic Utsteinen ridge (“UT”) north of the Utsteinen nunatak was sampled, close to the Belgian Station Princess Elisabeth Antarctica (“PEA”; 71°57’ S, 23°21′ E; 1,372 m a.s.l.). Perlebandet to the northwest of PEA is a mountain range composed of three nunataks mainly characterized by gneiss soils interlayered with some marble veins in the two northernmost nunataks (designated here as “Perlebandet N” or “PB_N,” and “Perlebandet S” or “PB_S”). To the southwest of PEA, near the main mountain range, Petrellnuten is located, which is a granitic nunatak close to the Pingvinane mountain subrange. Pingvinane is characterized by five granitic nunataks, two of which were sampled (named “Pingvinane 4^th^” or “PA4,” and “Pingvinane 6^th^” or “PA6”). Furthermore, three moraine sites were sampled: Widerøefjellet (here referred to as “Dry Valley” or “DV”) to the south-west, Yûboku-dani Valley (“YO”) to the south-east and Austkampane (“AU”) to the east of PEA. Gneiss and granitic bedrock types are chemically similar but physically different. Because of the high compression gneiss has been subjected to during metamorphism, it is composed of layers of minerals (silicates, aluminum, potassium, etc.) which make the rock very hard and of low porosity. By contrast, granitic rocks are characterized by randomly dispersed minerals within the rock matrix, causing the rock to be more porous and less hard. Marble, on the other hand, is a metamorphosed limestone, mainly composed of calcium carbonates and magnesium, characterized by an even higher porosity and lower hardness than both the granite and gneiss bedrock types. The moraines consist of a mixture of fragments of different types of rocks, thus with different physical and chemical characteristics. The sampled moraine soils were characterized by amphibolite (Austkampane) or greenschist-facies origins (Yûboku-dani Valley and Dry Valley) with gneiss, quartz and/or meta-tonalite inclusions (see Supplementary Table S1 for details). More details about the sampling locations are given in Supplementary material and Supplementary Figure S1.

The SRM have an east–west orientation, constituting a barrier to the northern ice plateau. This position is of particular advantage to the northern escarpment zone where most of the nunataks are located. Here, the strong north-facing katabatic winds are naturally deviated, resulting in rather sheltered areas, including Perlebandet and Utsteinen where PEA is located. Winters are relatively mild, with temperatures oscillating between 25 to −20°C during winter and summer temperatures between −15 and +5°C (Pattyn et al., 2009; Gorodetskaya et al., 2013). In opposition, conditions near the southern main mountain range are more extreme, with Austkampane to the east being the windiest site among all the sampled locations, followed by the Dry Valley area. Because of the continuous winds, these two sites are particularly harsh and microbial communities are often not visible.

During the Belgian Antarctic Research Expeditions (BELARE) in 2017–2018 and 2018–2019, 105 samples from 9 sites (Figure 1) were taken along elevational gradients in nunataks and valleys of different substrate types, having different geochemical properties, moisture content, and visible biomass (Supplementary Table S1). In each sampled site, we retrieved the diversity of edaphic communities observed *in situ*, by collecting the most heterogeneous kind of communities, ranging from exposed barren bedrock to protected substrates close to boulders (when present) where diverse development stages of biological soil crusts were visible and consisted of macroscopic biota such as lichens, mosses, microalgae and/or cyanobacteria. This sampling design was chosen to have a representative overview of the biota inhabiting the surface substrate (top-soil, 0–2 cm depth) consisting of gravel soil in the sampled nunataks and valleys. Nonetheless, difficulties in accessing specific nunataks or the low extent of biomass present contributed to an unbalanced sampling design, resulting in more samples from specific locations, compared to fewer samples from other sites. All samples were collected aseptically using a sterilized spoon (70% ethanol), immediately stored in sterile Whirl-Pak® sample bags and transported in a cool box with ice-packs (−20°C) until arrival at the station. All the samples were stored at −80°C at the station and brought to Belgium at −20°C, where they were stored at −20°C until further processing at the Laboratory of Protistology and Aquatic Ecology (Ghent University). When sufficient material was collected for the analysis of soil characteristics, pH, electric conductivity, moisture content (dry weight) and the concentrations of nutrients (N-NO_3_^−^, N-NH_4_^+^, P-PO_4_^3−^, total nitrogen, total phosphorus) and organic matter (soil total organic carbon, TOC) were analyzed. Insufficient material for DNA and soil characterizations affected the overall number of samples that could be used for downstream analysis and therefore further contributed to the resulting unbalanced design.

### DNA extraction and sequencing

High-quality bacterial 16S and eukaryotic 18S rRNA gene libraries were successfully constructed for 96 and 97 soil samples, respectively. Each sample was first thoroughly homogenized, and between 0.2 to 1 g was used for DNA extraction with the Qiagen DNeasy Powersoil kit (Valencia, CA) depending on the type of sample (i.e., mineral soils needed a higher amount of sample compared to soils with macrobiota). For bacteria, PCRs targeting the V1-V3 hypervariable regions of the 16S rRNA gene were performed using the general forward pA (5’-AGAGTTTGATCCTGGCTCAG-3′, positions 8–27) (Edwards et al., 1989) and reverse BKL1 (5’-GTATTACCGCGGCTGCTGGCA-3′, positions 536–516) (Cleenwerck et al., 2007) primers. For eukaryotes, the V4 region of the 18S rRNA gene was amplified using the general forward TAReuk454FWD1 (5′-CCAGCASCYGCGGTAATTCC-3′) and reverse TAReukREV3 (5′-ACTTTCGTTCTTGATYRA-3′) (Stoeck et al., 2010) primers. The PCR mixture consisted of 2.5 μL buffer (FastStart High Fidelity PCR System, Roche), 5 μL (2 mM) dNTP (Thermo Fisher), 2.5 μL (10 μM) of each primer, 0.25 μL (1 u μl^−1^) Taq polymerase (FastStart High Fidelity PCR System, Roche), 1 μL template DNA and 14.75 μL sterile milliQ water. An initial denaturation step of 5 min at 94°C was followed by 25 or 35 cycles for bacterial or eukaryotic primers, respectively, which consisted of 1 min denaturation at 94°C, 1 min annealing at 65 to 60°C or 57 to 52°C for bacterial or eukaryotic primers, respectively, with a decrease of 0.5°C per cycle, and an elongation step at 72°C for 3 min. After a final elongation of 20 min at 72°C, PCR products were visualized on TBE 1% agarose gel. In case of PCR failure, 2 μL template DNA was used instead of 1 μL, and/or 40 cycles were used instead of 35 cycles or 30 cycles instead of 25 for bacterial or eukaryotic primers, respectively. If amplification was successful, PCR duplicates for each sample were pooled to minimize potential biases. The pooled PCR products were subsequently purified with Agencourt AMPure XP beads (Beckman Coulter Inc.) according to the manufacturer’s instructions. Twelve to sixteen-cycle (depending on the amount of DNA) index PCRs using Nextera XT (Illumina Inc.) barcodes were performed as described in the Illumina 16S rRNA metagenomics library prep guide using 25 μL of PCR grade water, 5 μL of 5x buffer, 5 μL of dNTP (10 mMol), 0.5 μL Taq Polymerase (FastStart High Fidelity PCR System, Roche), 3.5 μL of both index 1 and index 2, and 7.5 μL of PCR template. The library prep was again purified using AMPure Beads XP (Beckman Coulter Inc.) as above. The DNA concentration of the cleaned libraries was measured using a Qubit fluorometer (ThermoFisher) with the Qubit dsDNA BR assay kit (ThermoFisher), and the samples were equimolarly pooled based on their molecular weight and DNA concentrations in a total volume of 15 μL and a final concentration of at least 5 nM. Amplicon libraries were sequenced on an Illumina Miseq platform (2 × 300 bp paired-end, v3 chemistry) at Genewiz (Azenta Life Sciences, Leipzig, Germany). Each run with either eukaryote or bacteria samples contained two replicates of a positive control sample (mock community), respectively containing 16 eukaryotic (Supplementary Table S2) and 20 bacterial species (20 Strain Even Mix Genomic Material, ATCC, Cat. No. MSA-1002, Manassas, VA, United States), two negative controls (each composed by a mixture of two PCR blanks) to check overall run quality and benchmark processing variables, as well as 2–3 PCR replicate samples. Sequences were deposited to NCBI Sequence Read Archive under BioProject accession number PRJNA1044841.

### Data processing

Raw reads were processed using the DADA2 package version 3.10 (Callahan et al., 2016) within R version 4.0 (R Core Team, 2021). For both 16S and 18S rRNA genes, primers were removed from the raw reads by trimming the first 20 and 21 (18 for 18S rRNA) nucleotides from the forward and reverse reads, respectively, using the *trimLeft* command. Based on manual inspection using FastQC (Andrews, 2010), forward and reverse reads wSUB13877824ere then truncated at base pair 297 and 257 (267 for eukaryotic reads), respectively, using the *truncLen* command and filtered with a maximum number of “expected errors” (*maxEE*) threshold of 2 for both forward and reverse reads. Reads not matching these criteria were discarded. After sequence dereplication, sequence variants for the forward and reverse reads were inferred based on an error matrix constructed from the first 1e8 bp of the sequences. Paired-end reads were merged with no mismatch allowed and a required minimum overlap of 20 bp. Sequence tables for each sequencing run were constructed using *makeSequenceTable*, and subsequently merged with *mergeSequenceTables* commands. Finally, chimeric sequences were removed using *removeBimeraDenovo*. Taxonomic classification of the resulting sets of ASVs was done using *classify.seqs* in Mothur with the SILVA v138.1 (Pruesse et al., 2007) and PR2 version 4.14.0 (Guillou et al., 2012) databases for the 16S and 18S ASV sets, respectively.

### Environmental data collection

Geographical coordinates of each sampled location were taken with a Garmin 62nd/64st/Montana® 680 GPS. Elevation data were retrieved from the geographical coordinates as described in Vanhellemont et al. (2021) and are shown in Figure 1 and in Supplementary Table S1. Soil was passed through a sterile 3 mm sieve for homogenization and to remove large particles before analysis. Analysis of geochemical and geophysical soil properties such as pH (H_2_O), soil total organic carbon (TOC,%), N-NO_3_^−^ (mg kg^−1^), N-NH_4_^+^ (mg kg^−1^), total nitrogen (TN,%), P-PO_4_^3−^ (mg kg^−1^), total phosphorus (TP,%) and soil dry weight (%) and electric conductivity (EC, μS cm^−1^) were conducted following established methods at the Institute of Botany, AS CR in Třeboň (Czech Republic). Analyses were performed according to Czech and European Union standards (ISO 10390, ISO 10523, ČSN EN 27888, ISO 11465, ČSN EN ISO 11732, ČSN EN ISO 13395, and ČSN EN ISO 15681–1). Conductivity (μS cm^−1^) and pH were evaluated in demineralized and distilled water, respectively. The samples were further dried at 105°C to constant weight and then combusted at 450°C. Soil organic matter content (SOM) was calculated as the difference between the two weights. Total phosphorus (TP) was estimated using spectrometric determination of phosphorus soluble in sodium hydrogen carbonate solution. Mineral nitrogen (Nmineral) was calculated as the sum of N–NH_4_^+^, N–NO_3_^−^ and N-NO_2_^−^ concentrations, which were measured using a QuikChem®8500FIA automated ion analyser (Lachat Instruments, Loveland, United States). In addition to elevation, these parameters were included as “environmental variables” for each sample (Supplementary Figure S2; Supplementary Table S1). The different bedrock types that characterized each soil sample were defined as in Shiraishi et al. (1997) and confirmed with pictures taken in the field. Bedrock type variables were binary coded (1: sampled type of bedrock; 0: other types of bedrock).

### Statistical analysis

All statistical calculations were conducted in R 4.0 (R Core Team, 2021).

Singletons (defined as an ASV with a sequence that is present exactly once across the whole dataset), mitochondrial and chloroplast-related sequences, and potential contaminants (following Fullerton et al., 2021) were removed. After occurrence inspection, mock communities, blanks and replicate samples were removed from the ASV tables and only one sample with the highest read number was kept among replicates of the same sample (Supplementary Figures S3, S4).

Diagnostic plots, skewness and kurtosis (0.14 version of the *moments* package; Komsta and Novomestky, 2015) were used to identify the necessary transformations in order to improve the unimodal distribution of the environmental variables. To this purpose, the environmental variables were centered and scaled after square root (P-PO_4_^3−^) or log (elevation, TOC, N-NO_3_^−^, N-NH_4_^+^, TN, TP, dry weight, conductivity) transformations. Pearson’s coefficients with 999 permutations and the variance inflation factor (VIF) with a stepwise calculation were used to check for collinearity among the transformed environmental parameters. Pearson’s coefficients were calculated using the *cor* function in the 0.92 version of the corrplot package (Wei and Simko, 2021) (Supplementary Figure S5). VIF factors were calculated using the *vistep* function of the 1.1.18 version of the usdm package (R Core Team, 2021). No strong correlations between any of the environmental variables were observed with both methods (Pearson’s |r| < 0.8 and VIF < 10), hence all were kept during further analysis (Supplementary Figure S5). Kruskal-Wallis rank sum tests were performed with the *kruskal.test* function of the 4.0.5 version of the stats package to detect significant differences in the values of geochemical variables in relation to the substratetype (Supplementary Table S3). Multiple pairwise-comparisons between groups were performed with the *pairwise.wilcox.test* function of the stats package to calculate pairwise comparisons between the bedrock types with Bonferroni corrections for multiple testing.

For beta-diversity, cumulative-sum scaling (CSS) was performed using the *cumNorm* function in the metagenomeSeq package (v1.32; Paulson et al., 2013). PCoA ordination and cluster analyses were conducted in phyloseq (version 1.34; McMurdie and Holmes, 2013) and were used to assess differences in community structure between the samples as well as to identify the potential explanatory environmental variables for the observed community patterns between the samples.

Pearson’s correlations between the environmental variables, elevation, the sampled sites, the substrate types, and, separately, the main bacterial and eukaryotic phyla were assessed in order to investigate a supplemental occurrence between all the measured variables.

Sampling plot coordinates were recorded as latitude and longitude. To test the influence of geographic distance at different spatial scales, and the effect of unmeasured environmental factors potentially varying between different regions on the bacterial and eukaryotic community structure, we used the spatial variables created by principal coordinates of neighborhood matrix (PCNMs) of the geographic coordinates of the samples (Borcard and Legendre, 2002), following Sakaeva et al. (2016). Briefly, using PCNMs allows to analyze the impact of spatial parameters on the communities at different spatial scales, ranging from large (PCNM1) to fine spatial scale (in our case, PCNM5). Therefore, geospatial coordinates of the samples were first used to calculate a geographic distance matrix using the 1.5.14 version of the geosphere package (Hijmans, 2021). Then, PCNM eigenvectors were calculated from the geographic distance matrix using the *pcnm* function implemented in the version 2.5.7 of the vegan package in R. We determined which PCNM were significantly correlated with the environmental variables using a cutoff of *p* < 0.05. Then, from the pool of significantly correlated PCNMs, we used a stepwise model selection to select the most parsimonious linear model (Venables and Ripley, 2002) linking the environmental and bedrock type variables to PCNM eigenvectors, as shown in Sokol et al. (2013).

To explain variation in community structure associated with explanatory variables, a distance-based redundancy analysis (dbRDA; McArdle and Anderson, 2001) was used to model variation in community structures as a function of either environmental (elevation, pH, TOC, N-NO_3_^−^, N-NH_4_^+^, TN, P-PO_4_^3−^, TP, dry weight, conductivity), spatial (PCNM) variables or bedrock type (as dummy variables for marble, granite, gneiss, moraine) after Euclidean transformation. The spatial variables and bedrock type were included to cover potentially important unmeasured environmental factors. Forward stepwise model selection (Blanchet et al., 2008) based on adjusted R^2^ values (Beisner et al., 2006; Peres-Neto et al., 2006; Nabout et al., 2009) was used to select the variables that best explained the variation in community structures.

To identify modules of strongly associated soil ASVs, a correlation network, i.e., co-occurrence network, was established, similarly to Delgado-Baquerizo et al. (2018a, 2018b) and Fullerton et al. (2021). These analyses were performed on 85 samples for which amplicon and environmental data were available for both bacteria and eukaryotes. To reduce the complexity of network visualization, rare ASVs were filtered out by defining thresholds based on a relative abundance > 0.1%, a global abundance of more than 5 reads, and presence in more than 3 samples (performed independently for the bacterial and eukaryotic datasets). These bacterial and eukaryotic ASVs were then merged into a single relative abundance table. We then calculated all pairwise Spearman’s rank correlations between all soil ASVs with a coefficient (ρ) > 0.7. We focused exclusively on positive correlations as they provide information on microbial taxa that may respond similarly to environmental conditions. A Spearman rank correlation was chosen over microbiome-specific correlation metrics (Friedman and Alm, 2012; Kurtz et al., 2015; Schwager et al., 2017) because, with *n* = 85, a Spearman coefficient equal to or above 0.355 is already sufficient (Zar, 1972) to retain the relationships that have a *p* < 0.001 and Spearman rank correlations have been used extensively in similar studies (Delgado-Baquerizo et al., 2018a, 2018b; Fullerton et al., 2021). Potential false-discovery rates were controlled through this strict alpha level, while maintaining good power with the high Spearman rank coefficient. Finally, the network was visualized with the interactive platform gephi (Bastian et al., 2009) such that ASVs served as “nodes” or vertices, and absolute correlation values as the “edges” between ASVs. A modularity analysis using different clustering algorithms in the R package igraph (1.2.6 version, Csardi and Nepusz, 2006) was performed (random walks, label propagation and Louvain clustering algorithms). A module is a cluster of highly interconnected nodes. While the total number of modules changed, the main modules identified by the tested algorithms converged and the 11 modules identified by the Louvain clustering algorithms (modularity of 0.79) were retained for downstream analysis. Identified modules represented ecological modules of ASVs showing a cohesive distribution across the sampled bedrock types. To investigate emergent patterns in the responses of these communities to soil-driven geochemical variables across the bedrock types, we coupled the co-occurrence network of ASVs to random forest (RF) variable ranking and correlations with Spearman and Pearson statistics. We then computed the relative abundance of each module by averaging the standardized relative abundances (z-score) of the ASVs that belong to each module, and we plotted them against the substrate type. By standardizing our data, we ruled out any effect of merging data from bacteria and eukaryotes. Kruskal-Wallis rank sum tests were performed to detect significant differences in the values of relative abundances per module in relation to the substrate type. Furthermore, the relationship between each module’s ASVs cumulative relative abundance and environmental predictors was investigated using an RFs regression analysis using the 1.1. version of the VSURF package (Genuer et al., 2015). The validity of the identified environmental predictors was further tested by correlating the cumulative abundance of each module, using both Pearson moment correlations and Spearman rank correlations, against all the environmental factors. Holm correction for multiple hypothesis testing was applied to all *p* values (Holm, 1979). Afterwards, scatterplots were used to manually inspect and confirm the statistically identified correlations in order to identify possible nonlinear relationships. With a consistent overlap of the identified environmental predictors by each different approach (RFs analysis, scatterplot inspection, Pearson moment and Spearman rank correlations), we consider that modules cohesively responding to variations in specific geochemical parameters might correspond to taxa that share habitat preferences that could be correlated to a specific bedrock type. The most biologically informative environmental variables that were associated with the distribution of each module were selected for plotting. Finally, the vertex degree and betweenness centrality of each node from the networks were measured for potential keystone taxa detection. Vertex degree represents the number of direct connections of a node with other ASVs in the whole community. Betweenness centrality reveals the role of a node as a bridge between components of a network. We considered ASVs with highest betweenness centrality and vertex degree in the network as potential keystone taxa (Martín González et al., 2010; Vick-Majors et al., 2014; Banerjee et al., 2016).

## Results

### Community structure and diversity in relation to substrate type

In total, Illumina sequencing resulted in 3,236,875 and 9,670,564 bacterial and eukaryotic reads which, after filtering and down-sampling, resulted in 3,852 to 111,385 reads for bacteria and 10,886 to 613,991 for eukaryotes. After bioinformatic data processing and removal of sample replicates, the remaining ASVs represented 78 and 75% of the original bacterial and eukaryotic reads, respectively. A total of 27,398 Bacterial ASVs were found across 96 samples and these were mainly classified as Actinomycetota (35%), Bacteroidota (15%) and Acidobacteriota (11%). Cyanobacteriota, Pseudomonadota, Abditibacteriota (formerly known as candidate division FBP) and Chloroflexota accounted for 8, 7, 5 and 4% of the total reads, respectively, while the remaining phyla consisted of Armatimonadota, Candidatus Patescibacteria, Planctomycetota and Deinococcota representing between 2 and 1%. Bdellovibrionota, Candidatus Dependentiae, Desulfobacterota, Elusimicrobiota, Bacillota, Gemmatimonadota, Myxococcota, Nitrospirota, Candidatus Sumerlaeota, Verrucomicrobiota and WPS-2 represented less than 1%.

**Figure 2:**
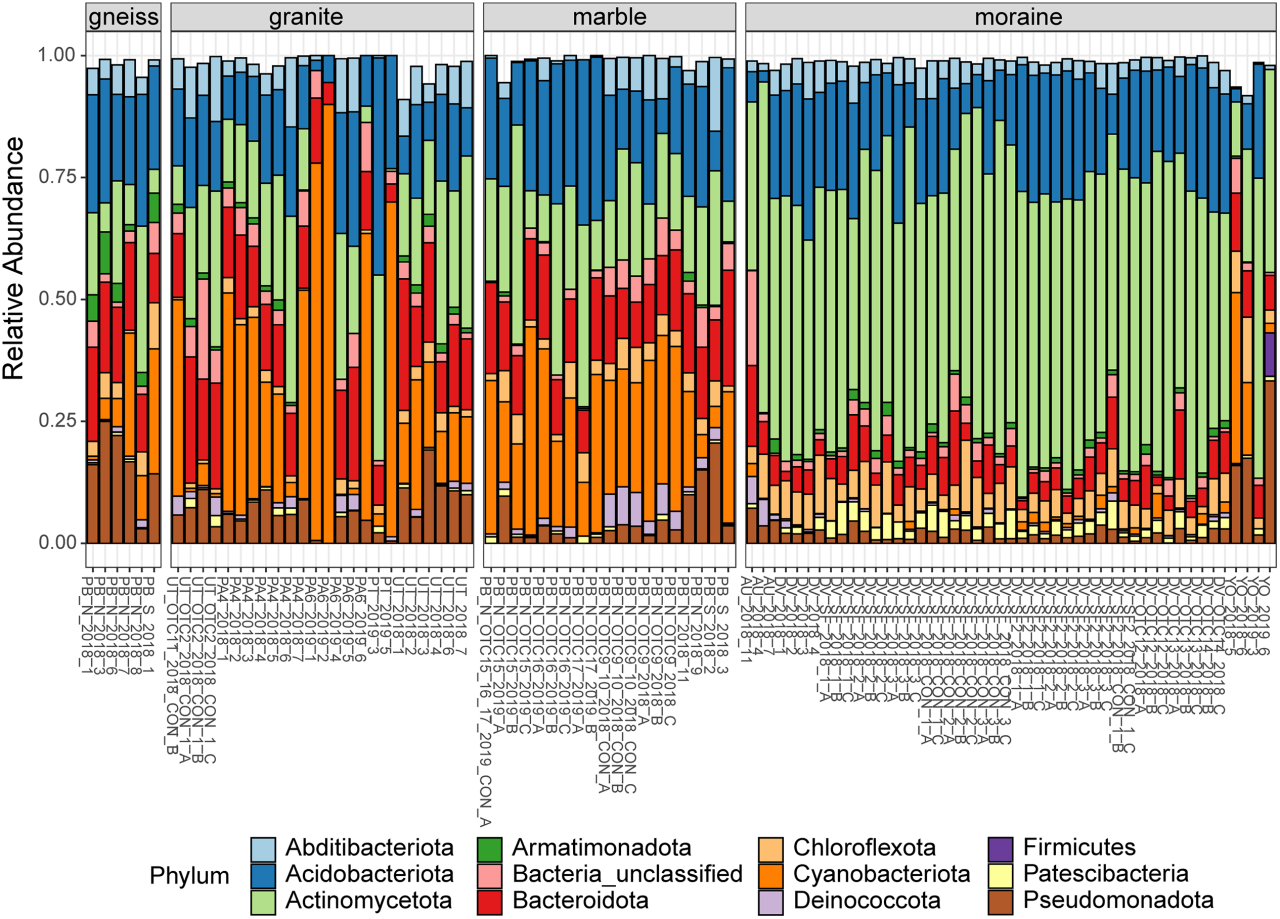
Relative abundances of the most abundant phyla (> 1% of total abundances) of bacteria per sample, grouped per substrate type. Colors are according to the Phylum (see legend). Sample names indicate the site of origin (see Supplementary Table S1 for details).

**Figure 3:**
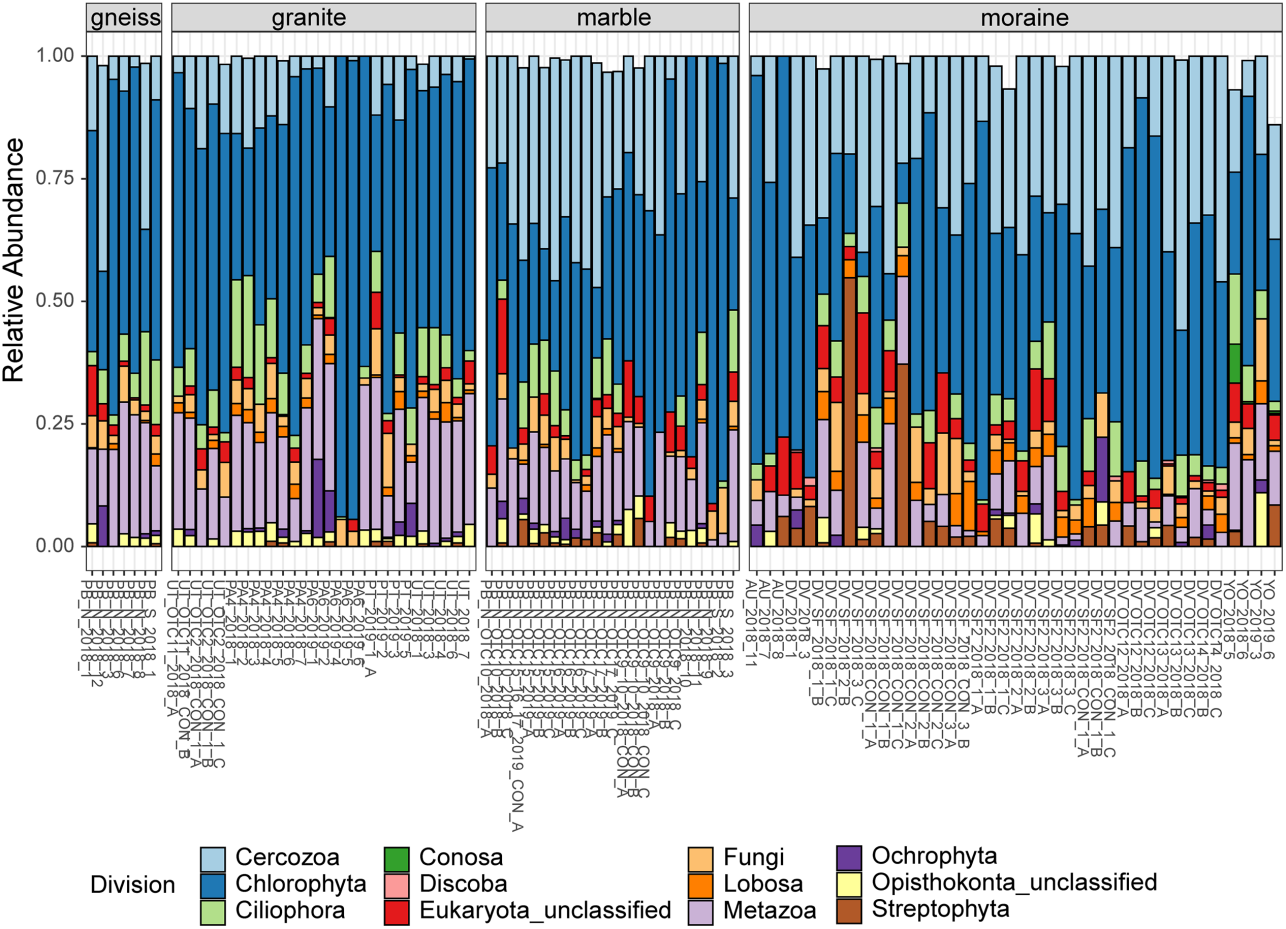
Relative abundances of the most abundant phyla (> 1% of total abundances) of eukaryotes per sample, grouped per substrate type. Colors are according to the Division (see legend). Sample names indicate the site of origin (see Supplementary Table S1 for details).

A total of 1,696 eukaryotic ASVs were detected across 97 samples. The ASVs mainly belonged to the division Chlorophyta (28%), Cercozoa (27%) and Metazoa (11%), followed by Ciliophora (7%), Streptophyta (4%) and Ochrophyta (2%). Less abundant phyla were represented by Opisthokonta and Lobosa accounting for less than 2%, and Apicomplexa, Archaeplastida, Centroheliozoa, Conosa, Cryptophyta, Dinoflagellata, Discoba, Opalozoa, and Pseudofungi represented less than 1% of the total reads. Interestingly, 8% of the bacterial (Figure 2) and eukaryotic reads (Figure 3) remained unclassified at the phylum or division level using the Silva and PR2 identification pipelines and thus might represent a significant fraction of novel diversity.

The relative abundance of several bacterial and eukaryotic taxa differed between the different substrate types (Figures 2, 3; Supplementary Tables S6–S9). Actinomycetota were more abundant in moraine samples (49% of the total read counts in moraines) compared to the other bedrock types (ranging from 9% in marble soils to 12% and 16% in gneiss and granite samples, respectively). Cyanobacteriota were dominant in granite, gneiss and marble samples, being represented by Nostocaceae (granite and gneiss: 20% and < 1%, respectively), Phormidaceae (granite and gneiss: 4% and 2%, respectively) and Chroococcidiopsaceae (marble, granite and gneiss: 31%, 4% and 17%). Cyanobacteriota were present in moraine soils as well, but less abundant compared to the other substrate types (3% in moraine) while Patescibacteria were more abundant in the moraine soils (2.6%), compared to marble (0.2%), gneiss (0.1%) and granite (0.8%). Metazoa (mainly Rotifera and Tardigrada) were abundant in marble (33%), gneiss (31%) and granite (24%). Rotifera were also abundant in moraine soils of Yûboku-dani Valley (25%). Together with other unclassified Metazoa, Rotifera were also found in the extreme soils of the Dry Valley (unclassified: 4%; Rotifera: 2%) and of Austkampane (unclassified: 5%). Other abundant eukaryotic taxa were Ciliophora (granite: 11%; marble: 4%; moraine: 3%; gneiss: < 1%), Cercozoa (marble and moraine: 14%; gneiss: 3%; granite: 1%) and Lobosa (moraine: 4%; granite, gneiss and marble: < 1%).

**Figure 4:**
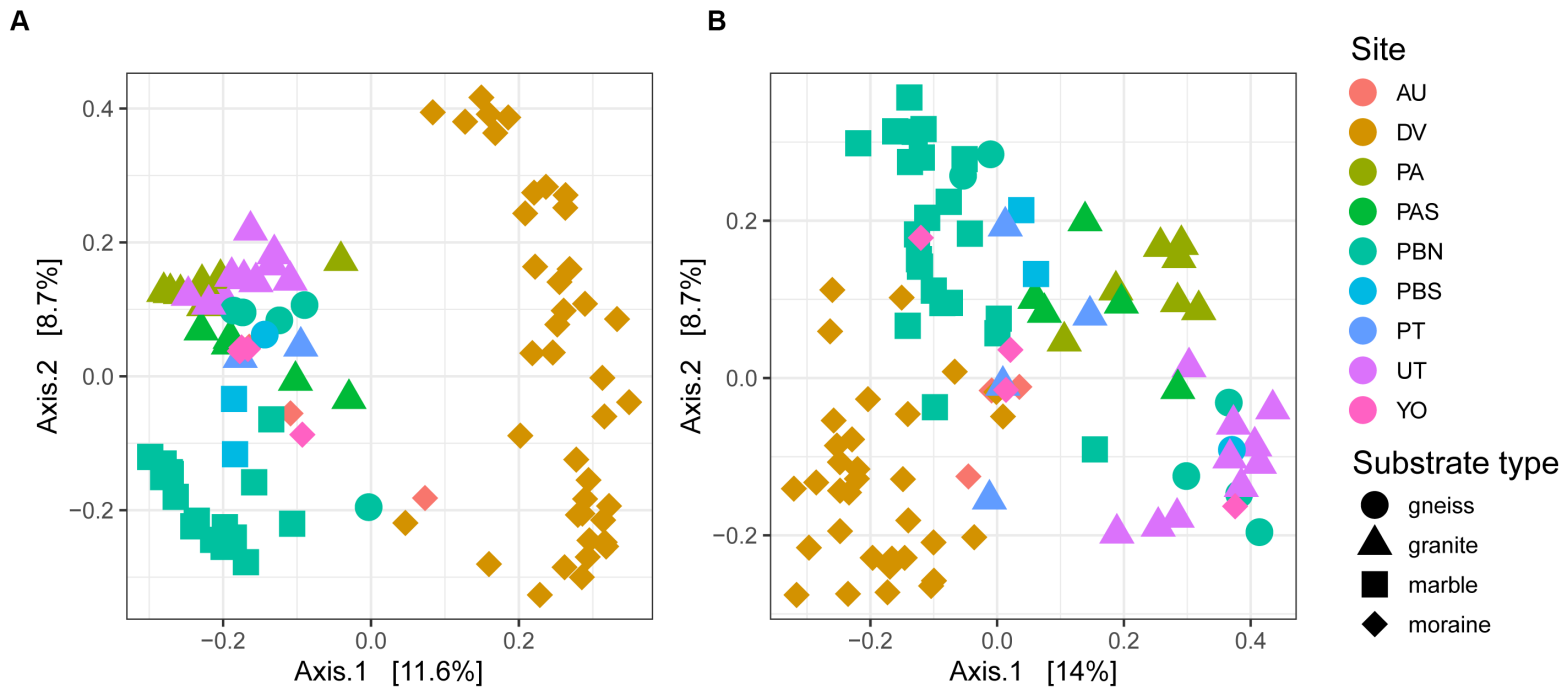
PCoA of 96 and 97 samples of **(A)** bacteria and **(B)** eukaryotes. Circles represent gneiss, triangles granite, squares marble and diamonds moraine samples. The percentage explained by the first two axes is given between brackets.

A PCoA on ASV level confirmed that samples from different sampling locations with the same substrates tended to have similar bacterial and eukaryotic communities (Figure 4). Granitic samples from the Pingvinane and Petrellnuten nunataks (PA and PT regions, respectively) and Utsteinen ridge (UT region) clustered together. Similarly, bacterial and eukaryotic communities on the marble soils from the two nunataks of Perlebandet (PBN and PBS) also clustered together. However, gneiss bedrock from Perlebandet had communities that were more similar to those observed in samples from granite bedrock. The Dry Valley (DV) bacterial communities appeared to be rather unique (Figure 4A) compared to the other sites. With a few exceptions, this was also the case for the eukaryotic communities in the Dry Valleys (Figure 4B).

### Community structure in relation to environmental factors, location and substrate type

Distance-based redundancy analysis (db-RDA) of the ASV relative abundance data showed a significant effect of environmental factors (*p* = 0.001), PCNMs (*p* = 0.001) and bedrock type (*p* = 0.001) on the microbial community structure. Best-fit environmental models showed that the combined environmental variables (pH, elevation, total nitrogen, conductivity, total phosphorus, ammonia, nitrates, total organic carbon and only for bacteria dry weight) accounted for 19% and 21% of the total variation in bacterial and eukaryotic community structure, respectively. Overall, four key factors (elevation, pH, total nitrogen and conductivity) had the most influence on the community structure, explaining together 14% and 16% of the total variation in bacterial and eukaryotic community structure, respectively, while all other environmental factors individually explained 1% or less of the total variation (Supplementary Tables S4, S5). Of the selected environmental properties, pH was the variable explaining the highest variation (7%). In addition, elevation appeared to be an important structuring factor for both bacteria and eukaryotes (5%). Overall, nearly the same geographical distance-based and bedrock type variables were selected for both datasets (Supplementary Tables S4, S5). Out of the four selected Principal Component of Neighborhood Matrix (PCNM) vectors built using (x,y) geographic coordinates, four and three PCNMs (PCNM3, PCNM4, PCNM1, PCNM2 and PCNM3, PCNM4, PCNM1) explained 10% and 11% of the total variation in bacterial and eukaryotic community structure, respectively. Selected scales reflect both short (PCNM3, PCNM4) and large (PCNM1, PCNM2) spatial scale differences and similarities between communities. Finally, three significant substrate type factors, namely moraine, marble, and gneiss, explained 16 and 14% of the total difference in bacterial and eukaryotic community structure, respectively.

### Network analysis

**Figure 5:**
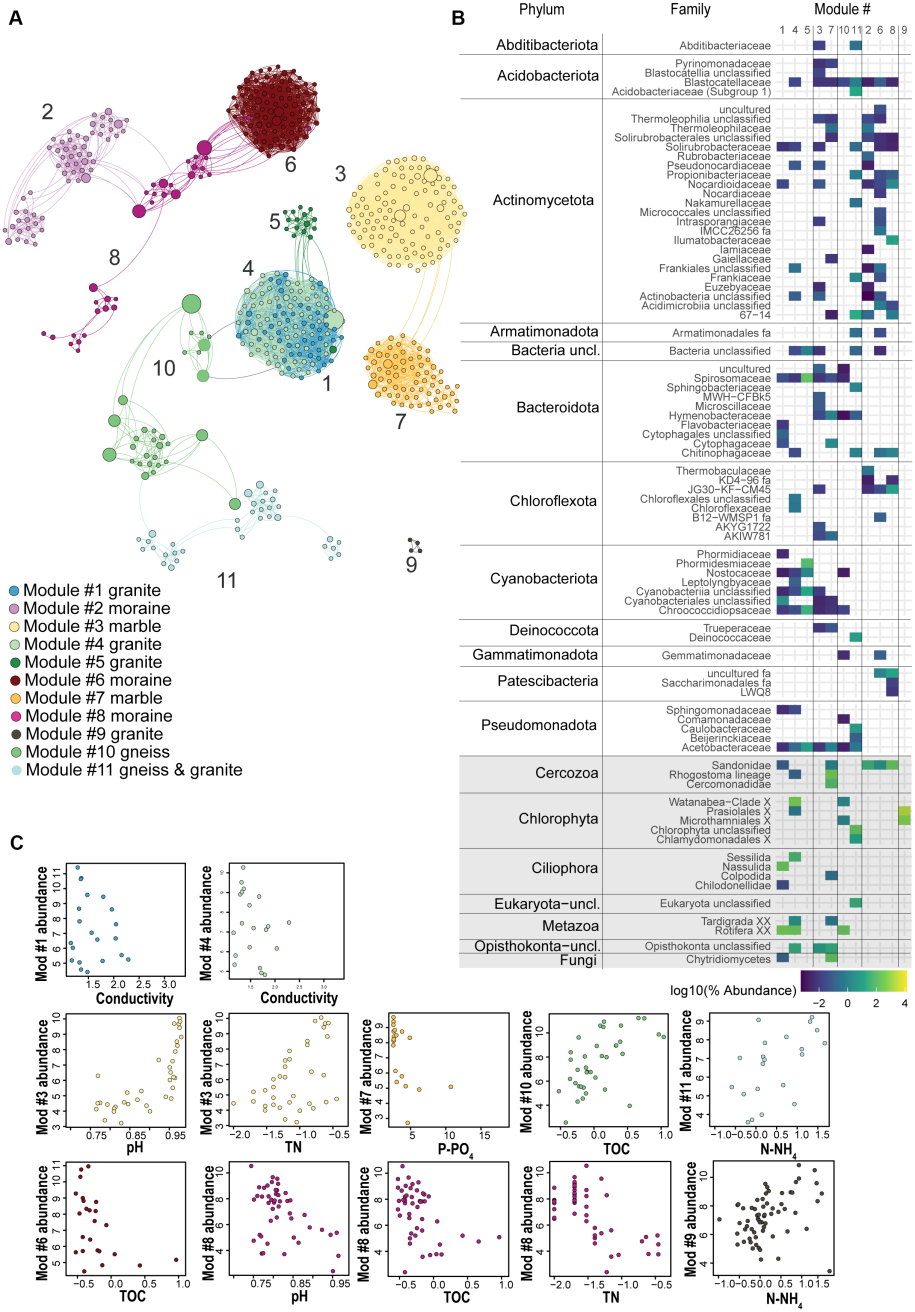
**(A)** Co-occurrence network analysis based on bacterial and eukaryotic ASVs (dots) across all sampling sites, colored according to the major identified modules in the different substrate types, with positive Spearman correlations > 0.7 plotted as edges. Size of nodes corresponds to the betweenness centrality degree. **(B)** Phylum-or Division-level taxonomic log-transformed abundance of those ASVs associated with each of the 11 modules, colors represent the abundance of ASVs per module, grey rectangles highlight eukaryotic phyla. **(C)** Relative abundance (*z*-score) of ASVs in each module plotted against the most important substrate geochemical variables identified by RF, Spearman correlation and Pearson correlation as the major explanatory variable for that module (*p* < 0.01, Holm-corrected). Module 5 represented a low number of samples. Modules 2 and 6 correlated well with decreasing TN values, similar to the plotted module 8.

A co-occurrence network enabled us to identify modules of strongly co-occurring ASVs which might represent co-existing soil taxa. Based on our criteria (ASVs relative abundance > 0.1%; global abundance > 5 reads; occurrence > 3 samples), only 4% of the bacterial ASVs (1,063 out of 27,398 ASVs) and 12% of the eukaryotic ASVs (205 out of 1,696) were kept for the network analyses (Figure 5A; Supplementary Figure S8). However, they accounted for 67.5 and 93.8% of 16S rRNA and 18S rRNA gene sequences across all samples, respectively. Together, our results suggest that soil bacterial as well as micro-eukaryotic communities, are typically dominated by a relatively small subset of ASVs. The final network included 552 nodes (91% of bacteria and 9% of eukaryotic ASVs) with 5,181 edges, and had an average clustering coefficient of 0.669 and an average diameter of 18.4 edges. Within the 5,181 edges, 4,675 indicated bacteria-bacteria co-occurrences, 452 bacteria-eukaryote co-occurrences and 76 eukaryote-eukaryote co-occurrences. Using this approach, we identified 11 modules of co-occurring ASVs (Figure 5; Supplementary Tables S11–S13), comprising multiple taxa (Supplementary Figure S9) and which were divided in 3 main hubs. One hub was composed of five connected modules (1, 4, 5, 10 and 11). The relative abundances of ASVs present in Modules 1, 4 and 5 were highest in granitic soils (Supplementary Figure S10), while in Module 10 this was in gneiss, and in Module 11 in both granitic and gneiss bedrock types. A second hub was composed of two modules, namely Modules 3 and 7, of which the ASVs were mostly abundant in marble soils. The third hub included three connected modules (Modules 2, 6 and 8) with ASVs mostly abundant in moraine bedrock type. Finally, Module 9 was isolated from the three hubs and encompassed only eukaryotic ASVs (Chlorophyta) which were mostly dominant in granitic soils, but also present in all the other substrate types (Supplementary Figure S10; Supplementary Table S12). Overall, Actinomycetota and Cyanobacteriota were the most abundant phyla, representing 27% and 24%, respectively, of the network nodes, whereas Chlorophyta, Cercozoa and Metazoa were the most represented eukaryotic phyla, accounting for 4, 2 and 1% of the network nodes (Supplementary Table S12). According to the bedrock types, different microbial consortia were observed (Supplementary Figures S6, S7). Photoautotrophs such as filamentous Cyanobacteriota and/or Chlorophyta were often dominant in granitic and gneiss soils (representing between 15% and 85% of the total reads of Modules 1, 4, 5, 10 and 11) whereas the eukaryotic heterotrophs Ciliophora and Metazoa were the second most abundant category in these modules. Ciliophora dominated Module 1 (Nassulida), accounting for 56.2% of its total read count and represented 5% in Module 4 (Sessilida), whilst Metazoa (Rotifera) accounted for 15%, 13% and 9% of the total reads count of Modules 1, 4 and 10. Other bacterial phyla were represented by Pseudomonadota (6% and 8% of the total read counts of Modules 5 and 11, respectively), Bacteroidota (9% and 5% of the total reads count of Modules 5 and 11, respectively) and Actinomycetota (5% and 7% of the total reads count of Modules 4 and 11, respectively; Supplementary Tables S11, S12). Moraine modules 2, 6 and 8 were dominated by the bacterial Actinomycetota phylum (75%, 67% and 27%, respectively; Supplementary Tables S11, S12) followed by Acidobacteriota (11% in M2 and M8) or Chloroflexota (16% in M6) and the eukaryote Cercozoa division which dominated M8 (54%; Supplementary Tables S11, S12) and was less present in M2 (13%) and M6 (2%).

The nodes whose betweenness centrality and vertex degree indices were the highest for each module were considered as representing putative keystone taxa. Among the granitic and marble modules, Cyanobacteriota (of the Nostocaceae and Chroococcidiopsaceae families, respectively) were identified as keystone taxa candidates. This is consistent with granitic and gneiss modules sharing a putative keystone taxon of the phylum Pseudomonadota, a common cyanobacteria predator. The ASV representative was predicted to belong to the Sphingomonadaceae family showing the highest betweenness centrality of the granitic module 4 (as well as of the whole network). Also, the nitrogen-fixer *Rhizobacter* was considered a putative keystone taxon in the gneiss module 11 (Supplementary Table S17). Gneiss Module 10 and gneiss & granitic Module 11 shared putative keystone taxa such as Acidomycetota (*Blastocatella*) and Chlorophyta (*Trebouxia jamesii* in Module 10 and unclassified representatives in Module 11), whereas Module 10 was also characterized by Cyanobacteriota (*Nostoc*) despite its very low abundance in the module (only 0.6%, Supplementary Table S11). Granitic modules correlated with decreasing conductivity values (M1 and 4), or total N concentration (M5; Figure 5; Supplementary Table S16). Gneiss Module 10 correlated with increasing TOC concentrations whilst gneiss & granitic Module 11 correlated with increasing TOC, N-NH_4_^+^ and TN concentrations (Figure 5; Supplementary Table S16). Although the ASVs of Module 3 were more abundant in “pure” marble samples, the ASVs of Module 7 were more abundant in marble soils that had some gneiss intrusions. Both marble modules correlated with increasing pH values and decreasing P-PO_4_^3−^ values, whilst Module 3 also correlated with increasing total N (Supplementary Table S16). Putative keystone taxa in moraine modules were represented mainly by Actinomycetota (*Crossiella* and 67–14 in Module 2; Solirubrobacterales in Modules 6 and 8), Chloroflexota (JG30-KF-CM45) in Module 6 and Patescibacteria (uncultured) in Module 8 (Supplementary Table S17). Module 2, 6 and 8 correlated best with decreasing total N concentrations, although Modules 6 and 8 were also inversely correlated with decreasing pH values and TOC concentrations (Supplementary Table S16).

## Discussion

Our findings indicate, as hypothesized, that the composition of terrestrial bacteria and micro-eukarya of the inland ice-free areas of the Sør Rondane Mountains (East Antarctica) largely co-vary with the four substrate types, namely granite, gneiss, marble and moraine, even at meter-scale distances as evidenced by the different communities being present on gneiss and marble in Perlebandet. This is in agreement with Tytgat et al. (2016) who observed significant differences in bacterial community structure between granite and gneiss substrates in the same Antarctic region. The influence of substrate was also described by Lulákova et al. (2023) who showed differences in edaphic microbial communities from distinct mineral substrates in two adjacent forefield glaciers in Svalbard, but also by Meslier et al. (2018) and Archer et al. (2016) who found differences in endolithic communities occurring in hot and cold deserts. In the present study, we show that both bacterial and eukaryotic communities of marble and moraine soils are even more distinct compared to those of granite and gneiss. Indeed, three disconnected ecological clusters (modules) could be discerned in our co-occurrence network analysis (i.e., granite-gneiss, marble and moraine) in which putative and well known primary producers were predicted as the main keystone taxa that co-occurred with different other bacterial and eukaryotic taxa. These ecological modules correlated with important physical and chemical parameters characterizing the substrate types. This is very likely because substrate type may directly affect soil chemistry (e.g., the presence of weathering products, pH and ions and nutrients concentration), as well as other unmeasured environmental parameters at micro- and macro-scale such as soil temperature and the water retention capacity. Other factors such as elevation were instead probably masking a site effect (i.e., moraines of Dry Valley, the highest sampling site). In turn, particular soil biota may also alter micro-habitat conditions by, for example, providing a habitat and buffer against environmental extremes (e.g., soil crusts producing EPS), altering the concentration and form of nutrients, and producing organic carbon for heterotrophs and mixotrophs (e.g., Belnap et al., 2001; Colesie et al., 2013; Pessi et al., 2019). The diversity of particular biota may therefore impact the diversity and abundance of other members of soil food webs via trophic interactions. Below we discuss the main co-occurrence modules based on the dominance of particular taxa and or the presence of ASVs that might represent keystone species. A keystone ASV is considered as an important community member crucial for network stability and therefore highly correlated with other phylotypes (highest vertex degree) and/or connects with other ASVs being present in different hubs (highest betweenness centrality) (Martín González et al., 2010; Vick-Majors et al., 2014; Banerjee et al., 2016).

### The filamentous Nostocaceae family (Cyanobacteriota) is abundant on granite

Granite hosted the highest relative abundance of diazotrophic phylotypes of the filamentous Nostocaceae family (20% of bacterial relative abundance), which is consistent with the observations by Tytgat et al. (2016) in the same region. Nostocaceae phylotypes are capable of producing an exopolysaccharide (EPS) matrix, which could result in more favorable micro-environmental conditions for other bacteria and micro-eukaryotes (Tamaru et al., 2005). Nostocaceae phylotypes were designated as putative keystone taxa of the granitic modules 4 and 5. Notably, also an ASV of *Polymorphobacter* (Pseudomonadota, Sphingomonadaceae family) was defined as a putative keystone phylotype of the granitic Module 4 because of its high betweenness centrality (the highest of the entire network). Interestingly, this ASV is not dominant in abundance and belongs to one of the least abundant families in all the granitic samples. Nostocaceae and Sphingomonadaceae are known to be nitrogen-fixing families (Coyne et al., 2020), and thrive under oligotrophic stress, which may explain why their presence, though in low abundance, may be critical in sustaining life. The characteristic eukaryotes on granite substrate were Metazoa and Ciliophora. For Metazoa, these were mainly Rotifera and Tardigrada, which, together with Ciliophora, are taxa commonly observed in Antarctic desert soils, as well as terrestrial and lacustrine habitats in more coastal regions of the continent (Obbels et al., 2016; Thompson, 2021). In addition to these characteristic taxa, the bacterial Acidobacteriota and the photoautotrophic eukaryotic Chlorophyta (especially of the Microthamniales order) phyla were abundant in granite soils, but also on the other substrate types, which is in agreement with other studies (Moniz et al., 2012; Obbels et al., 2016), with Chlorophyta considered pioneers in the colonization of soils in the Antarctic (Wynn-Williams, 1996). Acidobacteriota constitute one of the most abundant phyla dominating bacterial communities in all kinds of soils (Lee, 2009; Delgado-Baquerizo et al., 2018a) including in Antarctica (Tytgat et al., 2016; Lambrechts et al., 2019), and contain phylotypes able to be active at low temperatures and resilient to multiple freeze–thaw cycles (Männistö et al., 2011). Additionally, Acidobacteriota have been shown to harbor the ability to oxidize atmospheric H_2_, which might represent a selective advantage enabling them to survive periods of carbon depletion (Giguere et al., 2021; Ortiz et al., 2021).

### The unicellular Chroococcidiopsaceae family (Cyanobacteriota) dominates marble

As on the granitic bedrock, Cyanobacteriota also dominated communities on marble substrates. Here, however, the dominant phylotypes belonged to the unicellular Chroococcidiopsaceae family. Species of the Chroococcidiopsidales order (e.g., *Chroococcidiopsis* sp., *Aliterella chasmolithica*, *Gloeocapsopsis dulcis*) are known to resist prolonged periods with moisture deficit and UVR as a result of the conspicuous production of compatible solutes along with EPS or thick mucilage around the cells (Hershkovitz et al., 1991; Potts, 1994; Jung et al., 2020; Moore et al., 2023). Members of the Chroococcidiopsaceae are often found to dominate lithic habitats of both hot and cold deserts (Broady, 2005; Pointing et al., 2009; Chan et al., 2012; Jung et al., 2020) and especially in quartz-derived rocks which enable UV filtering but sufficient PAR to reach the biota. After our co-occurrence network analysis, we identified several phylotypes of the *Aliterella* genus (Chroococcidiopsaceae) as putative keystone taxa in the marble modules. This highlights not only the dominance of this family in this alkaline substrate type but also their important ecological role in the community. *Aliterella* has recently been described (Rigonato et al., 2016) and only a few strains have been isolated and studied so far. Other studies focusing on marble bedrock (i.e., Timoncini et al., 2022)—despite their very distant focus compared to our study (i.e., bacterial composition of marble statues)—also found a predominance of cyanobacteria belonging to the genus *Aliterella*. These highly specialized cyanobacterial communities could also be lichen-forming. Indeed, two of the recently described *Aliterella* species were cyanobionts (*A. compacta* and *A. gigantea*) of different fungal species with which they form lichens (*Peltula clavata* and *Peltula capensis*, respectively; Jung et al., 2020) in the Atacama Desert. Interestingly, in our marble samples, black lichen communities were often present, which may suggest that the phylotypes occurring in our study may form symbionthic lichen communities as well. However *Peltula* genus was not encountered in our dataset. Whether this genus is linked to the high alkalinity, the calcium carbonate or other features of marble, however, need further research. In addition to Cyanobacteriota, the phylum Bacteroidota was mostly abundant in marble soils, which is consistent with other studies (e.g., Lauber et al., 2009) in which Bacteroidota were found to be abundant in other alkaline soils. In marble, we measured pH values of up to 9 in Perlebandet. For bacteria, differences in soil pH may help explain the variation in their diversity and structure (Fierer and Jackson, 2006), and for example, they could explain why different families of Cyanobacteriota are abundant in granite and marble. As in granite substrates, Chlorophyta and Metazoa (Rotifera and Tardigrada) were the dominant eukaryotes of these marble soils. However, in contrast to the granite samples, Cercozoa were more abundant than Ciliophora, which is likely related to the dry conditions encountered in the marble soils, consistent with findings in soils from the McMurdo Dry Valleys (Thompson, 2021).

### The Acidobacteriota phylum dominates gneiss

On gneiss, the Acidobacteriota were the dominant bacteria (Supplementary Table S5). Despite its high abundance in edaphic habitats of Antarctica (Lee et al., 2012; Van Horn et al., 2014; Tytgat et al., 2016), this bacterial phylum has been detected in great abundance in the inactive fraction of Antarctic soil microbial communities (Schwartz et al., 2014; Buelow et al., 2016). Cyanobacteriota was the second most abundant bacterial phylum in gneiss, with a high abundance of members of the unicellular Chroococcidiopsaceae family. Interestingly, ASV1328, here identified as closely related to the *Nostoc* sp. PCC-73102 strain, was identified as putative keystone taxon of gneiss Module 10 despite its low abundance in gneiss samples (Nostocaceae family). Because of their ability to fix atmospheric N_2_, this phylum might contribute to the N cycle of gneiss food web. The eukaryotic communities were again dominated by Chlorophyta and among the Metazoa, Rotifera and Tardigrada were dominant groups, supporting our hypothesis concerning the abundance of Metazoa phylotypes in presence of a high abundance of Cyanobateriota ones.

### The Actinomycetota phylum dominates moraines

Moraine soils, especially from Austkampane and the Dry Valley, were the most arid and oligotrophic among the investigated substrates. Moraines are characterized by a mixture of different bedrock types. Actinomycetota were found to be conspicuously more abundant than photoautotrophs in moraine substrates, with abundances up to 50% of the bacterial reads, which is consistent with studies from other hyper-arid regions of Antarctica (Bay et al., 2021; Ortiz et al., 2021; Ray et al., 2022), other cold deserts across the globe (An et al., 2013; Gupta et al., 2015) as well as hot deserts (see Raimond and Cowan 2022 and references therein). Species of this phylum are known to be able to outcompete other groups under the most arid conditions in oligotrophic soils, likely due to their high resistance to desiccation and starvation conditions (Battistuzzi and Hedges, 2008; Jones and Lennon, 2010). They are furthermore metabolically active at subzero temperatures and form cyst-like resting structures or spores (Soina et al., 2004). They are also able to withstand very high UV radiation intensity thanks to their numerous UV repair mechanisms, enabling them to survive on the soil surface (Makhalanyane et al., 2015). The majority of the investigated moraine samples were taken from a high elevation site in the southern continental area (i.e., Dry Valley), which were therefore exposed to harsh conditions with continuous strong winds, in contrast to edaphic niches of, e.g., nunataks, characterized by a higher number of sheltered sites, even produced by the nunatak’s slope against the wind. This means that the structure found in the microbial communities from the moraine soils could also be explained by other unmeasured intrinsic parameters of the Dry Valley site. For instance, Dry Valley was the highest sampled site (1,600–1,700 m a.s.l.), which probably contributes in explaining why elevation was one of the main environmental drivers of the observed microbial communities. The co-occurrence network analysis revealed three connected moraine modules, whose ASVs mainly belonged to the Pseudonocardiaceae, Solirubrobacteraceae, Frankiales, and Nocardioidaceae families (all Actinomycetota) and whose putative keystone taxa belonged to the Actinomycetota and the Chloroflexota phyla. Indeed, other highly abundant phylotypes of moraine substrates belonged to the Chloroflexota phylum, which is also often encountered in extremely arid and ultra-oligotrophic Antarctic environments (Ji et al., 2015). In such cyanobacteria-poor soils, part of the bacterial primary production might also be attributed to atmospheric H_2_, CO_2_ and CO consumption by phylotypes of the Actinomycetota (including the order Solirubrobacterales), Chloroflexota and other taxa (Ji et al., 2017; Ortiz et al., 2021; Greening et al., 2022; Ray et al., 2022). Surprisingly, even in these very dry soils, Chlorophyta were the dominant eukaryotes, together with Cercozoa. Interestingly, and seemingly in contrast to our second hypothesis, also Metazoans attained a considerable relative abundance (9%), with Rotifera and unclassified taxa being the most abundant groups. For Rotifera, this is probably because of their ability to produce a drought-resistant dormant stage (resting egg), which can remain viable for a long time, survive desiccation and frost, and is well adapted for passive dispersal (Artois et al., 2011). In addition, many soil bacteria are also estimated to often be in a dormancy state, and potentially even more so on Antarctic moraines (Jones and Lennon, 2010; Schwartz et al., 2014; Buelow et al., 2016; Feeser et al., 2018). However, while we cannot rule out that these are inactive organisms, the presence of Metazoa might imply that the primary production by these bacteria and the dry and cold adapted Chlorophyta is sufficiently high to support these relatively diverse communities.

## Conclusion

Our analyses revealed that the composition of edaphic bacterial and micro-eukaryotic communities in the inland ice-free areas of the Sør Rondane Mountains (East Antarctica) largely co-varies with substrate type. Three disconnected ecological clusters (modules) could be discerned in our co-occurrence network analysis (i.e., granite-gneiss, marble and moraine) in which putative and well-known primary producers were designated as the main keystone taxa which co-occurred with different other bacterial and eukaryotic taxa. These ecological modules were correlated with important physical and chemical parameters characterizing the different substrate types and, in some cases, location as well, suggesting that other unmeasured micro-climatic and geological features need to be further investigated. Our study provides new insights into the microbial ecology of terrestrial microbial communities in inland regions of the most extreme cold deserts on Earth. It also contributes to the creation and management of an Antarctic Specially Protected Area to protect the microbial diversity of representative sites in this region.

## Data availability statement

The datasets presented in this study can be found in online repositories. The names of the repository/repositories and accession number(s) can be found at: NCBI – PRJNA1044841: SAMN38413892–SAMN38413996.

## Author contributions

VS: Data curation, Formal analysis, Methodology, Software, Validation, Visualization, Writing – original draft, Writing – review & editing. SL: Formal analysis, Software, Validation, Writing – review & editing, Data curation, Methodology. BT: Conceptualization, Funding acquisition, Investigation, Methodology, Project administration, Software, Supervision, Validation, Writing – review & editing. QV: Funding acquisition, Validation, Visualization, Writing – review & editing. JE: Data curation, Methodology, Validation, Writing – review & editing. AWill: Conceptualization, Funding acquisition, Investigation, Project administration, Supervision, Validation, Writing – review & editing. AWilm: Funding acquisition, Investigation, Project administration, Validation, Writing – review & editing. EV: Conceptualization, Funding acquisition, Investigation, Methodology, Project administration, Resources, Supervision, Validation, Writing – review & editing. WV: Conceptualization, Funding acquisition, Investigation, Methodology, Project administration, Resources, Supervision, Validation, Writing – review & editing.
