# Supplementary material for: Geology defines microbiome structure and composition in nunataks and valleys of the Sør Rondane Mountains, East Antarctica
Source: Front Microbiol. 2024 Feb 6;15:1316633. doi: 10.3389/fmicb.2024.1316633 (PMC10877063; doi:10.3389/fmicb.2024.1316633)
Supplement: Supplementary file 1 [file Data_Sheet_1.docx]

1. **SUPPLEMENTARY FIGURES**

**
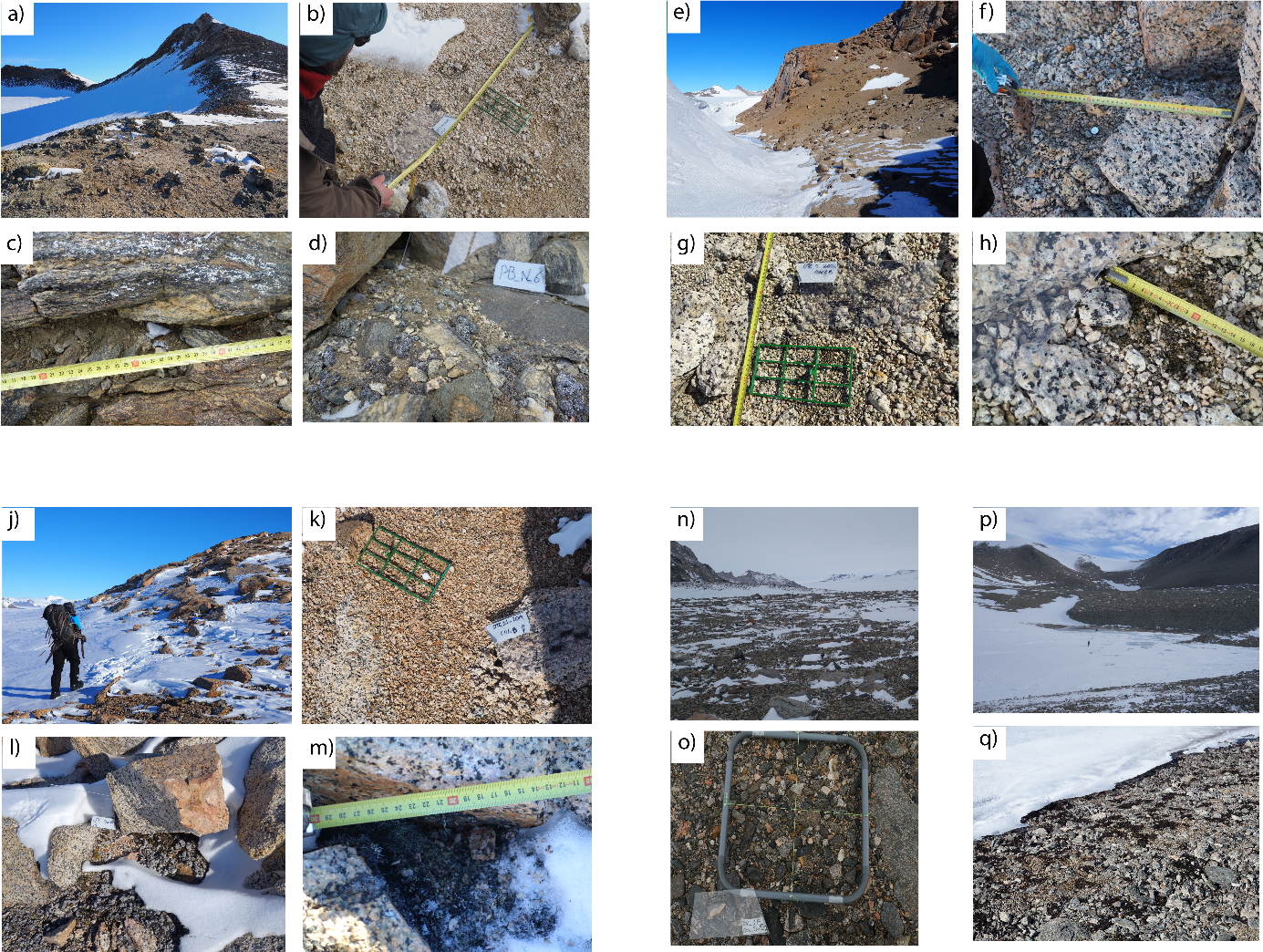
**

***Fig. S1 Detail of the sampled sites****. Perlebandet N nunatak overview (a) and zoom on marble (b) and gneiss (c-d) bedrock types. Pingvinane 6 nunatak overview (e) and zoom on its communities (f) and Pingvinane 4 nunatak (g-h) communities on granitic gravel. Utsteinen ridge overview (j) and zoom on bare granitic gravel (k) and developed BSCs (l-m). ‘Dry Valley’ overview (n) and zoom on its moraines (o). Yûboku-dani Valley overview (p) and zoom on moraines close to a frozen lake. More information about the samples can be found in Table S1.*


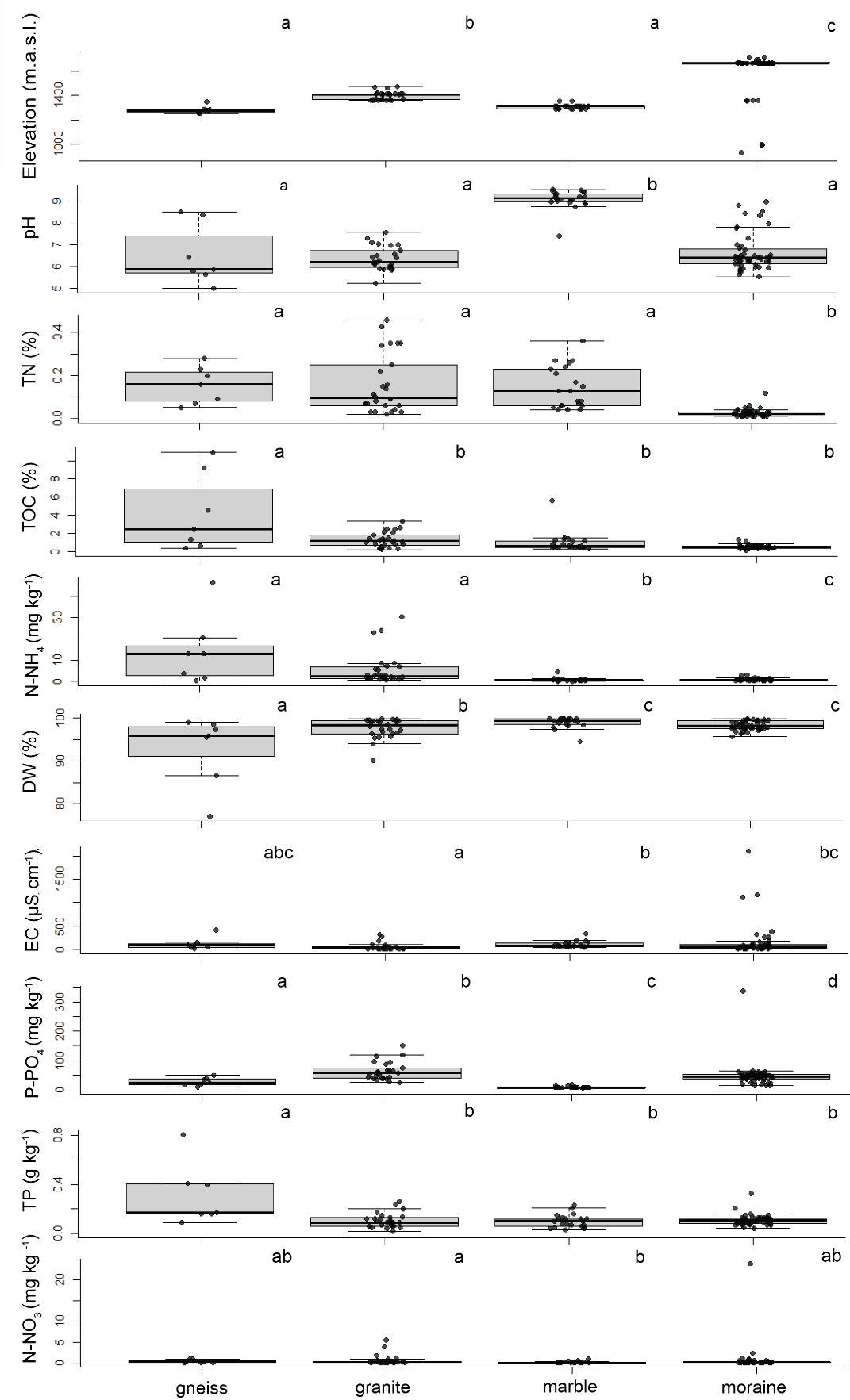


***Fig. S2*** ***Elevation and geochemical characterization of the soil ecosystems (four substrate types: gneiss, granite, marble and moraine).*** *Boxplots followed by different letters are significantly different (Kruskal-Wallis rank sum test, p < 0.05). Bars represent standard error.*

***
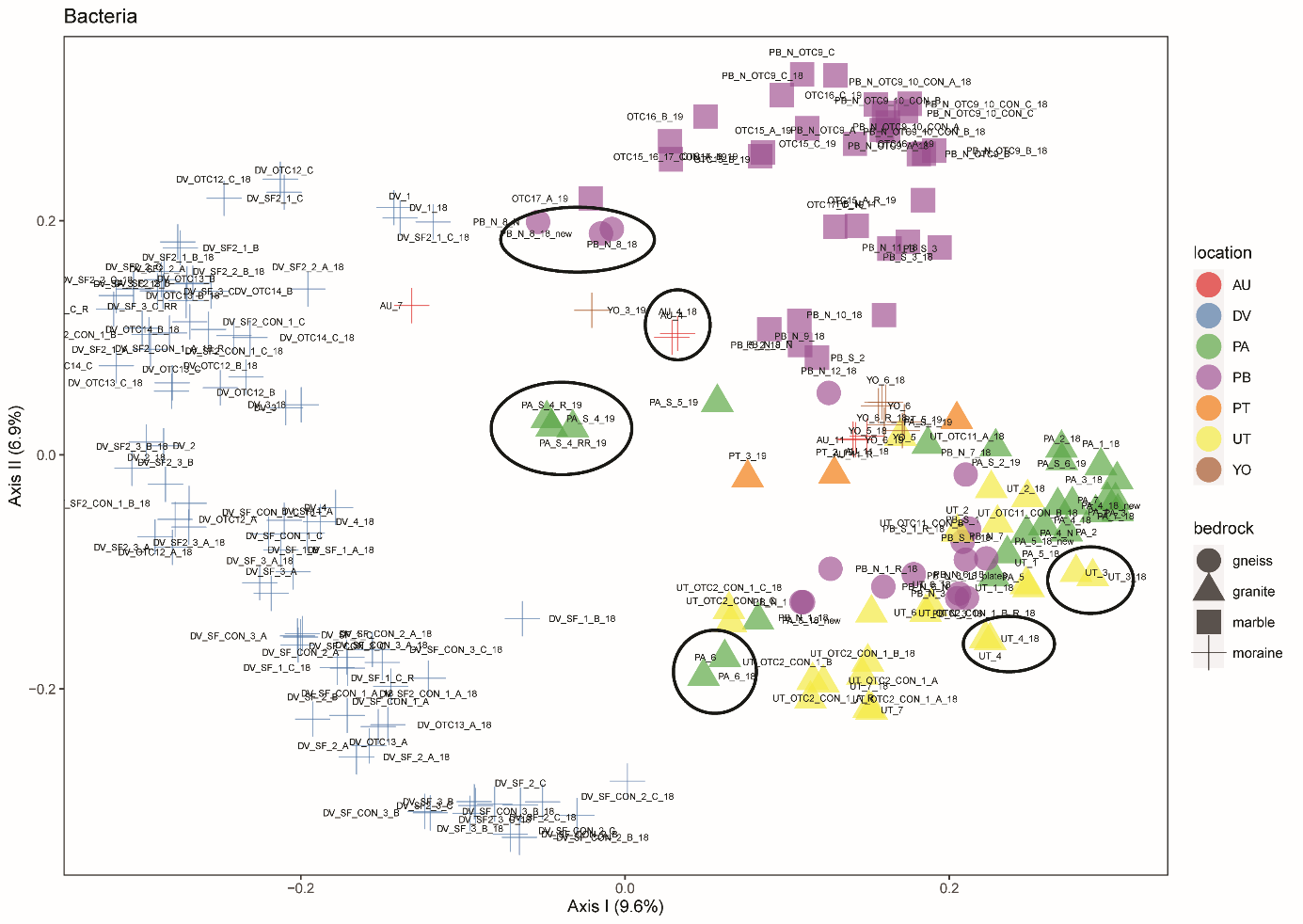
Fig. S3 PCoA of bacterial ASV table representing replicates*** *(encircled in black).*


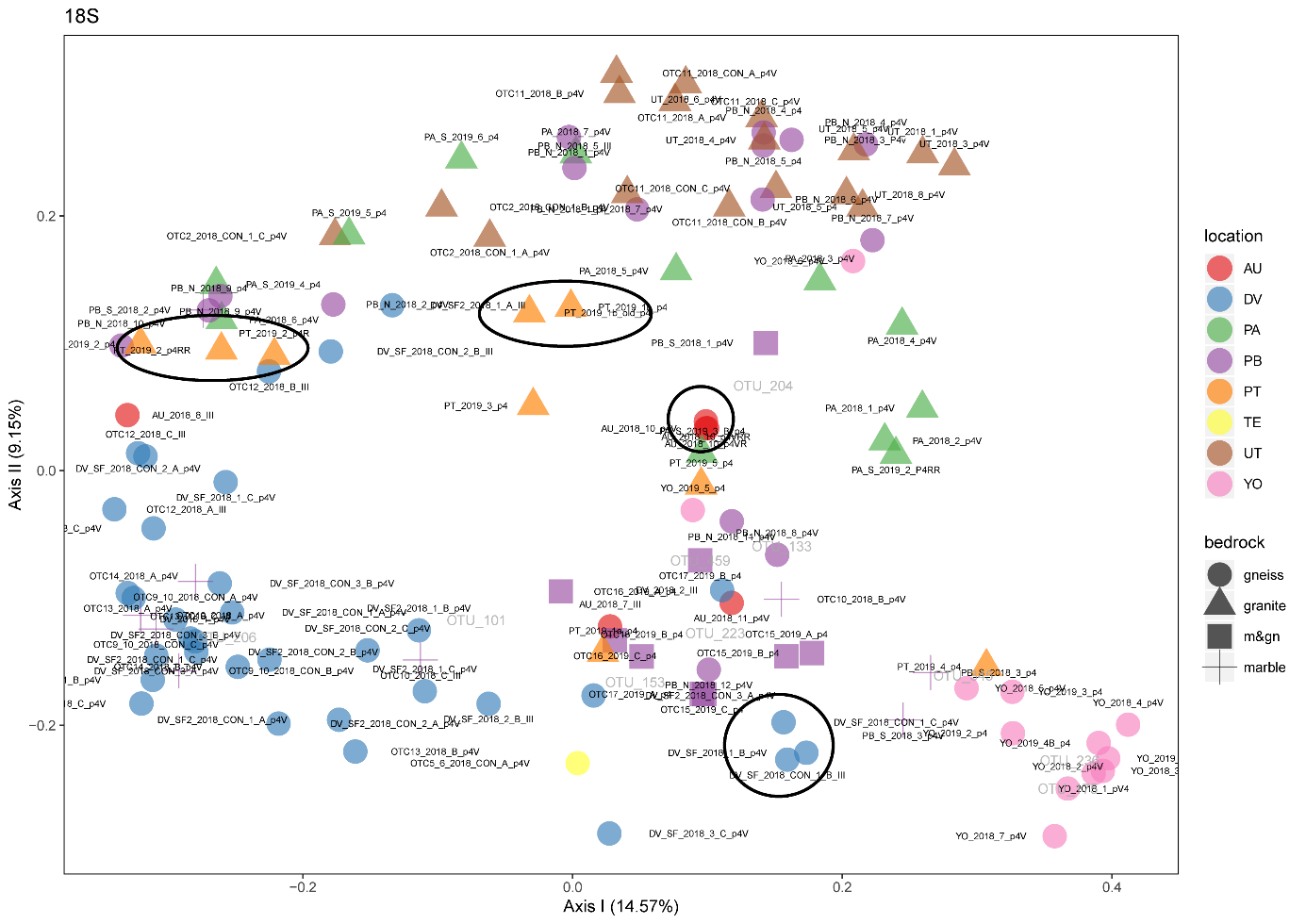


***Fig. S4*** ***PCoA of eukaryotic ASV table representing replicates*** *(encircled in black).*

**
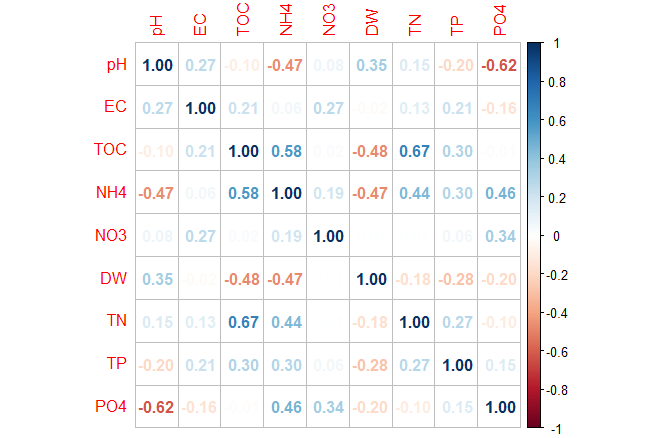
**

***Fig. S5 Correlogram showing Pearson’s correlation coefficients of all geochemical transformed variables.*** *EC: electrical conductivity; TOC: total organic carbon; DW: dry weight; TN: total nitrogen; TP: total phosphorus.*

***
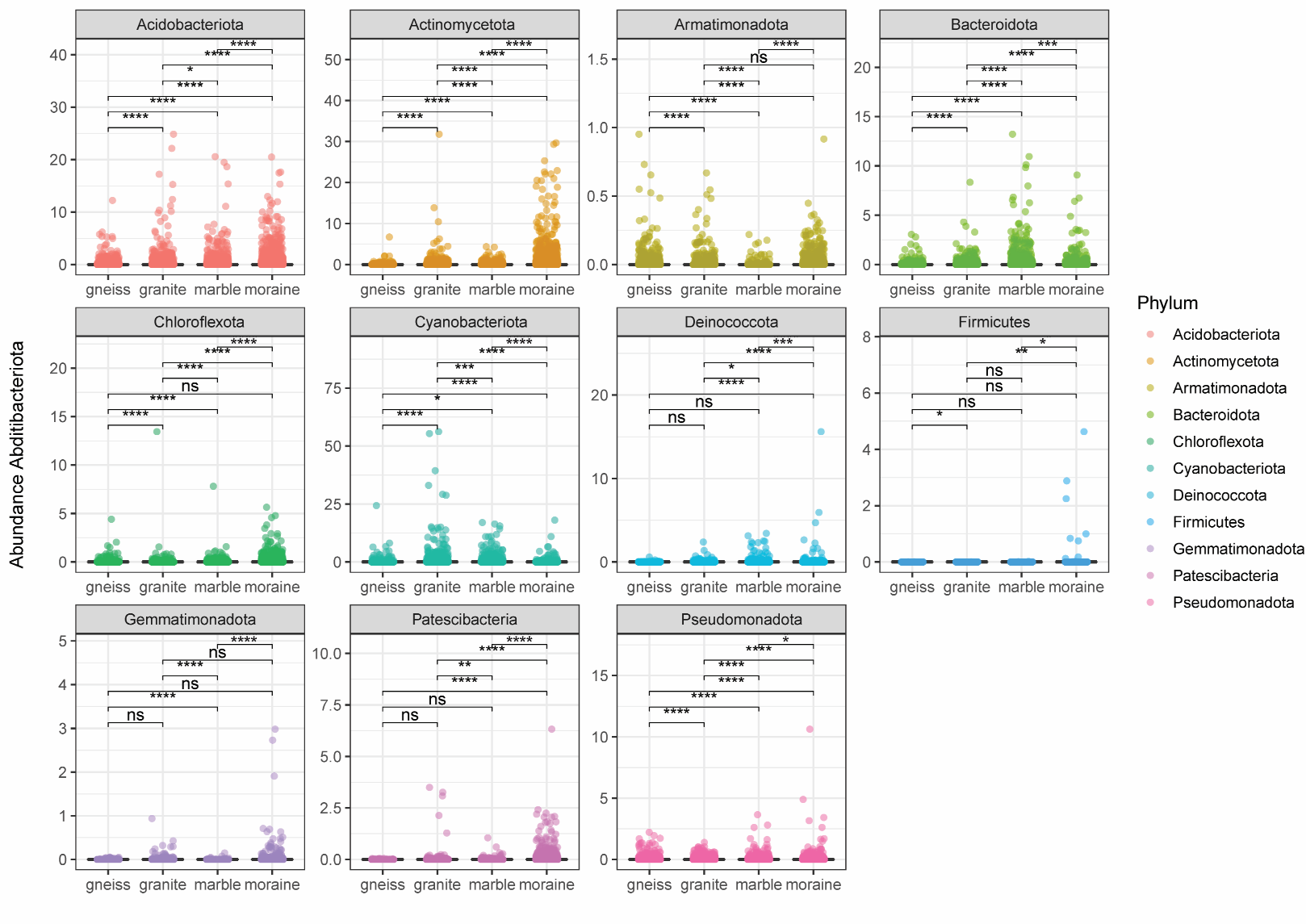
***

***Fig. S6 Boxplot of relative abundances (in %) of bacterial phyla in the different kinds of substrates.*** *Colors represent different phyla. Asterisks represent the significance of the p-value of the pairwise Wilcoxon test after mean comparison by the Kruskal Wallis test. Asteriks represent p-values: * = p < 0.05; ** = p < 0.01; *** = p < 0.001; ns = to p > 0.05.*

**
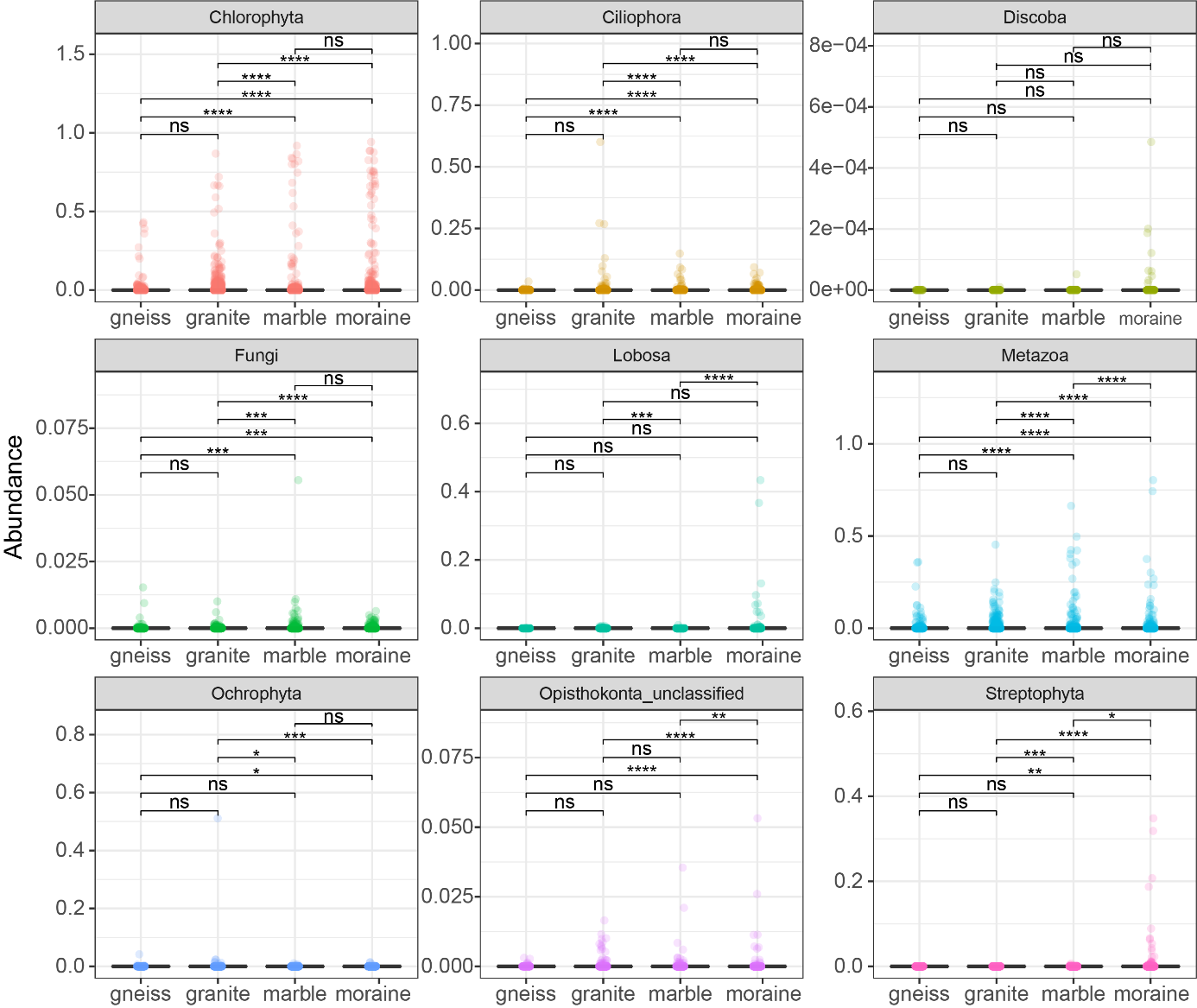
**

***Fig. S7 Boxplot of relative abundances (in %) of eukaryotic phyla in the different kinds of substrate.*** *Colors represent different phyla. Asteriscs represent the significance of the p-value (p < 0.05) of the pairwise Wilcoxon test after mean comparison by the Kruskal Wallis test. ns correspond to p > 0.05. Asteriks represent p-values: * = p < 0.05; ** = p < 0.01; *** = p < 0.001; ns = to p > 0.05.*

**
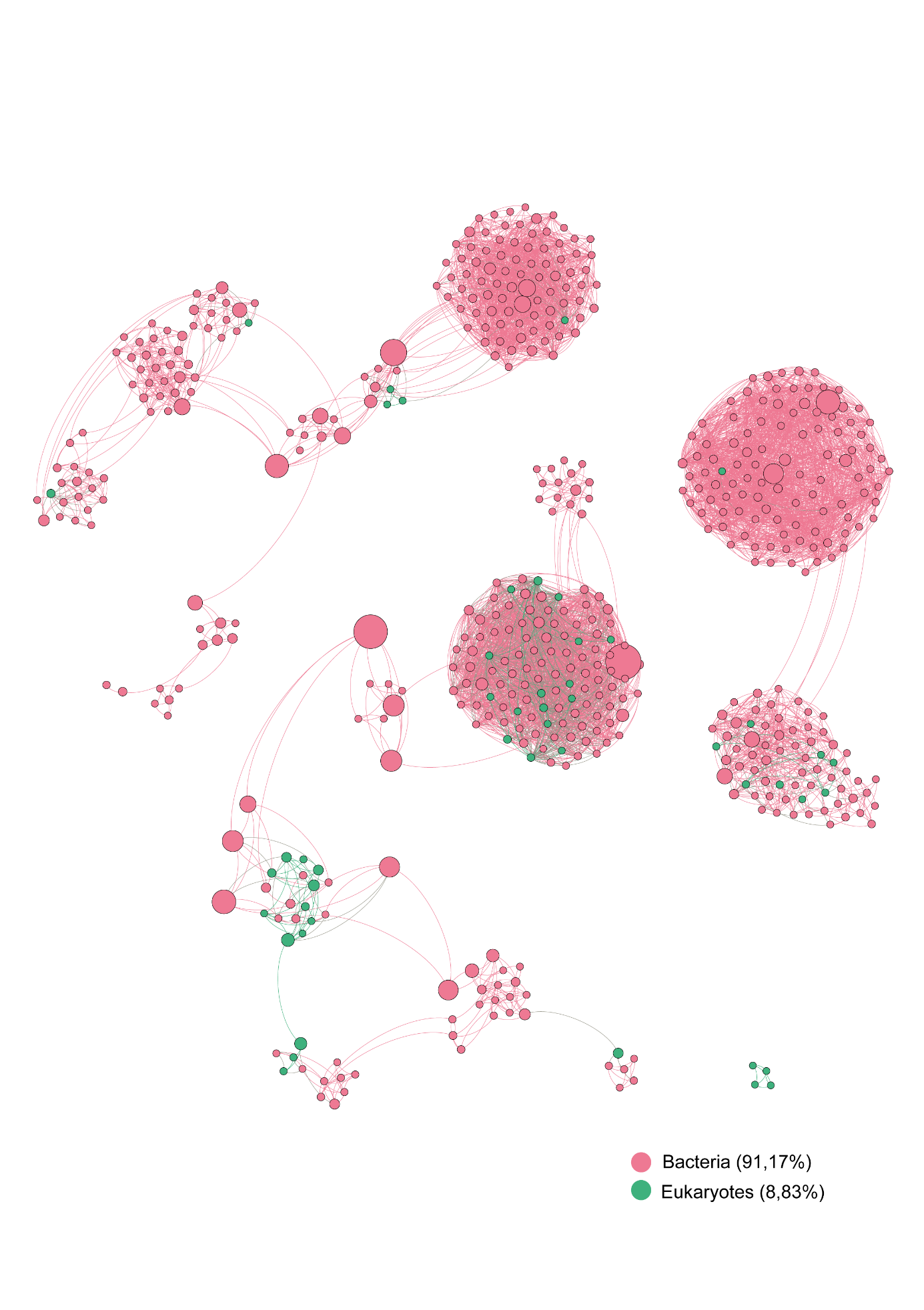
**

***Fig. S8 Co-occurrence network analysis based on bacterial and eukaryotic ASVs across all sampling sites****, colored by Kingdom (red: bacterial ASVs; green: eukaryotic ASVs). Size of nodes corresponds to the betweenness centrality degree.*

**
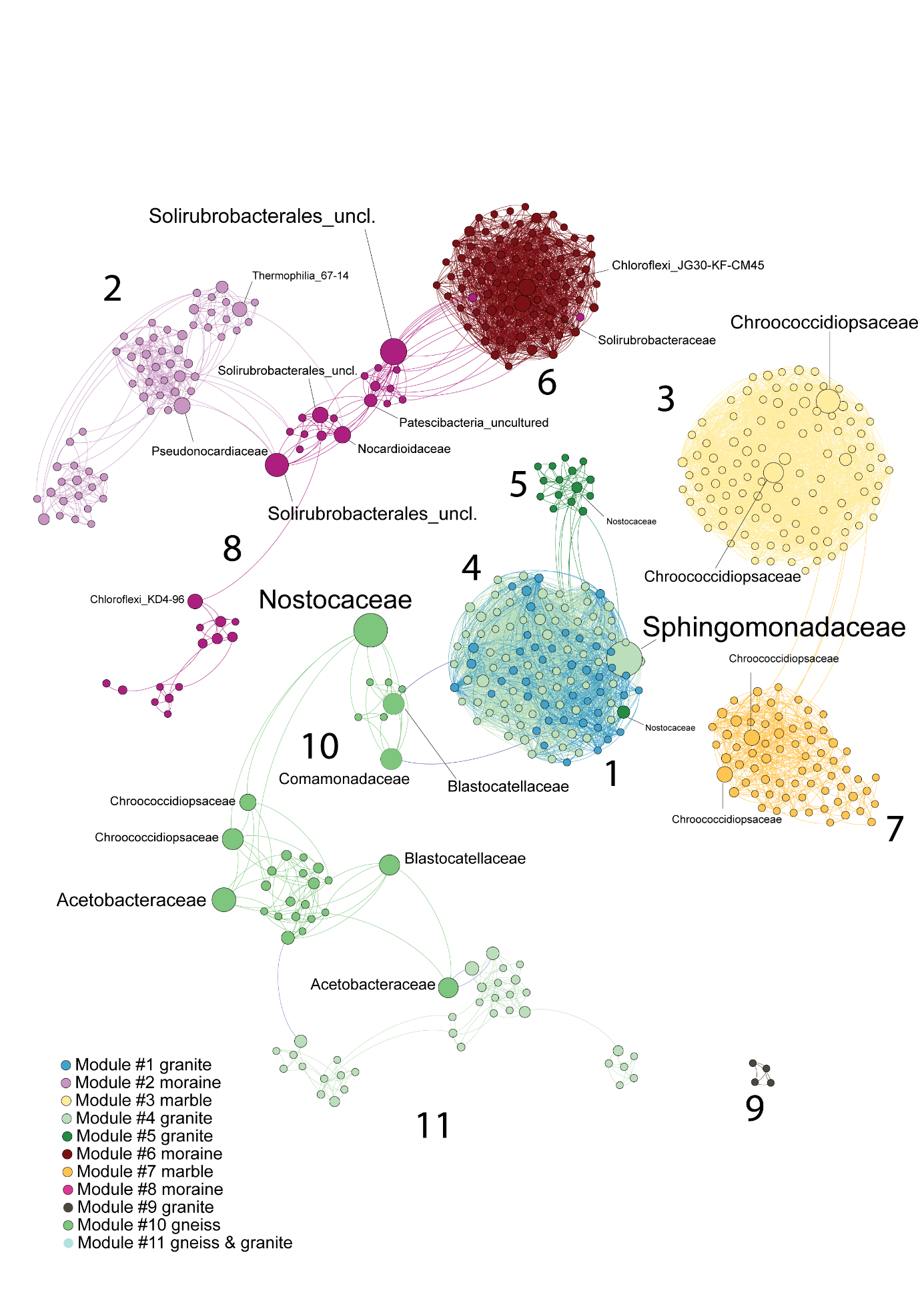
**

***Fig. S9 Co-occurrence network analysis based on bacterial and eukaryotic ASVs across all sampling sites****, colored by Modules (see legend). Size of nodes and label corresponds to the betweenness centrality degree.*

**
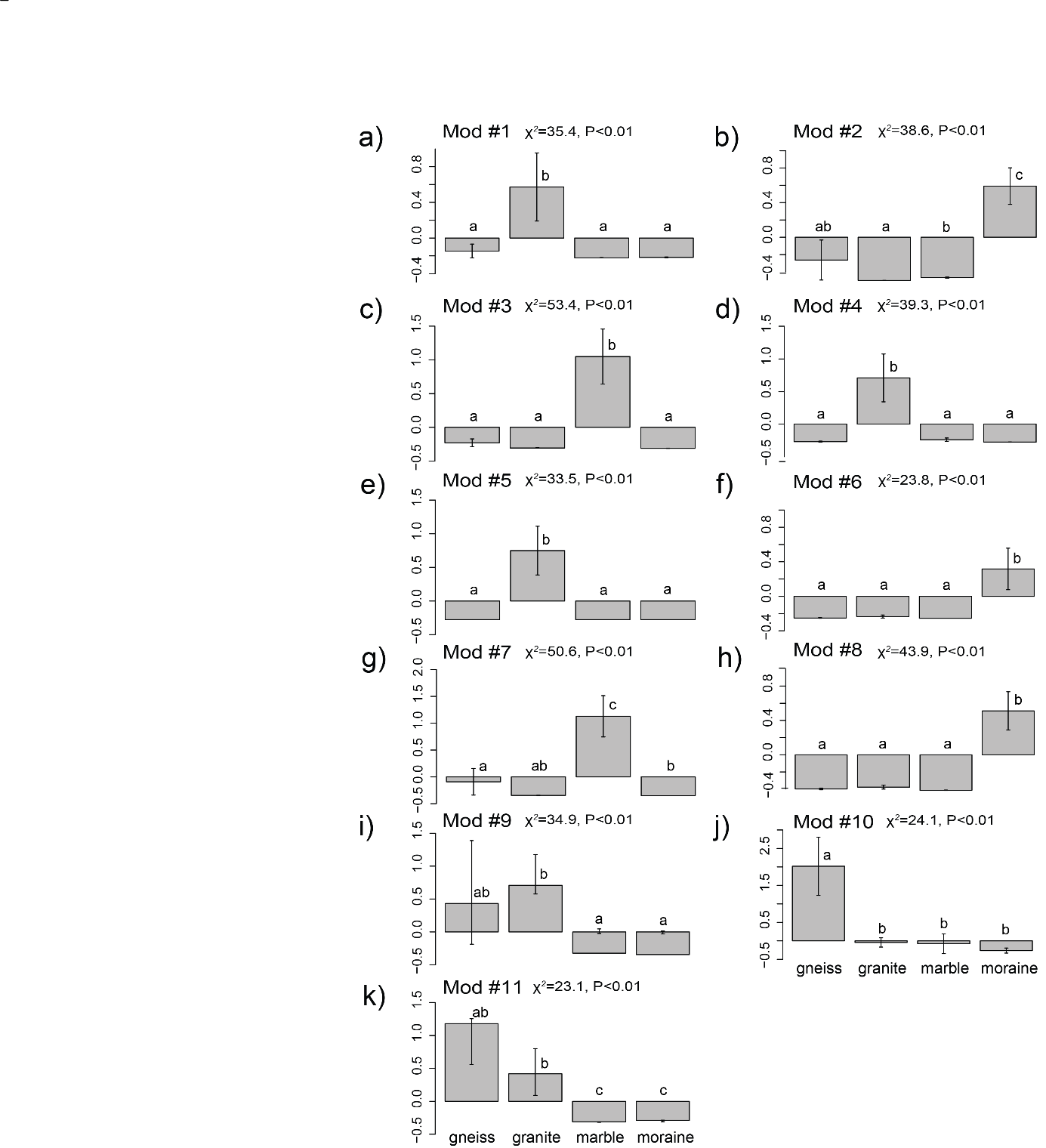
**

***Fig. S10 Mean values (±SE) for the relative abundance (Z-score) of modules #1–11 across four different substrate types.*** *Different letters in this panel indicate significant differences among substrate types. χ^2^ and P-value are from Kruskal-Wallis rank sum test.*

**I.II SUPPLEMENTARY TABLES**

***Table S1. Environmental data and number of reads for the 16S and 18S rRNA gene sequences***

| **Sample** | **Latitude** | **Longitude** | **Substrate** | **Substrate**  **detail** | **pH** | **EC**  **(µS cm^-1^)** | **TOC**  **(%)** | **N-NH_4_**  **(mg kg^-1^)** | **N-NO_3_**  **(mg kg^-1^)** | **Dry weight**  **(%)** | **P-PO_4_**  **(mg kg^-1^)** | **TN (%)** | **TP (%)** | **Elevation**  **(m a.s.l.)** | **# 16S reads** | **# 18S reads** |
| --- | --- | --- | --- | --- | --- | --- | --- | --- | --- | --- | --- | --- | --- | --- | --- | --- |
| AU_2018_11 | -71.661156 | 25.150839 | moraine | (Mo)_Amp | 7.77 | 164.6 | 0.61 | 3.034 | 23.99 | 99.81 | 337.269 | 0.12 | 0.33 | 930 | 79153 | 48216 |
| AU_2018_4 | -71.67534 | 25.136383 | moraine | (Mo)_Amp | 8.34 | 112.7 | 0.92 | 1.143 | 0.213 | 99.56 | 18.631 | 0.01 | 0.16 | 996 | 32443 | NA |
| AU_2018_8 | -71.67574 | 25.139032 | moraine | (Mo)_Amp | 8.2 | 165.6 | 0.81 | 0.536 | 80.518 | 99.38 | 15.965 | 0.02 | 0.12 | 994 | NA | 19445 |
| AU_2018_7 | -71.67593 | 25.136465 | moraine | (Mo)_Amp | 7.01 | 1112 | 0.68 | 0.742 | 0.421 | 99.65 | 25.596 | 0.01 | 0.21 | 995 | 118695 | 13805 |
| DV_2018_1 | -72.115875 | 23.157526 | moraine | (Mo)_Sch_D_Gbh_To | 7.96 | 2110 | 0.76 | 0.591 | 0.607 | 99.65 | 21.138 | 0.03 | 0.08 | 1714 | 28411 | 85993 |
| DV_2018_2 | -72.11599 | 23.157045 | moraine | (Mo)_Sch_D_Gbh_To | 6.36 | 119.5 | 0.46 | 1.645 | 0.133 | 99.55 | 16.177 | 0.02 | 0.08 | 1712 | 98841 | 1375 |
| DV_2018_3 | -72.11476 | 23.163443 | moraine | (Mo)_Sch_D_Gbh_To | 6.33 | 260 | 0.74 | 1.31 | 0.077 | 99.51 | 28.958 | 0.02 | 0.07 | 1694 | 134443 | NA |
| DV_2018_4 | -72.11468 | 23.163328 | moraine | (Mo)_Sch_D_Gbh_To | 6.44 | 78.9 | 0.49 | 1.085 | 0.021 | 99.64 | 24.585 | 0.03 | 0.07 | 1694 | 53267 | NA |
| DV_OTC12_2018_A | -72.11223 | 23.184898 | moraine | (Mo)_Sch_D_Gbh_To | 6.14 | 47.6 | 0.49 | 0.637 | 0.125 | 99.92 | 44.325 | 0.01 | 0.07 | 1666 | 45965 | 9917 |
| DV_OTC12_2018_B | -72.11223 | 23.184898 | moraine | (Mo)_Sch_D_Gbh_To | 6.29 | 140 | 0.3 | 0.922 | 0.135 | 99.87 | 40.959 | 0.01 | 0.09 | 1666 | 50727 | 19343 |
| DV_OTC12_2018_C | -72.11223 | 23.184898 | moraine | (Mo)_Sch_D_Gbh_To | 7.31 | 1175 | 0.45 | 1.245 | 0.989 | 99.73 | 42.879 | 0.04 | 0.09 | 1666 | 63362 | 60322 |
| DV_OTC13_2018_A | -72.11219 | 23.184841 | moraine | (Mo)_Sch_D_Gbh_To | 6.1 | 41.5 | 0.39 | 0.855 | 0.091 | 99.91 | 52.72 | 0.02 | 0.13 | 1667 | 54683 | 127788 |
| DV_OTC13_2018_B | -72.11219 | 23.184841 | moraine | (Mo)_Sch_D_Gbh_To | 6.78 | 57.2 | 0.7 | 1.342 | 0.202 | 99.88 | 43.947 | 0.02 | 0.1 | 1667 | 40174 | 128774 |
| DV_OTC13_2018_C | -72.11219 | 23.184841 | moraine | (Mo)_Sch_D_Gbh_To | 6.56 | 50.8 | 0.64 | 1.047 | 0.082 | 99.91 | 37.159 | 0.01 | 0.07 | 1667 | 32797 | 25292 |
| DV_OTC14_2018_B | -72.11219 | 23.184841 | moraine | (Mo)_Sch_D_Gbh_To | 6.5 | 264 | 0.4 | 0.927 | 0.646 | 99.89 | 41.475 | 0.06 | 0.15 | 1667 | 30670 | 117064 |
|  |  |  |  |  |  |  |  |  |  |  |  |  |  |  |  |  |
| **Sample** | **Latitude** | **Longitude** | **Substrate** | **Substrate**  **detail** | **pH** | **EC**  **(µS cm^-1^)** | **TOC**  **(%)** | **N-NH_4_**  **(mg kg^-1^)** | **N-NO_3_**  **(mg kg^-1^)** | **Dry weight**  **(%)** | **P-PO_4_**  **(mg kg^-1^)** | **TN (%)** | **TP (%)** | **Elevation**  **(m a.s.l.)** | **# 16S reads** | **#18S reads** |
| DV_OTC14_2018_C | -72.11219 | 23.184841 | moraine | (Mo)_Sch_D_Gbh_To | 6.46 | 46.9 | 0.3 | 1.275 | 0.211 | 99.92 | 43.192 | 0.05 | 0.15 | 1667 | 44296 | 101652 |
| DV_SF_2018_1_A | -72.11258 | 23.18555 | moraine | (Mo)_Sch_D_Gbh_To | 6.06 | 382 | 0.6 | 0.986 | 0.272 | 99.84 | 46.216 | 0.03 | 0.12 | 1667 | 44638 | NA |
| DV_SF_2018_1_B | -72.11258 | 23.18555 | moraine | (Mo)_Sch_D_Gbh_To | 6.45 | 59.2 | 0.34 | 0.802 | 0.143 | 99.74 | 37.011 | 0.02 | 0.09 | 1667 | 73228 | 45577 |
| DV_SF_2018_1_C | -72.11258 | 23.18555 | moraine | (Mo)_Sch_D_Gbh_To | 6.4 | 52.7 | 0.57 | 1.101 | 0.026 | 99.82 | 37.628 | 0.03 | 0.07 | 1667 | 72354 | 54217 |
| DV_SF_2018_2_A | -72.11258 | 23.18555 | moraine | (Mo)_Sch_D_Gbh_To | 6.22 | 34.7 | 0.38 | 0.94 | 0.102 | 99.87 | 47.913 | 0.02 | 0.11 | 1667 | 162952 | NA |
| DV_SF_2018_2_B | -72.11258 | 23.18555 | moraine | (Mo)_Sch_D_Gbh_To | 6.34 | 30.8 | 0.38 | 1.011 | 0.177 | 99.9 | 53.034 | 0.02 | 0.13 | 1667 | 26318 | 15084 |
| DV_SF_2018_2_C | -72.11258 | 23.18555 | moraine | (Mo)_Sch_D_Gbh_To | 5.98 | 40.8 | 0.52 | 1.041 | 0.918 | 99.86 | 57.324 | 0.03 | 0.13 | 1667 | 66432 | NA |
| DV_SF_2018_3_A | -72.11258 | 23.18555 | moraine | (Mo)_Sch_D_Gbh_To | 6.43 | 95 | 0.47 | 0.836 | 0.088 | 99.89 | 46.376 | 0.03 | 0.12 | 1667 | 33494 | NA |
| DV_SF_2018_3_B | -72.11258 | 23.18555 | moraine | (Mo)_Sch_D_Gbh_To | 5.94 | 328 | 0.19 | 0.946 | 0.157 | 99.88 | 50.124 | 0.03 | 0.13 | 1667 | 31021 | NA |
| DV_SF_2018_3_C | -72.11258 | 23.18555 | moraine | (Mo)_Sch_D_Gbh_To | 6.49 | 62.7 | 0.49 | 1.217 | 0.046 | 99.63 | 37.329 | 0.02 | 0.08 | 1667 | 154035 | 49349 |
| DV_SF_2018_CON_1_A | -72.11262 | 23.185282 | moraine | (Mo)_Sch_D_Gbh_To | 6.26 | 22.6 | 0.6 | 1.192 | 0.169 | 99.47 | 54.103 | 0.02 | 0.13 | 1668 | 33026 | 47456 |
| DV_SF_2018_CON_1_B | -72.11262 | 23.185282 | moraine | (Mo)_Sch_D_Gbh_To | 6.09 | 341 | 0.34 | 0.657 | 0.097 | 99.42 | 39.202 | 0.02 | 0.11 | 1668 | NA | 15693 |
| DV_SF_2018_CON_1_C | -72.11262 | 23.185282 | moraine | (Mo)_Sch_D_Gbh_To | 6.24 | 33.7 | 0.38 | 0.377 | 0.092 | 99.34 | 40.899 | 0.01 | 0.1 | 1668 | 36168 | 8002 |
| DV_SF_2018_CON_2_A | -72.11278 | 23.185186 | moraine | (Mo)_Sch_D_Gbh_To | 5.75 | 125.6 | 0.85 | 0.259 | 0.031 | 99.84 | 61.912 | 0.03 | 0.12 | 1668 | 49707 | 28584 |
| DV_SF_2018_CON_2_B | -72.11278 | 23.185186 | moraine | (Mo)_Sch_D_Gbh_To | 5.64 | 16.6 | 0.36 | 0.851 | 0.041 | 99.12 | 52.525 | 0.02 | 0.12 | 1668 | 104192 | 19851 |
| DV_SF_2018_CON_2_C | -72.11278 | 23.185186 | moraine | (Mo)_Sch_D_Gbh_To | 5.55 | 38.9 | 0.35 | 0.631 | 0.031 | 98.91 | 62.933 | 0.03 | 0.12 | 1668 | 66614 | 68553 |
| **Sample** | **Latitude** | **Longitude** | **Substrate** | **Substrate**  **detail** | **pH** | **EC**  **(µS cm^-1^)** | **TOC**  **(%)** | **N-NH_4_**  **(mg kg^-1^)** | **N-NO_3_**  **(mg kg^-1^)** | **Dry weight**  **(%)** | **P-PO_4_**  **(mg kg^-1^)** | **TN (%)** | **TP (%)** | **Elevation**  **(m a.s.l.)** | **# 16S reads** | **# 18S reads** |
| DV_SF_2018_CON_3_A | -72.11271 | 23.184937 | moraine | (Mo)_Sch_D_Gbh_To | 6.14 | 37.4 | 0.38 | 0.477 | 0.634 | 99.72 | 53.264 | 0.02 | 0.09 | 1668 | 16321 | 43271 |
| DV_SF_2018_CON_3_B | -72.11271 | 23.184937 | moraine | (Mo)_Sch_D_Gbh_To | 5.81 | 22.3 | 0.3 | 0.504 | 0.232 | 99.19 | 63.504 | 0.02 | 0.12 | 1668 | 29065 | 40965 |
| DV_SF_2018_CON_3_C | -72.11271 | 23.184937 | moraine | (Mo)_Sch_D_Gbh_To | 5.88 | 16.9 | 0.43 | 0.733 | 0.056 | 99.19 | 42.924 | 0.02 | 0.12 | 1668 | 29400 | NA |
| DV_SF2_2018_1_A | -72.11214 | 23.185244 | moraine | (Mo)_Sch_D_Gbh_To | 6.44 | 26.4 | 0.44 | 1.234 | 0.066 | 99.34 | 44.511 | 0.01 | 0.05 | 1666 | 77807 | 13801 |
| DV_SF2_2018_1_B | -72.11214 | 23.185244 | moraine | (Mo)_Sch_D_Gbh_To | 6.95 | 39.2 | 0.44 | 1.167 | 0.112 | 99.25 | 51.044 | 0.02 | 0.08 | 1666 | 115279 | 81302 |
| DV_SF2_2018_1_C | -72.11214 | 23.185244 | moraine | (Mo)_Sch_D_Gbh_To | 7.8 | 74.9 | 0.57 | 2.735 | 0.619 | 99.89 | 55.622 | 0.04 | 0.12 | 1666 | 105677 | 27177 |
| DV_SF2_2018_2_A | -72.112114 | 23.185379 | moraine | (Mo)_Sch_D_Gbh_To | 6.41 | 29 | 0.73 | 1.082 | 0.081 | 99.52 | 48.976 | 0.02 | 0.11 | 1666 | 50966 | 106464 |
| DV_SF2_2018_2_B | -72.112114 | 23.185379 | moraine | (Mo)_Sch_D_Gbh_To | 6.51 | 32.8 | 0.58 | 0.636 | 0.076 | 99.87 | 50.217 | 0.03 | 0.11 | 1666 | 59901 | 17667 |
| DV_SF2_2018_2_C | -72.112114 | 23.185379 | moraine | (Mo)_Sch_D_Gbh_To | 6.59 | 100.4 | 0.53 | 0.831 | 0.549 | 99.88 | 42.732 | 0.04 | 0.1 | 1666 | 72295 | NA |
| DV_SF2_2018_3_A | -72.112114 | 23.185379 | moraine | (Mo)_Sch_D_Gbh_To | 5.91 | 19.3 | 0.48 | 0.663 | 0.051 | 99.88 | 62.873 | 0.02 | 0.13 | 1666 | 53201 | 44627 |
| DV_SF2_2018_3_B | -72.112114 | 23.185379 | moraine | (Mo)_Sch_D_Gbh_To | 6.29 | 108.7 | 0.43 | 0.482 | 0.188 | 99.81 | 51.853 | 0.02 | 0.1 | 1666 | 151156 | 119697 |
| DV_SF2_2018_3_C | -72.112114 | 23.185379 | moraine | (Mo)_Sch_D_Gbh_To | 5.98 | 18.2 | 0.46 | 0.889 | 0.076 | 99.75 | 53.056 | 0.01 | 0.12 | 1666 | 126795 | 39826 |
| DV_SF2_2018_CON_1_A | -72.11206 | 23.184668 | moraine | (Mo)_Sch_D_Gbh_To | 6.23 | 21.1 | 0.49 | 0.327 | 0.101 | 99.65 | 55.756 | 0.01 | 0.1 | 1667 | 20881 | 42126 |
| DV_SF2_2018_CON_1_B | -72.11206 | 23.184668 | moraine | (Mo)_Sch_D_Gbh_To | 6.36 | 64.9 | 0.63 | 0.518 | 0.086 | 99.72 | 48.878 | 0.01 | 0.09 | 1667 | 56442 | 26676 |
| DV_SF2_2018_CON_1_C | -72.11206 | 23.184668 | moraine | (Mo)_Sch_D_Gbh_To | 6.85 | 185.5 | 0.67 | 0.558 | 0.091 | 99.68 | 56.133 | 0.02 | 0.12 | 1667 | 43438 | 69557 |
| PB_N_OTC15_16_17_2019_CON_A | -71.84423 | 22.831173 | marble | (M) | 9.09 | 208.1 | 0.48 | 0.926 | 0.27 | 99.95 | 6.899 | 0.04 | 0.03 | 1310 | 6074 | NA |
| **Sample** | **Latitude** | **Longitude** | **Substrate** | **Substrate**  **detail** | **pH** | **EC**  **(µS cm^-1^)** | **TOC**  **(%)** | **N-NH_4_**  **(mg kg^-1^)** | **N-NO_3_**  **(mg kg^-1^)** | **Dry weight**  **(%)** | **P-PO_4_**  **(mg kg^-1^)** | **TN (%)** | **TP (%)** | **Elevation**  **(m a.s.l.)** | **# 16S reads** | **# 18S reads** |
| PB_N_OTC15_2019_A | -71.84423 | 22.831173 | marble | (M) | 9.31 | 68.2 | 0.34 | 0.816 | 0.06 | 99.92 | 6.636 | 0.13 | 0.08 | 1310 | 42613 | 35953 |
| PB_N_OTC15_2019_B | -71.84423 | 22.831173 | marble | (M) | 9.21 | 58.2 | 0.5 | 0.292 | 0.05 | 99.78 | 6.686 | 0.06 | 0.07 | 1310 | 10504 | 98432 |
| PB_N_OTC15_2019_C | -71.84423 | 22.831173 | marble | (M) | 9.34 | 60.2 | 0.46 | 0.096 | 0.031 | 99.85 | 6.537 | 0.13 | 0.08 | 1310 | 23842 | NA |
| PB_N_OTC16_2019_A | -71.84423 | 22.831173 | marble | (M) | 9.22 | 62.9 | 0.69 | 0.786 | 0.121 | 99.81 | 6.571 | 0.07 | 0.05 | 1310 | 43381 | 63102 |
| PB_N_OTC16_2019_B | -71.84423 | 22.831173 | marble | (M) | 9.13 | 65.4 | 0.47 | 0.105 | 0.26 | 99.95 | 6.666 | 0.05 | 0.05 | 1310 | 12665 | 62095 |
| PB_N_OTC16_2019_C | -71.84423 | 22.831173 | marble | (M) | 9.08 | 90.7 | 0.6 | 0.286 | 0.827 | 99.89 | 6.445 | 0.06 | 0.06 | 1310 | 8694 | 83367 |
| PB_N_OTC17_2019_A | -71.84423 | 22.831173 | marble | (M) | 8.73 | 72.5 | 0.59 | 1.081 | 0.025 | 99.74 | 7.534 | 0.08 | 0.13 | 1310 | 6270 | 85397 |
| PB_N_OTC17_2019_B | -71.84423 | 22.831173 | marble | (M) | 8.93 | 57.6 | 0.38 | 0.997 | 0.022 | 99.91 | 6.749 | 0.06 | 0.04 | 1310 | 18596 | 13355 |
| PB_N_OTC17_2019_C | -71.84423 | 22.831173 | marble | (M) | 8.88 | 66.4 | 0.69 | 1.354 | 0.015 | 99.74 | 6.906 | 0.08 | 0.07 | 1310 | 427 | NA |
| PA4_2018_1 | -72.00135 | 22.999208 | granite | (Gr) | 6.12 | 22.9 | 0.76 | 2.753 | 0.088 | 99.75 | 48.568 | 0.06 | 0.12 | 1409 | 37553 | 64886 |
| PA4_2018_2 | -72.00131 | 22.999477 | granite | (Gr) | 6.06 | 19.4 | 0.88 | 0.989 | 0.089 | 99.82 | 55.398 | 0.08 | 0.09 | 1410 | 52255 | 65644 |
| PA4_2018_3 | -72.00144 | 22.999647 | granite | (Gr) | 6.41 | 22.4 | 1.46 | 3.359 | 0.389 | 99.45 | 57.074 | 0.09 | 0.13 | 1410 | 96637 | 91044 |
| PA4_2018_4 | -72.00144 | 22.999647 | granite | (Gr) | 6.58 | 20.7 | 1.05 | 2.004 | 0.223 | 99.36 | 61.305 | 0.03 | 0.05 | 1410 | 37361 | 39331 |
| PA4_2018_5 | -72.00149 | 23.000029 | granite | (Gr) | 6.21 | 21.1 | 0.43 | 1.379 | 0.111 | 99.45 | 67.494 | 0.14 | 0.1 | 1411 | 47827 | 45814 |
| PA4_2018_6 | -72.00143 | 23.000105 | granite | (Gr) | 6.22 | 15.4 | 1.13 | 1.464 | 0.116 | 99.25 | 40.294 | 0.03 | 0.06 | 1412 | 61407 | 90664 |
| PA4_2018_7 | -72.00139 | 23.000048 | granite | (Gr) | 6.45 | 47.4 | 1.54 | 8.585 | 0.108 | 99.74 | 55.434 | 0.1 | 0.05 | 1412 | 37221 | 96722 |
| PA6_2019_1 | -72.010506 | 22.989674 | granite | (Gr) | 7.31 | 62.1 | 0.78 | 2.017 | 0.898 | 99.41 | 86.474 | 0.06 | 0.09 | 1412 | 33007 | NA |
| PA6_2019_2 | -72.01036 | 22.991528 | granite | (Gr) | 7.04 | 193.4 | 1.47 | 1.859 | 0.289 | 98.25 | 73.589 | 0.07 | 0.07 | 1416 | 5397 | 112914 |
| PA6_2019_4 | -72.010056 | 22.993479 | granite | (Gr) | 6.97 | 24.8 | 0.31 | 7.283 | 0.312 | 99.75 | 114.824 | 0.02 | 0.09 | 1416 | 91806 | 97871 |
| PA6_2019_5 | -72.01002 | 22.993422 | granite | (Gr) | 7.57 | 65.6 | 0.32 | 8.557 | 1.053 | 99.85 | 95.352 | 0.03 | 0.06 | 1416 | 46842 | 221282 |
| PA6_2019_6 | -72.0101 | 22.993536 | granite | (Gr) | 6.52 | 108.8 | 3.4 | 24.04 | 5.464 | 99.35 | 119.427 | 0.46 | 0.02 | 1417 | 8795 | 24800 |
| **Sample** | **Latitude** | **Longitude** | **Substrate** | **Substrate**  **detail** | **pH** | **EC**  **(µS cm^-1^)** | **TOC**  **(%)** | **N-NH_4_**  **(mg kg^-1^)** | **N-NO_3_**  **(mg kg^-1^)** | **Dry weight**  **(%)** | **P-PO_4_**  **(mg kg^-1^)** | **TN (%)** | **TP (%)** | **Elevation**  **(m a.s.l.)** | **# 16S reads** | **# 18S reads** |
| PB_N_2018_1 | -71.84361 | 22.838146 | gneiss | (Gbg) | 5.88 | 414.2 | 9.23 | 20.526 | 0.04 | 98.25 | 38.751 | 0.23 | 0.4 | 1256 | 86362 | 270390 |
| PB_N_2018_10 | -71.84405 | 22.834051 | marble | (Gbg) | 9.09 | 149.7 | 1.48 | 0.89 | 0.506 | 99.74 | 7.968 | 0.27 | 0.21 | 1287 | 134 | 71300 |
| PB_N_2018_11 | -71.84405 | 22.834051 | marble | (Gbg) | 8.98 | 56.5 | 0.79 | 0.437 | 0.036 | 99.68 | 8.81 | 0.15 | 0.12 | 1287 | 106411 | 74000 |
| PB_N_2018_12 | -71.84414 | 22.833708 | gneiss | (Gbg) | 8.51 | 62.2 | 0.45 | 0.404 | 0.828 | 99.75 | 9.592 | 0.05 | 0.09 | 1290 | 40 | 34500 |
| PB_N_2018_3 | -71.84353 | 22.838034 | gneiss | (Gbg) | 5.01 | 163.1 | 10.95 | 46.657 | 0.136 | 98.21 | 48.899 | 0.28 | 0.17 | 1255 | 58605 | 15081 |
| PB_N_2018_6 | -71.84395 | 22.835169 | gneiss | (Gbg) | 5.63 | 91.7 | 4.6 | 12.926 | 0.036 | 99.15 | 34.056 | 0.2 | 0.41 | 1279 | 48899 | 74851 |
| PB_N_2018_7 | -71.843895 | 22.835245 | gneiss | (Gbg) | 5.82 | 49.2 | 2.52 | 13.117 | 0.131 | 99.22 | 18.639 | 0.16 | 0.16 | 1277 | 52543 | 39805 |
| PB_N_2018_8 | -71.84399 | 22.834904 | gneiss | (Gbg) | 8.38 | 89.4 | 0.68 | 3.906 | 0.964 | 99.02 | 23.818 | 0.07 | 0.81 | 1282 | 139319 | 47148 |
| PB_N_2018_9 | -71.84405 | 22.834051 | marble | (M) | 7.4 | 336 | 5.62 | 4.553 | 0.351 | 99.45 | 18.515 | 0.36 | 0.23 | 1287 | 31911 | 82615 |
| PB_N_OTC9_10_CON_A | -71.84405 | 22.834051 | marble | (M) | 9.42 | 117.9 | 1.19 | 0.321 | 0.035 | 99.91 | 6.918 | 0.24 | 0.12 | 1287 | 31565 | 47675 |
| PB_N_OTC9_10_CON_B | -71.84405 | 22.834051 | marble | (M) | 9.17 | 73.6 | 0.58 | 0.59 | 0.06 | 99.95 | 7.173 | 0.27 | 0.16 | 1287 | 32512 | 119600 |
| PB_N_OTC9_10_CON_C | -71.84405 | 22.834051 | marble | (M) | 9.45 | 117.3 | 1.14 | 0.282 | 0.05 | 99.78 | 7.104 | 0.21 | 0.1 | 1287 | 50091 | 39996 |
| PB_N_OTC9_A_18 | -71.84405 | 22.834051 | marble | (M) | 9.46 | 160.4 | 1.3 | 0.761 | 0.062 | 99.91 | 8.042 | 0.23 | 0.15 | 1287 | 25218 | 6032 |
| PB_N_OTC9_B | -71.84405 | 22.834051 | marble | (M) | 9.49 | 185.5 | 1.49 | 0.392 | 0.151 | 99.78 | 8.109 | 0.17 | 0.12 | 1287 | 45852 | 48553 |
| PB_N_OTC9_C_18 | -71.84405 | 22.834051 | marble | (M) | 9.55 | 142.6 | 1.56 | 0.748 | 0.045 | 99.82 | 9.184 | 0.26 | 0.12 | 1287 | 15260 | 81208 |
| PB_S_2018_1 | -71.8797 | 22.746996 | gneiss | (Gbh) | 6.45 | 17.6 | 1.41 | 1.648 | 0.088 | 99.27 | 17.073 | 0.09 | 0.16 | 1349 | 71864 | 78606 |
| PB_S_2018_2 | -71.87994 | 22.746687 | marble | (M) | 9.01 | 63.7 | 0.53 | 0.299 | 0.173 | 99.35 | 14.094 | 0.04 | 0.1 | 1354 | 23529 | 108313 |
| PB_S_2018_3 | -71.88009 | 22.74659 | marble | (M) | 8.94 | 50.7 | 0.78 | 0.333 | 0.09 | 99.41 | 14.28 | 0.04 | 0.04 | 1357 | 43623 | 294308 |
| PT_2019_1_A | -72.007385 | 22.832005 | granite | (Gr) | 6.11 | 28.8 | 0.15 | 2.204 | 0.387 | 99.45 | 52.049 | 0.05 | 0.11 | 1469 | NA | 154275 |
| PT_2019_2 | -72.007385 | 22.832005 | granite | (Gr) | 6.13 | 70.4 | 1.28 | 2.444 | 3.837 | 99.57 | 60.359 | 0.11 | 0.14 | 1469 | 845 | 404670 |
| PT_2019_3 | -72.007416 | 22.831738 | granite | (Gr) | 7.11 | 31.8 | 0.45 | 3.258 | 0.428 | 99.47 | 42.777 | 0.04 | 0.06 | 1472 | 5459 | 84779 |
| PT_2019_5 | -72.00757 | 22.833204 | granite | (Gr) | 7.01 | 14.9 | 0.25 | 0.721 | 0.28 | 99.9 | 36.502 | 0.03 | 0.07 | 1460 | 16428 | 13779 |
| UT_2018_1 | -71.9456 | 23.345005 | granite | (Gr) | 5.89 | 21.1 | 2.46 | 7.048 | 0.303 | 99.55 | 64.229 | 0.15 | 0.11 | 1362 | 27478 | 30572 |
| **Sample** | **Latitude** | **Longitude** | **Substrate** | **Substrate**  **detail** | **pH** | **EC**  **(µS cm^-1^)** | **TOC**  **(%)** | **N-NH_4_**  **(mg kg^-1^)** | **N-NO_3_**  **(mg kg^-1^)** | **Dry weight**  **(%)** | **P-PO_4_**  **(mg kg^-1^)** | **TN (%)** | **TP**  **(%)** | **Elevation**  **(m a.s.l.)** | **# 16S reads** | **# 18S reads** |
| UT_2018_2 | -71.94543 | 23.345041 | granite | (Gr) | 6.28 | 20.9 | 1.83 | 2.835 | 0.077 | 99.28 | 26.373 | 0.16 | 0.15 | 1358 | 102793 | NA |
| UT_2018_3 | -71.94559 | 23.345139 | granite | (Gr) | 5.24 | 30.1 | 2.15 | 5.929 | 0.084 | 99.36 | 151.121 | 0.22 | 0.12 | 1361 | 80256 | 107111 |
| UT_2018_4 | -71.94573 | 23.345177 | granite | (Gr) | 6.1 | 15.6 | 1.24 | 1.483 | 0.086 | 99.62 | 34.375 | 0.35 | 0.09 | 1364 | 49357 | 91872 |
| UT_2018_6 | -71.94626 | 23.3456 | granite | (Gr) | 5.95 | 30.3 | 1.1 | 1.607 | 0.076 | 99.75 | 39.742 | 0.25 | 0.12 | 1372 | 32239 | 43205 |
| UT_2018_7 | -71.94624 | 23.345734 | granite | (Gr) | 5.87 | 14.2 | 0.59 | 1.151 | 0.146 | 99.81 | 40.212 | 0.07 | 0.04 | 1370 | 95682 | 59959 |
| UT_OTC11_2018_A | -71.945465 | 23.344967 | granite | (Gr) | 5.92 | 58.1 | 2.49 | 2.059 | 0.091 | 99.72 | 23.943 | 0.35 | 0.17 | 1359 | 107 | 36767 |
| UT_OTC11_2018_CON_B | -71.945465 | 23.344967 | granite | (Gr) | 6.73 | 36.6 | 0.89 | 1.334 | 0.115 | 99.89 | 43.154 | 0.09 | 0.09 | 1359 | 29017 | 69947 |
| UT_OTC2_2018_CON_1_A | -71.946075 | 23.345505 | granite | (Gr) | 5.83 | 329.1 | 2.67 | 30.354 | 0.497 | 99.25 | 96.667 | 0.34 | 0.24 | 1369 | 65588 | 42987 |
| UT_OTC2_2018_CON_1_B | -71.946075 | 23.345505 | granite | (Gr) | 5.9 | 89.8 | 1.31 | 5.449 | 0.082 | 99.12 | 66.263 | 0.35 | 0.26 | 1369 | 45595 | 42684 |
| UT_OTC2_2018_CON_1_C | -71.946075 | 23.345505 | granite | (Gr) | 5.97 | 273.1 | 2.13 | 22.79 | 1.773 | 99.72 | 50.768 | 0.43 | 0.2 | 1369 | 69907 | 55313 |
| YO_2019_3 | -72.08115 | 23.793186 | moraine | (Mo)_Sch_To | 8.81 | 90.5 | 0.54 | 0.256 | 0.236 | 99.81 | 22.712 | 0.04 | 0.09 | 1361 | 10252 | 16922 |
| YO_2018_5 | -72.081375 | 23.793673 | moraine | (Mo)_Sch_To | 8.45 | 110.8 | 1.19 | 0.627 | 0.197 | 99.74 | 15.318 | 0.05 | 0.1 | 1360 | 43198 | 88956 |
| YO_2019_6 | -72.08344 | 23.790731 | moraine | (Mo)_Sch_To | 8.96 | 93.7 | 0.57 | 0.286 | 2.38 | 99.65 | 27.897 | 0.02 | 0.08 | 1357 | 15497 | 120712 |
| YO_2018_6 | -72.08111 | 23.793453 | moraine | (Mo)_Sch_To | 8.53 | 60.2 | 1.38 | 0.407 | 0.296 | 99.82 | 16.151 | 0.03 | 0.04 | 1360 | 76449 | 54595 |

AU: Austkampane; DV: Dry Valley; PB_N: Perlebandet North; PB_S: Perlebandet South; PA4: Pingvinane 4^th^ nunatak; PA6: Pingvinane 6^th^ nunatak; PT: Petrellnuten; UT: Utsteinen; YO: Yûboku-dani Valley. (Mo)_Amp: Moraine with orthopyroxene amphibolite; (Mo)_Sch_D_Gbh_To: Moraine with amphibole schist, diorite and quartz, biotite-hornblende gneiss and metatonalite; (M): Marble and skarns; (Gbg): Sillimanite-garnet-biotite gneiss, partly containing cordierite; (Gr): Granite and pegmatite; (Gbh): Biotite-hornblende gneiss and hornblende gneiss containing garnet in places; (Mo)_Sch_To: Moraine with amphibole schist and metatonalite. EC: electric conductivity; TOC: total carbon; TN: total nitrogen; TP: total phosphorus.

***Table S2. Mock community for the 18S rRNA amplicon sequencing***

| Species ID | Species name | mock  (% cell count) |
| --- | --- | --- |
| M01_1352 | *Ulnaria ulna* | 4.28 |
| M02_1343 | *Ulnaria ulna* |  |
| M03_494 | *Ulnaria ulna* |  |
| M04_1180 | *Fragilaria crotonensis* | 5.38 |
| M05_1163 | *Fragilaria crotonensis* | 15.28 |
| M06_1164 | *Fragilaria crotonensis* |  |
| M07_512 | *Fragilaria nanana* | 3.06 |
| M08_1176 | *Asterionella formosa* | 13.08 |
| M09_679 | *Tabellaria flocculosa* | 7.58 |
| M10_499 | *Aulacoseira granulata* | 3.91 |
| M11_1170 | *Aulacoseira subarctica* | 35.09 |
| M12_1175 | *Nitzschia palea* | 7.58 |
| M13_1770 | *Staurodesmus sp.* | 0.12 |
| M14_1771 | *Chlorella vulgaris* | 0.86 |
| M15_1772 | *Peridinium sp.* | 0.12 |
| M16_1773 | *Cosmarium reniforme* | 0.24 |
| M17_1774 | *Desmodesmus sp.* | 1.22 |
| M18_1775 | *Scenedesmus sp.* | 0.12 |
| M19_1776 | *Mallomonas sp.* | 0.24 |
| M20_1777 | *Tetrahymena pyriformis* | 1.71 |
| M21_1778 | *Dinobryon sp.* | 0.12 |

***Table S3. Kruskal-Wallis rank sum test showing the effect of substrate type on environmental parameters.***

|  | df | χ2 | P |
| --- | --- | --- | --- |
| pH | 3 | 49.861 | <0.001 |
| TOC | 3 | 25.983 | <0.001 |
| N-NO_3_ | 3 | 9.9442 | <0.05 |
| N-NH_4_ | 3 | 51.778 | <0.001 |
| TN | 3 | 63.1 | <0.001 |
| P-PO_4_ | 3 | 59.322 | <0.001 |
| TP | 3 | 12.324 | <0.05 |
| DW | 3 | 23.604 | <0.001 |
| EC | 3 | 14.945 | <0.05 |

EC: Electric conductivity; TOC: Total carbon; TN: Total nitrogen; TP: Total phosphorus

***Table S4. Relative influence of environmental, spatial and substrate type variables on bacterial communities using a dbRDA and a forward, stepwise variable selection.***

| Environmental variables | Adj. R2 | F | df | P |
| --- | --- | --- | --- | --- |
| Elevation | 0.07 | 8.26 | 1 | 0.002 |
| pH | 0.12 | 6.24 | 1 | 0.002 |
| TN | 0.14 | 3.14 | 1 | 0.002 |
| Conductivity | 0.15 | 2.58 | 1 | 0.002 |
| TP | 0.17 | 2.21 | 1 | 0.002 |
| NH4 | 0.17 | 1.86 | 1 | 0.002 |
| NO3 | 0.18 | 1.51 | 1 | 0.014 |
| Dry weight | 0.18 | 1.51 | 1 | 0.01 |
| TOC | 0.19 | 1.58 | 1 | 0.01 |
| All | 0.19 | NA | NA | NA |
|  |  |  |  |  |
| PCNM | Adj. R2 | F | df | P |
| PCNM3 | 0.07 | 0.09 | 1 | 0.002 |
| PCNM4 | 0.09 | 0.10 | 1 | 0.002 |
| PCNM1 | 0.10 | 0.09 | 1 | 0.002 |
| PCNM2 | 0.10 | 0.10 | 1 | 0.002 |
| All | 0.11 | NA | NA | NA |
|  |  |  |  |  |
| Substrate type | Adj. R2 | F | df | P |
| moraine | 0.08 | 9.54 | 1 | 0.002 |
| marble | 0.13 | 6.52 | 1 | 0.002 |
| gneiss | 0.14 | 2.17 | 1 | 0.002 |
| All | 0.14 | NA | NA | NA |

***Table S5. Relative influence of environmental, spatial and substrate type variables on eukaryotic communities using a dbRDA and a forward, stepwise variable selection.***

| Environmental variables | Adj. R2 | F | df | P |
| --- | --- | --- | --- | --- |
| pH | 0.06 | 7.32 | 1 | 0.002 |
| Elevation | 0.13 | 7.94 | 1 | 0.002 |
| TN | 0.16 | 4.50 | 1 | 0.002 |
| Conductivity | 0.17 | 2.57 | 1 | 0.002 |
| TP | 0.18 | 2.02 | 1 | 0.004 |
| TOC | 0.19 | 2.00 | 1 | 0.002 |
| N_NO3 | 0.20 | 1.99 | 1 | 0.004 |
| N_NH4 | 0.21 | 1.52 | 1 | 0.028 |
| All | 0.21 | NA | NA | NA |
|  |  |  |  |  |
| PCNM | Adj. R2 | F | df | P |
| PCNM3 | 0.05 | 6.25 | 1.00 | 0.00 |
| PCNM4 | 0.08 | 3.51 | 1.00 | 0.00 |
| PCNM1 | 0.09 | 2.65 | 1.00 | 0.00 |
| All | 0.10 | 2.21 | 1.00 | 0.00 |
|  |  |  |  |  |
| Substrate type | Adj. R2 | F | df | P |
| moraine | 0.08 | 8.77 | 1.00 | 0.00 |
| marble | 0.14 | 8.41 | 1.00 | 0.00 |
| granite | 0.16 | 3.03 | 1.00 | 0.00 |
| All | 0.16 | NA | 1.00 | NA |

***Table S6. Taxonomic assignment at the bacterial phylum level of the ASVs with cumulative abundances calculated for each substrate type.***

| **Granite** | | | **Gneiss** | | |
| --- | --- | --- | --- | --- | --- |
| **Phylum** | **n. reads** | **% reads** | **Phylum** | **n. reads** | **% reads** |
| Cyanobacteriota | 238856 | 31.97% | Acidobacteriota | 92844 | 35.64% |
| Acidobacteriota | 204719 | 27.40% | Cyanobacteriota | 52823 | 20.28% |
| Actinobacteriota | 123943 | 16.59% | Actinobacteriota | 31466 | 12.08% |
| Bacteroidota | 62056 | 8.31% | Pseudomonadota | 28660 | 11.00% |
| Pseudomonadota | 32563 | 4.36% | Bacteroidota | 25496 | 9.79% |
| Abditibacteriota | 26897 | 3.60% | Chloroflexota | 11279 | 4.33% |
| Bacteria_unclassified | 24335 | 3.26% | Armatimonadota | 6520 | 2.50% |
| Chloroflexota | 11120 | 1.49% | Abditibacteriota | 4824 | 1.85% |
| Deinococcota | 6592 | 0.88% | Bacteria_unclassified | 2728 | 1.05% |
| Patescibacteria | 6093 | 0.82% | Planctomycetota | 1785 | 0.69% |
| Armatimonadota | 4114 | 0.55% | Deinococcota | 1164 | 0.45% |
| Planctomycetota | 2840 | 0.38% | Gemmatimonadota | 368 | 0.14% |
| Gemmatimonadota | 1584 | 0.21% | Patescibacteria | 203 | 0.08% |
| Myxococcota | 792 | 0.11% | Bdellovibrionota | 77 | 0.03% |
| unknown_unclassified | 475 | 0.06% | unknown_unclassified | 71 | 0.03% |
| Verrucomicrobiota | 54 | 0.01% | Myxococcota | 69 | 0.03% |
| Bdellovibrionota | 45 | 0.01% | Verrucomicrobiota | 53 | 0.02% |
| WPS-2 | 25 | 0.00% | WPS-2 | 51 | 0.02% |
| Sumerlaeota | 5 | 0.00% | Nitrospirota | 30 | 0.01% |
| Bacillota | 2 | 0.00% | SAR324_clade(Marine_group_B) | 8 | 0.00% |
|  |  |  | Bacillota | 4 | 0.00% |
|  |  |  | Elusimicrobiota | 3 | 0.00% |
|  |  |  | Sumerlaeota | 2 | 0.00% |
|  |  |  |  |  |  |
| **Marble** | | | **Moraine** | | |
| **Phylum** | **n. reads** | **% reads** | **Phylum** | **n. reads** | **% reads** |
| Cyanobacteriota | 115789 | 32.54% | Actinobacteriota | 920322 | 49.13% |
| Acidobacteriota | 103679 | 29.14% | Acidobacteriota | 576097 | 30.75% |
| Bacteroidota | 55915 | 15.71% | Chloroflexota | 100324 | 5.36% |
| Actinobacteriota | 31358 | 8.81% | Bacteroidota | 64721 | 3.45% |
| Pseudomonadota | 14742 | 4.14% | Cyanobacteriota | 54147 | 2.89% |
| Deinococcota | 10948 | 3.08% | Patescibacteria | 48464 | 2.59% |
| Abditibacteriota | 8186 | 2.30% | Pseudomonadota | 25947 | 1.39% |
| Chloroflexota | 6185 | 1.74% | Abditibacteriota | 25012 | 1.34% |
| Bacteria_unclassified | 5899 | 1.66% | Bacteria_unclassified | 21774 | 1.16% |
| Planctomycetota | 1284 | 0.36% | Deinococcota | 11303 | 0.60% |
| Patescibacteria | 840 | 0.24% | Gemmatimonadota | 9486 | 0.51% |
| Armatimonadota | 589 | 0.17% | Armatimonadota | 8693 | 0.46% |
| Myxococcota | 140 | 0.04% | Planctomycetota | 4150 | 0.22% |
|  |  |  |  |  |  |
| **Marble** | | | **Moraine** | | |
| **Phylum** | **n. reads** | **% reads** | **Phylum** | **n. reads** | **% reads** |
| unknown_unclassified | 117 | 0.03% | Bacillota | 1450 | 0.08% |
| Gemmatimonadota | 67 | 0.02% | Verrucomicrobiota | 495 | 0.03% |
| Bdellovibrionota | 66 | 0.02% | unknown_unclassified | 393 | 0.02% |
| SAR324_clade(Marine_group_B) | 13 | 0.00% | Nitrospirota | 212 | 0.01% |
| Verrucomicrobiota | 11 | 0.00% | Myxococcota | 153 | 0.01% |
| Bacillota | 6 | 0.00% | Bdellovibrionota | 121 | 0.01% |
| WPS-2 | 4 | 0.00% | Sumerlaeota | 44 | 0.00% |
|  |  | 0.00% | SAR324_clade(Marine_group_B) | 35 | 0.00% |
|  |  | 0.00% | WPS-2 | 35 | 0.00% |
|  |  | 0.00% | Desulfobacterota | 13 | 0.00% |
|  |  | 0.00% | Dependentiae | 8 | 0.00% |

***Table S7****. Taxonomic assignment at the bacterial family-level of the ASVs with cumulative abundance calculated for each substrate type.*

| **Granite** | | | **Gneiss** | | |
| --- | --- | --- | --- | --- | --- |
| **Family** | **n. reads** | **% reads** | **Family** | **n. reads** | **% reads** |
| Blastocatellaceae | 202488 | 27.10% | Blastocatellaceae | 80105 | 30.75% |
| Nostocaceae | 150339 | 20.12% | Chroococcidiopsaceae | 45003 | 17.27% |
| Chroococcidiopsaceae | 32603 | 4.36% | Acetobacteraceae | 19566 | 7.51% |
| Solirubrobacteraceae | 31525 | 4.22% | Pseudonocardiaceae | 16532 | 6.35% |
| Phormidiaceae | 31150 | 4.17% | Acidobacteriaceae_(Subgroup_1) | 9930 | 3.81% |
| Chitinophagaceae | 27480 | 3.68% | Chitinophagaceae | 9796 | 3.76% |
| Abditibacteriaceae | 26897 | 3.60% | Ktedonobacteraceae | 7926 | 3.04% |
| Bacteria_unclassified | 24335 | 3.26% | Armatimonadales_fa | 6464 | 2.48% |
| Pseudonocardiaceae | 23244 | 3.11% | Hymenobacteraceae | 6258 | 2.40% |
| Acetobacteraceae | 17819 | 2.39% | Spirosomaceae | 5992 | 2.30% |
| Nocardioidaceae | 14312 | 1.92% | Abditibacteriaceae | 4824 | 1.85% |
| Hymenobacteraceae | 12109 | 1.62% | Phormidiaceae | 3892 | 1.49% |
| Spirosomaceae | 11196 | 1.50% | Sphingomonadaceae | 2758 | 1.06% |
| Cyanobacteriia_unclassified | 9595 | 1.28% | Bacteria_unclassified | 2728 | 1.05% |
| Propionibacteriaceae | 8601 | 1.15% | Sphingobacteriaceae | 2692 | 1.03% |
| Actinobacteria_unclassified | 8189 | 1.10% | Beijerinckiaceae | 2397 | 0.92% |
| Leptolyngbyaceae | 7824 | 1.05% | Nocardioidaceae | 1930 | 0.74% |
| Solirubrobacterales_unclassified | 7723 | 1.03% | Solirubrobacteraceae | 1850 | 0.71% |
| Cyanobacteriales_unclassified | 5677 | 0.76% | Cyanobacteriales_unclassified | 1844 | 0.71% |
| Deinococcaceae | 5423 | 0.73% | Caulobacteraceae | 1701 | 0.65% |
| Sphingomonadaceae | 5270 | 0.71% | WD2101_soil_group | 1500 | 0.58% |
| Frankiales_unclassified | 4951 | 0.66% | Frankiales_unclassified | 1366 | 0.52% |
| Nocardiaceae | 4580 | 0.61% | Nakamurellaceae | 1282 | 0.49% |
| Chloroflexales_unclassified | 4199 | 0.56% | Actinobacteria_unclassified | 1242 | 0.48% |
| Sphingobacteriaceae | 4145 | 0.55% | Nostocaceae | 1200 | 0.46% |
| Armatimonadales_fa | 4025 | 0.54% | Pyrinomonadaceae | 1014 | 0.39% |
| 67-14 | 3962 | 0.53% | AKIW781 | 1001 | 0.38% |
| LWQ8 | 3372 | 0.45% | Propionibacteriaceae | 898 | 0.34% |
| Nakamurellaceae | 3151 | 0.42% | uncultured | 851 | 0.33% |
| uncultured | 2796 | 0.37% | Deinococcaceae | 808 | 0.31% |
| Flavobacteriaceae | 2752 | 0.37% | Solirubrobacterales_unclassified | 777 | 0.30% |
| AKIW781 | 2606 | 0.35% | Solibacteraceae | 700 | 0.27% |
| Frankiaceae | 2368 | 0.32% | Intrasporangiaceae | 606 | 0.23% |
| Intrasporangiaceae | 1960 | 0.26% | JG30-KF-CM45 | 579 | 0.22% |
| WD2101_soil_group | 1847 | 0.25% | 67-14 | 551 | 0.21% |
| uncultured_fa | 1728 | 0.23% | Bryobacteraceae | 531 | 0.20% |
| Phormidesmiaceae | 1657 | 0.22% | Micromonosporaceae | 508 | 0.19% |
| Gemmatimonadaceae | 1584 | 0.21% | C0119_fa | 487 | 0.19% |
| Cytophagales_unclassified | 1579 | 0.21% | Ilumatobacteraceae | 446 | 0.17% |
| Beijerinckiaceae | 1504 | 0.20% | Comamonadaceae | 439 | 0.17% |
|  |  |  |  |  |  |
| **Granite** | | | **Gneiss** | | |
| **Family** | **n. reads** | **% reads** | **Family** | **n. reads** | **% reads** |
| JG30-KF-CM45 | 1420 | 0.19% | Nocardiaceae | 406 | 0.16% |
| Iamiaceae | 1415 | 0.19% | Frankiaceae | 403 | 0.15% |
| Comamonadaceae | 1248 | 0.17% | Cyanobacteriia_unclassified | 380 | 0.15% |
| Proteobacteria_unclassified | 1246 | 0.17% | Leptolyngbyaceae | 374 | 0.14% |
| Kineosporiaceae | 1213 | 0.16% | Iamiaceae | 367 | 0.14% |
| Trueperaceae | 1165 | 0.16% | Trueperaceae | 356 | 0.14% |
| Chloroflexaceae | 1159 | 0.16% | Thermoleophilaceae | 349 | 0.13% |
| Cytophagaceae | 969 | 0.13% | Caldilineaceae | 340 | 0.13% |
| Caulobacteraceae | 954 | 0.13% | Alphaproteobacteria_unclassified | 336 | 0.13% |
| Oxalobacteraceae | 788 | 0.11% | Thermobaculaceae | 330 | 0.13% |
| Dermacoccaceae | 699 | 0.09% | Gemmatimonadaceae | 325 | 0.12% |
| Micromonosporaceae | 696 | 0.09% | Proteobacteria_unclassified | 262 | 0.10% |
| Tepidisphaeraceae | 682 | 0.09% | Acidobacteriae_unclassified | 248 | 0.10% |
| Xanthomonadaceae | 648 | 0.09% | Bacteroidia_unclassified | 242 | 0.09% |
| Micrococcaceae | 638 | 0.09% | Blastocatellia_unclassified | 237 | 0.09% |
| Acidobacteriaceae_(Subgroup_1) | 602 | 0.08% | Rubrobacteriaceae | 231 | 0.09% |
| Burkholderiaceae | 588 | 0.08% | Thermoleophilia_unclassified | 230 | 0.09% |
| Xanthobacteraceae | 570 | 0.08% | Burkholderiales_unclassified | 198 | 0.08% |
| Rhodobacteraceae | 561 | 0.08% | KD4-96_fa | 190 | 0.07% |
| Roseiflexaceae | 533 | 0.07% | Burkholderiaceae | 188 | 0.07% |
| Solibacteraceae | 495 | 0.07% | Xanthobacteraceae | 186 | 0.07% |
| unknown_unclassified | 475 | 0.06% | Cytophagales_unclassified | 167 | 0.06% |
| Acidimicrobiia_unclassified | 462 | 0.06% | Actinobacteriota_unclassified | 135 | 0.05% |
| Rubrobacteriaceae | 457 | 0.06% | Tepidisphaeraceae | 130 | 0.05% |
| Polyangiaceae | 430 | 0.06% | Phormidesmiaceae | 123 | 0.05% |
| Saccharimonadales_fa | 418 | 0.06% | Rhizobiaceae | 112 | 0.04% |
| Blastocatellia_unclassified | 399 | 0.05% | Chitinophagales_unclassified | 108 | 0.04% |
| Burkholderiales_unclassified | 385 | 0.05% | Acidimicrobiia_unclassified | 107 | 0.04% |
| Bacteroidia_unclassified | 374 | 0.05% | Kineosporiaceae | 104 | 0.04% |
| RBG-13-54-9_fa | 365 | 0.05% | Gaiellaceae | 99 | 0.04% |
| Rhodothermaceae | 326 | 0.04% | Microtrichales_unclassified | 87 | 0.03% |
| Saccharimonadales_unclassified | 313 | 0.04% | Rhodobacteraceae | 87 | 0.03% |
| env.OPS_17 | 310 | 0.04% | Micrococcaceae | 86 | 0.03% |
| Pyrinomonadaceae | 291 | 0.04% | LWQ8 | 83 | 0.03% |
| Bryobacteraceae | 289 | 0.04% | TK10_fa | 78 | 0.03% |
| Actinobacteriota_unclassified | 288 | 0.04% | Silvanigrellaceae | 71 | 0.03% |
| Thermoleophilia_unclassified | 285 | 0.04% | unknown_unclassified | 71 | 0.03% |
| C0119_fa | 270 | 0.04% | Gemmataceae | 70 | 0.03% |
| Geodermatophilaceae | 240 | 0.03% | Herpetosiphonaceae | 64 | 0.02% |
| Parcubacteria_fa | 221 | 0.03% | Parcubacteria_fa | 59 | 0.02% |
| Caldilineaceae | 180 | 0.02% | Rhodothermaceae | 59 | 0.02% |
| Alphaproteobacteria_unclassified | 169 | 0.02% | SC-I-84 | 56 | 0.02% |
| Microtrichales_unclassified | 140 | 0.02% | WPS-2_fa | 51 | 0.02% |
| **Granite** | | | **Gneiss** | | |
| **Family** | **n. reads** | **% reads** | **Family** | **n. reads** | **% reads** |
| Cyclobacteriaceae | 136 | 0.02% | Ktedonobacteria_unclassified | 48 | 0.02% |
| Ktedonobacteraceae | 131 | 0.02% | Tepidisphaerales_unclassified | 48 | 0.02% |
| Ilumatobacteraceae | 129 | 0.02% | Chthoniobacteraceae | 47 | 0.02% |
| Isosphaeraceae | 121 | 0.02% | Armatimonadales_unclassified | 46 | 0.02% |
| Microscillaceae | 117 | 0.02% | Chloroflexia_unclassified | 44 | 0.02% |
| Rickettsiaceae | 113 | 0.02% | Micrococcales_unclassified | 44 | 0.02% |
| Rhizobiaceae | 107 | 0.01% | Longimicrobiaceae | 43 | 0.02% |
| Gemmataceae | 98 | 0.01% | Rhizobiales_unclassified | 43 | 0.02% |
| Geminicoccaceae | 88 | 0.01% | AKYG1722 | 41 | 0.02% |
| Tepidisphaerales_unclassified | 88 | 0.01% | Cytophagaceae | 40 | 0.02% |
| Rhizobiales_unclassified | 85 | 0.01% | Oxalobacteraceae | 39 | 0.01% |
| Thermoleophilaceae | 83 | 0.01% | Xanthomonadaceae | 39 | 0.01% |
| Nannocystaceae | 80 | 0.01% | Geodermatophilaceae | 38 | 0.01% |
| Nitrosomonadaceae | 80 | 0.01% | A21b | 37 | 0.01% |
| IMCC26256_fa | 73 | 0.01% | Ardenticatenaceae | 35 | 0.01% |
| Herpetosiphonaceae | 71 | 0.01% | Saccharimonadales_fa | 33 | 0.01% |
| Rickettsiales_fa | 70 | 0.01% | Cyclobacteriaceae | 32 | 0.01% |
| Haliangiaceae | 68 | 0.01% | Geminicoccaceae | 31 | 0.01% |
| Polyangiales_unclassified | 65 | 0.01% | Microbacteriaceae | 30 | 0.01% |
| Armatimonadales_unclassified | 60 | 0.01% | Nitrospiraceae | 30 | 0.01% |
| KD4-96_fa | 58 | 0.01% | Roseiflexaceae | 30 | 0.01% |
| Micropepsaceae | 54 | 0.01% | RBG-13-54-9_fa | 29 | 0.01% |
| Patescibacteria_unclassified | 54 | 0.01% | uncultured_fa | 28 | 0.01% |
| Rhodanobacteraceae | 52 | 0.01% | Vicinamibacterales_unclassified | 27 | 0.01% |
| 11-24_fa | 49 | 0.01% | Vicinamibacteraceae | 25 | 0.01% |
| BIrii41 | 46 | 0.01% | JG30-KF-CM66_fa | 23 | 0.01% |
| Streptomycetaceae | 46 | 0.01% | Kapabacteriales_fa | 23 | 0.01% |
| Chitinophagales_unclassified | 43 | 0.01% | Acidobacteriota_unclassified | 22 | 0.01% |
| Phaselicystidaceae | 43 | 0.01% | Sutterellaceae | 22 | 0.01% |
| Sporichthyaceae | 40 | 0.01% | Flavobacteriaceae | 20 | 0.01% |
| Corynebacteriales_unclassified | 38 | 0.01% | Phycisphaeraceae | 20 | 0.01% |
| Microbacteriaceae | 37 | 0.00% | Polyangiaceae | 20 | 0.01% |
| TK10_fa | 37 | 0.00% | BIrii41 | 19 | 0.01% |
| Berkelbacteria_fa | 35 | 0.00% | Isosphaeraceae | 17 | 0.01% |
| Flavobacteriales_unclassified | 33 | 0.00% | Devosiaceae | 16 | 0.01% |
| JG30-KF-CM66_fa | 33 | 0.00% | Microscillaceae | 16 | 0.01% |
| Micrococcales_unclassified | 30 | 0.00% | TRA3-20 | 16 | 0.01% |
| Acidobacteriae_unclassified | 29 | 0.00% | Propionibacteriales_unclassified | 15 | 0.01% |
| Chthoniobacteraceae | 28 | 0.00% | Haliangiaceae | 14 | 0.01% |
| Polyangia_unclassified | 27 | 0.00% | IMCC26256_fa | 14 | 0.01% |
| WPS-2_fa | 25 | 0.00% | Anaerolineae_unclassified | 13 | 0.00% |
| Saccharimonadaceae | 21 | 0.00% | Chloroflexi_unclassified | 13 | 0.00% |
| AKYG1722 | 20 | 0.00% | Euzebyaceae | 9 | 0.00% |
| **Granite** | | | **Gneiss** | | |
| **Family** | **n. reads** | **% reads** | **Family** | **n. reads** | **% reads** |
| Bdellovibrionaceae | 20 | 0.00% | Gammaproteobacteria_unclassified | 9 | 0.00% |
| SC-I-84 | 20 | 0.00% | SJA-28_fa | 9 | 0.00% |
| Paracaedibacteraceae | 18 | 0.00% | Chthonomonadaceae | 8 | 0.00% |
| Propionibacteriales_unclassified | 18 | 0.00% | SAR324_clade(Marine_group_B)_fa | 8 | 0.00% |
| Caedibacteraceae | 17 | 0.00% | B1-7BS | 7 | 0.00% |
| Microtrichales_fa | 17 | 0.00% | Saccharimonadales_unclassified | 7 | 0.00% |
| Silvanigrellaceae | 17 | 0.00% | Sericytochromatia_fa | 7 | 0.00% |
| mle1-27_fa | 15 | 0.00% | Acidothermaceae | 6 | 0.00% |
| Bacteroidota_unclassified | 14 | 0.00% | Oligoflexaceae | 6 | 0.00% |
| Chthonomonadaceae | 14 | 0.00% | P2-11E_fa | 6 | 0.00% |
| Weeksellaceae | 14 | 0.00% | Verrucomicrobiaceae | 6 | 0.00% |
| Acidothermaceae | 13 | 0.00% | Polyangiales_unclassified | 5 | 0.00% |
| Chloroflexi_unclassified | 13 | 0.00% | Bacteroidaceae | 4 | 0.00% |
| Gammaproteobacteria_unclassified | 12 | 0.00% | Corynebacteriales_unclassified | 4 | 0.00% |
| Vicinamibacterales_unclassified | 12 | 0.00% | Myxococcaceae | 4 | 0.00% |
| Opitutaceae | 11 | 0.00% | Polyangia_unclassified | 4 | 0.00% |
| Vampirovibrionales_fa | 11 | 0.00% | Rickettsiaceae | 4 | 0.00% |
| Chloroflexia_unclassified | 10 | 0.00% | Caedibacteraceae | 3 | 0.00% |
| Verrucomicrobiae_unclassified | 10 | 0.00% | Lineage_IIa_fa | 3 | 0.00% |
| A21b | 9 | 0.00% | Phaselicystidaceae | 3 | 0.00% |
| A4b | 9 | 0.00% | Firmicutes_unclassified | 2 | 0.00% |
| Acidobacteriota_unclassified | 9 | 0.00% | Gitt-GS-136_fa | 2 | 0.00% |
| Myxococcaceae | 9 | 0.00% | Porphyromonadaceae | 2 | 0.00% |
| Sandaracinaceae | 9 | 0.00% | Sphingobacteriales_unclassified | 2 | 0.00% |
| 0319-6G20_fa | 8 | 0.00% | Streptococcaceae | 2 | 0.00% |
| Gaiellales_unclassified | 8 | 0.00% | Subgroup_7_fa | 2 | 0.00% |
| Gallionellaceae | 8 | 0.00% | Sumerlaeaceae | 2 | 0.00% |
| Armatimonadota_unclassified | 7 | 0.00% | Weeksellaceae | 2 | 0.00% |
| Devosiaceae | 7 | 0.00% | Gaiellales_unclassified | 1 | 0.00% |
| Rhizobiales_Incertae_Sedis | 7 | 0.00% |  |  |  |
| B12-WMSP1_fa | 6 | 0.00% |  |  |  |
| Corynebacteriaceae | 6 | 0.00% |  |  |  |
| Kapabacteriales_fa | 6 | 0.00% |  |  |  |
| Sumerlaeaceae | 5 | 0.00% |  |  |  |
| Sutterellaceae | 5 | 0.00% |  |  |  |
| TSBb06_fa | 5 | 0.00% |  |  |  |
| Deinococci_unclassified | 4 | 0.00% |  |  |  |
| Phycisphaeraceae | 4 | 0.00% |  |  |  |
| Candidatus_Kaiserbacteria_fa | 3 | 0.00% |  |  |  |
| Legionellaceae | 3 | 0.00% |  |  |  |
| Verrucomicrobiales_unclassified | 3 | 0.00% |  |  |  |
| Acetobacterales_unclassified | 2 | 0.00% |  |  |  |
| Cellulomonadaceae | 2 | 0.00% |  |  |  |
| Chthoniobacterales_unclassified | 2 | 0.00% |  |  |  |
| Clostridiaceae | 2 | 0.00% |  |  |  |
| TRA3-20 | 2 | 0.00% |  |  |  |
| Vicinamibacteraceae | 2 | 0.00% |  |  |  |
| **Marble** | | | **Moraine** | | |
| **Family** | **n. reads** | **% reads** | **Family** | **n. reads** | **% reads** |
| Chroococcidiopsaceae | 110552 | 31.07% | Blastocatellaceae | 564050 | 30.11% |
| Blastocatellaceae | 99209 | 27.88% | Pseudonocardiaceae | 336533 | 17.96% |
| Spirosomaceae | 44303 | 12.45% | Solirubrobacteraceae | 140967 | 7.52% |
| Trueperaceae | 10889 | 3.06% | Solirubrobacterales_unclassified | 116762 | 6.23% |
| Acetobacteraceae | 8231 | 2.31% | Nocardioidaceae | 70467 | 3.76% |
| Abditibacteriaceae | 8186 | 2.30% | Actinobacteria_unclassified | 67362 | 3.60% |
| Euzebyaceae | 6325 | 1.78% | JG30-KF-CM45 | 65097 | 3.47% |
| Bacteria_unclassified | 5899 | 1.66% | Chitinophagaceae | 45146 | 2.41% |
| Pseudonocardiaceae | 4952 | 1.39% | uncultured_fa | 36217 | 1.93% |
| Hymenobacteraceae | 3567 | 1.00% | Frankiales_unclassified | 31541 | 1.68% |
| AKIW781 | 3364 | 0.95% | Leptolyngbyaceae | 27878 | 1.49% |
| Cyanobacteriales_unclassified | 3359 | 0.94% | Abditibacteriaceae | 25012 | 1.34% |
| Solirubrobacterales_unclassified | 2510 | 0.71% | uncultured | 22714 | 1.21% |
| Chitinophagaceae | 2475 | 0.70% | Bacteria_unclassified | 21774 | 1.16% |
| Nocardioidaceae | 2417 | 0.68% | Thermoleophilia_unclassified | 19516 | 1.04% |
| uncultured | 2386 | 0.67% | 67-14 | 17434 | 0.93% |
| 67-14 | 2299 | 0.65% | Chroococcidiopsaceae | 16371 | 0.87% |
| Solirubrobacteraceae | 2190 | 0.62% | Acidimicrobiia_unclassified | 13027 | 0.70% |
| Pyrinomonadaceae | 2123 | 0.60% | Propionibacteriaceae | 12770 | 0.68% |
| Thermoleophilia_unclassified | 2001 | 0.56% | Ilumatobacteraceae | 12043 | 0.64% |
| Actinobacteria_unclassified | 1712 | 0.48% | Gemmatimonadaceae | 9308 | 0.50% |
| Cytophagaceae | 1534 | 0.43% | Acetobacteraceae | 8967 | 0.48% |
| Rhodobacteraceae | 1531 | 0.43% | Armatimonadales_fa | 8471 | 0.45% |
| Blastocatellia_unclassified | 1438 | 0.40% | Intrasporangiaceae | 8214 | 0.44% |
| Rhizobiaceae | 1373 | 0.39% | KD4-96_fa | 7680 | 0.41% |
| Cyanobacteriia_unclassified | 1360 | 0.38% | Pyrinomonadaceae | 7373 | 0.39% |
| JG30-KF-CM45 | 1243 | 0.35% | Frankiaceae | 6870 | 0.37% |
| WD2101_soil_group | 1099 | 0.31% | Cyanobacteriia_unclassified | 6690 | 0.36% |
| Frankiales_unclassified | 1025 | 0.29% | Rubrobacteriaceae | 6558 | 0.35% |
| Caulobacteraceae | 993 | 0.28% | Sphingobacteriaceae | 6060 | 0.32% |
| Beijerinckiaceae | 848 | 0.24% | Trueperaceae | 6017 | 0.32% |
| Propionibacteriaceae | 794 | 0.22% | B12-WMSP1_fa | 5863 | 0.31% |
| Bryobacteraceae | 652 | 0.18% | LWQ8 | 5647 | 0.30% |
| Frankiaceae | 645 | 0.18% | Deinococcaceae | 5277 | 0.28% |
| Sphingomonadaceae | 639 | 0.18% | AKIW781 | 5169 | 0.28% |
| Microscillaceae | 615 | 0.17% | Nocardiaceae | 4887 | 0.26% |
| Armatimonadales_fa | 557 | 0.16% | Thermoleophilaceae | 4695 | 0.25% |
| Thermoleophilaceae | 542 | 0.15% | Saccharimonadales_unclassified | 4676 | 0.25% |
| uncultured_fa | 510 | 0.14% | Micrococcaceae | 4654 | 0.25% |
| **Marble** | | | **Moraine** | | |
| **Family** | **n. reads** | **% reads** | **Family** | **n. reads** | **% reads** |
| AKYG1722 | 500 | 0.14% | Iamiaceae | 4608 | 0.25% |
| Ilumatobacteraceae | 485 | 0.14% | Hymenobacteraceae | 4391 | 0.23% |
| Cytophagales_unclassified | 448 | 0.13% | IMCC26256_fa | 4356 | 0.23% |
| MWH-CFBk5 | 444 | 0.12% | Spirosomaceae | 3708 | 0.20% |
| Rhodothermaceae | 434 | 0.12% | C0119_fa | 3664 | 0.20% |
| Intrasporangiaceae | 423 | 0.12% | Comamonadaceae | 3377 | 0.18% |
| Micrococcaceae | 399 | 0.11% | Sphingomonadaceae | 3358 | 0.18% |
| Gaiellaceae | 345 | 0.10% | Thermobaculaceae | 3139 | 0.17% |
| KD4-96_fa | 336 | 0.09% | Micrococcales_unclassified | 2877 | 0.15% |
| Proteobacteria_unclassified | 323 | 0.09% | Beijerinckiaceae | 2846 | 0.15% |
| Sphingobacteriaceae | 321 | 0.09% | WD2101_soil_group | 2839 | 0.15% |
| Leptolyngbyaceae | 320 | 0.09% | Actinobacteriota_unclassified | 2617 | 0.14% |
| Saccharimonadales_unclassified | 300 | 0.08% | Blastocatellia_unclassified | 2315 | 0.12% |
| Microtrichales_unclassified | 262 | 0.07% | Euzebyaceae | 1919 | 0.10% |
| Rubrobacteriaceae | 234 | 0.07% | Flavobacteriaceae | 1835 | 0.10% |
| Acidobacteriae_unclassified | 215 | 0.06% | Parcubacteria_fa | 1823 | 0.10% |
| Caldilineaceae | 205 | 0.06% | Caldilineaceae | 1673 | 0.09% |
| Cyclobacteriaceae | 194 | 0.05% | Phormidiaceae | 1449 | 0.08% |
| Nostocaceae | 192 | 0.05% | Planococcaceae | 1418 | 0.08% |
| Acidimicrobiia_unclassified | 190 | 0.05% | Microtrichales_unclassified | 1393 | 0.07% |
| Thermobaculaceae | 189 | 0.05% | Gitt-GS-136_fa | 1392 | 0.07% |
| Iamiaceae | 157 | 0.04% | A4b | 1315 | 0.07% |
| Chitinophagales_unclassified | 143 | 0.04% | Rhodobacteraceae | 1299 | 0.07% |
| Xanthomonadaceae | 128 | 0.04% | Chloroflexaceae | 1294 | 0.07% |
| Gemmataceae | 120 | 0.03% | Kineosporiaceae | 1212 | 0.06% |
| LWQ8 | 120 | 0.03% | AKYG1722 | 1038 | 0.06% |
| unknown_unclassified | 117 | 0.03% | Cytophagales_unclassified | 1006 | 0.05% |
| Gaiellales_unclassified | 105 | 0.03% | Nostocaceae | 976 | 0.05% |
| Actinobacteriota_unclassified | 99 | 0.03% | SJA-28_fa | 972 | 0.05% |
| Chloroflexi_unclassified | 95 | 0.03% | Gaiellaceae | 931 | 0.05% |
| Comamonadaceae | 91 | 0.03% | Nakamurellaceae | 872 | 0.05% |
| Sutterellaceae | 90 | 0.03% | Chloroflexia_unclassified | 849 | 0.05% |
| Micromonosporaceae | 89 | 0.03% | Solibacteraceae | 847 | 0.05% |
| BIrii41 | 74 | 0.02% | Xanthomonadaceae | 811 | 0.04% |
| Herpetosiphonaceae | 72 | 0.02% | Saccharimonadales_fa | 762 | 0.04% |
| Kineosporiaceae | 70 | 0.02% | TK10_fa | 678 | 0.04% |
| Gemmatimonadaceae | 67 | 0.02% | Sutterellaceae | 644 | 0.03% |
| Xanthomonadales_unclassified | 67 | 0.02% | Pseudanabaenaceae | 640 | 0.03% |
| Bdellovibrionaceae | 66 | 0.02% | Gemmataceae | 627 | 0.03% |
| Sporichthyaceae | 62 | 0.02% | Bryobacteraceae | 561 | 0.03% |
| TK10_fa | 59 | 0.02% | Cyclobacteriaceae | 515 | 0.03% |
| Alphaproteobacteria_unclassified | 52 | 0.01% | Caulobacteraceae | 511 | 0.03% |
| IMCC26256_fa | 51 | 0.01% | Oxalobacteraceae | 511 | 0.03% |
| **Marble** | | | **Moraine** | | |
| **Family** | **n. reads** | **% reads** | **Family** | **n. reads** | **% reads** |
| Bacteroidia_unclassified | 50 | 0.01% | B10-SB3A_fa | 488 | 0.03% |
| C0119_fa | 46 | 0.01% | Chthoniobacteraceae | 476 | 0.03% |
| Devosiaceae | 43 | 0.01% | Propionibacteriales_unclassified | 463 | 0.02% |
| Vicinamibacteraceae | 39 | 0.01% | Micromonosporaceae | 435 | 0.02% |
| Deinococcaceae | 34 | 0.01% | Sporichthyaceae | 431 | 0.02% |
| Isosphaeraceae | 33 | 0.01% | Nitrosomonadaceae | 407 | 0.02% |
| Unknown_Family | 33 | 0.01% | Acidobacteriae_fa | 403 | 0.02% |
| Armatimonadales_unclassified | 29 | 0.01% | unknown_unclassified | 393 | 0.02% |
| Gitt-GS-136_fa | 29 | 0.01% | Microbacteriaceae | 362 | 0.02% |
| Polyangiaceae | 26 | 0.01% | Corynebacteriales_unclassified | 358 | 0.02% |
| Rhizobiales_unclassified | 23 | 0.01% | Xanthobacteraceae | 339 | 0.02% |
| JG30-KF-CM66_fa | 18 | 0.01% | Rhizobiales_unclassified | 325 | 0.02% |
| Ardenticatenaceae | 17 | 0.00% | Rhodanobacteraceae | 290 | 0.02% |
| Saccharimonadales_fa | 16 | 0.00% | JG30-KF-CM66_fa | 284 | 0.02% |
| Deinococci_unclassified | 14 | 0.00% | Microscillaceae | 273 | 0.01% |
| Oxalobacteraceae | 14 | 0.00% | Bacteroidia_unclassified | 256 | 0.01% |
| SC-I-84 | 14 | 0.00% | Ktedonobacteraceae | 253 | 0.01% |
| Haliangiaceae | 13 | 0.00% | Rhodothermaceae | 247 | 0.01% |
| Parcubacteria_fa | 13 | 0.00% | Proteobacteria_unclassified | 240 | 0.01% |
| Planctomycetes_unclassified | 13 | 0.00% | Gaiellales_unclassified | 235 | 0.01% |
| SAR324_clade(Marine_group_B)_fa | 13 | 0.00% | Vicinamibacteraceae | 234 | 0.01% |
| Micrococcales_unclassified | 12 | 0.00% | Tepidisphaeraceae | 232 | 0.01% |
| Phaselicystidaceae | 12 | 0.00% | Nitrospiraceae | 212 | 0.01% |
| Tepidisphaeraceae | 12 | 0.00% | Pirellulaceae | 183 | 0.01% |
| 0319-7L14_fa | 11 | 0.00% | Rhizobiales_Incertae_Sedis | 170 | 0.01% |
| Bacteroidota_unclassified | 11 | 0.00% | Streptomycetaceae | 169 | 0.01% |
| Chthoniobacteraceae | 11 | 0.00% | Burkholderiales_unclassified | 162 | 0.01% |
| Deinococcales_unclassified | 11 | 0.00% | Longimicrobiaceae | 157 | 0.01% |
| TRA3-20 | 11 | 0.00% | Isosphaeraceae | 152 | 0.01% |
| Chloroflexia_unclassified | 10 | 0.00% | Chloroflexi_unclassified | 148 | 0.01% |
| Porphyromonadaceae | 9 | 0.00% | MB-A2-108_fa | 141 | 0.01% |
| Burkholderiales_unclassified | 8 | 0.00% | Herpetosiphonaceae | 118 | 0.01% |
| NS9_marine_group | 7 | 0.00% | Gammaproteobacteria_unclassified | 117 | 0.01% |
| Xanthobacteraceae | 7 | 0.00% | Patescibacteria_unclassified | 112 | 0.01% |
| Bacillaceae | 6 | 0.00% | Kapabacteriales_fa | 107 | 0.01% |
| Polyangiales_unclassified | 6 | 0.00% | Geminicoccaceae | 96 | 0.01% |
| Pseudanabaenaceae | 6 | 0.00% | TRA3-20 | 93 | 0.00% |
| B1-7BS | 5 | 0.00% | Chitinophagales_unclassified | 92 | 0.00% |
| Geminicoccaceae | 5 | 0.00% | Bdellovibrionaceae | 91 | 0.00% |
| Myxococcaceae | 4 | 0.00% | Geodermatophilaceae | 88 | 0.00% |
| Rickettsiaceae | 4 | 0.00% | Sericytochromatia_fa | 75 | 0.00% |
| Rickettsiales_fa | 4 | 0.00% | 0319-7L14_fa | 66 | 0.00% |
| Tepidisphaerales_unclassified | 4 | 0.00% | Burkholderiaceae | 66 | 0.00% |
| **Marble** | | | **Moraine** | | |
| **Family** | **n. reads** | **% reads** | **Family** | **n. reads** | **% reads** |
| WPS-2_fa | 4 | 0.00% | vadinHA49_fa | 66 | 0.00% |
| Acidobacteriaceae_(Subgroup_1) | 3 | 0.00% | Acidimicrobiaceae | 61 | 0.00% |
| Burkholderiaceae | 3 | 0.00% | Acidobacteriae_unclassified | 60 | 0.00% |
| Gaiellales_fa | 3 | 0.00% | Berkelbacteria_fa | 56 | 0.00% |
| mle1-27_fa | 3 | 0.00% | Subgroup_7_fa | 54 | 0.00% |
| Nitrosomonadaceae | 3 | 0.00% | Alphaproteobacteria_unclassified | 53 | 0.00% |
| Phycisphaerae_unclassified | 3 | 0.00% | Armatimonadales_unclassified | 52 | 0.00% |
| Diplorickettsiaceae | 2 | 0.00% | Microtrichaceae | 51 | 0.00% |
| Parcubacteria_unclassified | 2 | 0.00% | Rhizobiaceae | 51 | 0.00% |
| Propionibacteriales_unclassified | 2 | 0.00% | Diplorickettsiaceae | 47 | 0.00% |
| Sandaracinaceae | 2 | 0.00% | Anaerolineaceae | 46 | 0.00% |
| Thermomicrobiales_unclassified | 2 | 0.00% | Sumerlaeaceae | 44 | 0.00% |

***Table S8****. Taxonomic assignment at the eukaryotic division-level of the ASVs with cumulative abundance calculated for each substrate type.*

| **Granite** | | |  | |  | | **Gneiss** | | | |  |
| --- | --- | --- | --- | --- | --- | --- | --- | --- | --- | --- | --- |
| **Division** | **n. reads** | **% reads** | |  | |  | | **Division** | **n. reads** | **% reads** | |
| Chlorophyta | 1395307 | 61.20% | |  | |  | | Chlorophyta | 483408 | 63.18% | |
| Metazoa | 555705 | 24.38% | |  | |  | | Metazoa | 242798 | 31.74% | |
| Ciliophora | 252995 | 11.10% | |  | |  | | Cercozoa | 21618 | 2.83% | |
| Ochrophyta | 29245 | 1.28% | |  | |  | | Ciliophora | 6260 | 0.82% | |
| Cercozoa | 22814 | 1.00% | |  | |  | | Eukaryota_unclassified | 6043 | 0.79% | |
| Opisthokonta_unclassified | 10671 | 0.47% | |  | |  | | Fungi | 2683 | 0.35% | |
| Lobosa | 5238 | 0.23% | |  | |  | | Opisthokonta_unclassified | 1858 | 0.24% | |
| Fungi | 3915 | 0.17% | |  | |  | | Lobosa | 200 | 0.03% | |
| Eukaryota_unclassified | 3529 | 0.15% | |  | |  | | Ochrophyta | 74 | 0.01% | |
| Streptophyta | 333 | 0.01% | |  | |  | | Pseudofungi | 69 | 0.01% | |
| Dinoflagellata | 27 | 0.00% | |  | |  | | Streptophyta | 55 | 0.01% | |
| Opalozoa | 9 | 0.00% | |  | |  | | Opalozoa | 9 | 0.00% | |
| **Marble** | | |  | |  | | **Moraine** | | | |  |
| **Division** | **n. reads** | **% reads** | |  | |  | | **Division** | **n. reads** | **% reads** | |
| Chlorophyta | 1005651 | 46.34% | |  | |  | | Chlorophyta | 1988246 | 66.73% | |
| Metazoa | 718026 | 33.08% | |  | |  | | Cercozoa | 409745 | 13.75% | |
| Cercozoa | 310928 | 14.33% | |  | |  | | Metazoa | 261472 | 8.78% | |
| Ciliophora | 84106 | 3.88% | |  | |  | | Lobosa | 121032 | 4.06% | |
| Eukaryota_unclassified | 19443 | 0.90% | |  | |  | | Ciliophora | 74857 | 2.51% | |
| Fungi | 16745 | 0.77% | |  | |  | | Eukaryota_unclassified | 51478 | 1.73% | |
| Opisthokonta_unclassified | 7294 | 0.34% | |  | |  | | Streptophyta | 32053 | 1.08% | |
| Ochrophyta | 2768 | 0.13% | |  | |  | | Fungi | 7700 | 0.26% | |
| Streptophyta | 2016 | 0.09% | |  | |  | | Apicomplexa | 6694 | 0.22% | |
| Lobosa | 1522 | 0.07% | |  | |  | | Opisthokonta_unclassified | 5614 | 0.19% | |
|  |  |  | |  | |  | |  |  |  | |
| **Marble** | | |  | |  | | **Moraine** | | | |  |
| **Division** | **n. reads** | **% reads** | |  | |  | | **Division** | **n. reads** | **% reads** | |
| Archaeplastida_unclassified | 1089 | 0.05% | |  | |  | | Centroheliozoa | 4724 | 0.16% | |
| Dinoflagellata | 467 | 0.02% | |  | |  | | Ochrophyta | 4410 | 0.15% | |
| Apicomplexa | 163 | 0.01% | |  | |  | | Dinoflagellata | 3697 | 0.12% | |
| Opalozoa | 150 | 0.01% | |  | |  | | Cryptophyta | 3539 | 0.12% | |
| Discoba | 7 | 0.00% | |  | |  | | Conosa | 3155 | 0.11% | |
|  |  |  | |  | |  | | Opalozoa | 474 | 0.02% | |
|  |  |  | |  | |  | | Pseudofungi | 318 | 0.01% | |
|  |  |  | |  | |  | | Discoba | 141 | 0.00% | |

***Table S9. Taxonomic assignment at the eukaryotic family-level of the ASVs with cumulative abundance calculated for each substrate type.***

| **Granite** | | | **Gneiss** | | | |  |
| --- | --- | --- | --- | --- | --- | --- | --- |
| **Family** | **n. reads** | **% reads** | | **Family** | **n. reads** | **% reads** | |
| Microthamniales_X | 947963 | 41.58% | | Microthamniales_X | 305363 | 39.91% | |
| Prasiolales_X | 378942 | 16.62% | | Prasiolales_X | 143485 | 18.75% | |
| Rotifera_XX | 304706 | 13.37% | | Rotifera_XX | 127867 | 16.71% | |
| Tardigrada_XX | 165459 | 7.26% | | Tardigrada_XX | 88690 | 11.59% | |
| Nassulida | 126306 | 5.54% | | Metazoa_unclassified | 26241 | 3.43% | |
| Chilodonellidae | 98401 | 4.32% | | Watanabea-Clade_X | 20989 | 2.74% | |
| Metazoa_unclassified | 68112 | 2.99% | | Cercozoa_unclassified | 8703 | 1.14% | |
| Watanabea-Clade_X | 38133 | 1.67% | | Eukaryota_unclassified | 6043 | 0.79% | |
| Xanthophyceae_XX | 29245 | 1.28% | | Sandonidae | 5960 | 0.78% | |
| Chlamydomonadales_X | 23154 | 1.02% | | Rhogostoma-lineage | 5673 | 0.74% | |
| Collembola | 16814 | 0.74% | | Trebouxiophyceae_unclassified | 5631 | 0.74% | |
| Oxytrichidae | 16389 | 0.72% | | Chlorophyta_unclassified | 4891 | 0.64% | |
| Rhogostoma-lineage | 13747 | 0.60% | | Oxytrichidae | 2620 | 0.34% | |
| Opisthokonta_unclassified | 10671 | 0.47% | | Chilodonellidae | 2520 | 0.33% | |
| Sessilida | 4940 | 0.22% | | Chlamydomonadales_X | 2497 | 0.33% | |
| Cercozoa_unclassified | 4335 | 0.19% | | Opisthokonta_unclassified | 1858 | 0.24% | |
| Endostelium-lineage | 3967 | 0.17% | | Tremellomycetes | 1394 | 0.18% | |
| Trebouxiophyceae_unclassified | 3728 | 0.16% | | Colpodida | 774 | 0.10% | |
| Eukaryota_unclassified | 3529 | 0.15% | | Cercomonadidae | 669 | 0.09% | |
| Sandonidae | 3422 | 0.15% | | Pezizomycotina_unclassified | 667 | 0.09% | |
| Pleurostomatida | 2775 | 0.12% | | Glissomonadida_unclassified | 574 | 0.08% | |
| Tokophryidae | 2601 | 0.11% | | Chlamydomonadales_unclassified | 333 | 0.04% | |
| Chytridiomycetes | 2243 | 0.10% | | Dothideomycetes | 220 | 0.03% | |
| Ulotrichales_X | 1991 | 0.09% | | Coccomyxaceae | 219 | 0.03% | |
| LOS7N/I-lineage | 1236 | 0.05% | | Chytridiomycetes | 206 | 0.03% | |
| Cercomonadidae | 1052 | 0.05% | | Endostelium-lineage | 153 | 0.02% | |
| Pezizomycotina_unclassified | 970 | 0.04% | | Platyophryida | 137 | 0.02% | |
| Ciliophora_unclassified | 788 | 0.03% | | Fungi_unclassified | 104 | 0.01% | |
|  |  |  | |  |  |  | |
| **Granite** | | | **Gneiss** | | | |  |
| **Family** | **n. reads** | **% reads** | | **Family** | **n. reads** | **% reads** | |
| Chlorophyta_unclassified | 702 | 0.03% | | Ciliophora_unclassified | 80 | 0.01% | |
| Ulvophyceae_unclassified | 689 | 0.03% | | Xanthophyceae_XX | 74 | 0.01% | |
| Arachnida | 528 | 0.02% | | Oomycota_X_unclassified | 69 | 0.01% | |
| Embryophyceae_XX | 333 | 0.01% | | Pleurostomatida | 64 | 0.01% | |
| Colpodida | 306 | 0.01% | | Embryophyceae_XX | 55 | 0.01% | |
| Tremellomycetes | 252 | 0.01% | | Lecanoromycetes | 54 | 0.01% | |
| Platyophryida | 210 | 0.01% | | Dileptidae | 41 | 0.01% | |
| Halteriidae | 201 | 0.01% | | Allapsidae | 39 | 0.01% | |
| Dothideomycetes | 158 | 0.01% | | LOS7N/I-lineage | 39 | 0.01% | |
| Glissomonadida_unclassified | 142 | 0.01% | | Cystobasidiomycetes | 24 | 0.00% | |
| Fungi_unclassified | 110 | 0.00% | | Tokophryidae | 17 | 0.00% | |
| Annelida_XX | 86 | 0.00% | | Sordariomycetes | 14 | 0.00% | |
| Lecanoromycetes | 86 | 0.00% | | MAST-12C_X | 9 | 0.00% | |
| Cryomonadida_unclassified | 77 | 0.00% | | Arcellinida-A3 | 8 | 0.00% | |
| Saccharomycetales | 50 | 0.00% | | Nassulida | 4 | 0.00% | |
| Tubulinea_unclassified | 35 | 0.00% | | Litostomatea_unclassified | 3 | 0.00% | |
| Glissomonadida_X | 34 | 0.00% | |  |  |  | |
| Taphrinomycetes | 30 | 0.00% | |  |  |  | |
| Tetrahymenida | 27 | 0.00% | |  |  |  | |
| Dinophyceae_unclassified | 23 | 0.00% | |  |  |  | |
| Litostomatea_XX | 19 | 0.00% | |  |  |  | |
| Rhodomelaceae | 18 | 0.00% | |  |  |  | |
| Colpodea_X_unclassified | 12 | 0.00% | |  |  |  | |
| Evaginogenida | 12 | 0.00% | |  |  |  | |
| Sordariomycetes | 11 | 0.00% | |  |  |  | |
| MAST-12C_X | 9 | 0.00% | |  |  |  | |
| OLIGO2 | 8 | 0.00% | |  |  |  | |
| **Marble** | | | **Moraine** | | | |  |
| **Family** | **n. reads** | **% reads** | | **Family** | **n. reads** | **% reads** | |
| Microthamniales_X | 916175 | 42.21% | | Microthamniales_X | 1729690 | 58.06% | |
| Rotifera_XX | 462990 | 21.33% | | Sandonidae | 260396 | 8.74% | |
| Tardigrada_XX | 208567 | 9.61% | | Rotifera_XX | 128598 | 4.32% | |
| Rhogostoma-lineage | 188226 | 8.67% | | Metazoa_unclassified | 116861 | 3.92% | |
| Sandonidae | 114553 | 5.28% | | LOS7N/I-lineage | 111234 | 3.73% | |
| Chilodonellidae | 73592 | 3.39% | | Rhogostoma-lineage | 108817 | 3.65% | |
| Prasiolales_X | 71511 | 3.29% | | Chlamydomonadales_X | 108198 | 3.63% | |
| Metazoa_unclassified | 45579 | 2.10% | | Prasiolales_X | 95817 | 3.22% | |
| Eukaryota_unclassified | 19443 | 0.90% | | Chilodonellidae | 52336 | 1.76% | |
| Watanabea-Clade_X | 10117 | 0.47% | | Eukaryota_unclassified | 51478 | 1.73% | |
| Chytridiomycetes | 7445 | 0.34% | | Embryophyceae_XX | 32053 | 1.08% | |
| Opisthokonta_unclassified | 7294 | 0.34% | | Chlorellales_X | 29345 | 0.98% | |
| Cercomonadidae | 5504 | 0.25% | | Cercozoa_unclassified | 23995 | 0.81% | |
| **Marble** | | | **Moraine** | | | |  |
| **Family** | **n. reads** | **% reads** | | **Family** | **n. reads** | **% reads** | |
| Pezizomycotina_unclassified | 3560 | 0.16% | | Watanabea-Clade_X | 15895 | 0.53% | |
| Pleurostomatida | 3022 | 0.14% | | Trachelocercidae | 8939 | 0.30% | |
| Fungi_unclassified | 2881 | 0.13% | | Allapsidae | 8869 | 0.30% | |
| Colpodida | 2831 | 0.13% | | Endostelium-lineage | 8712 | 0.29% | |
| Tremellomycetes | 2647 | 0.12% | | Annelida_XX | 8698 | 0.29% | |
| Trebouxiophyceae_unclassified | 2455 | 0.11% | | Trebouxiophyceae_unclassified | 7570 | 0.25% | |
| Chlorophyta_unclassified | 2400 | 0.11% | | Alphamonaceae | 6481 | 0.22% | |
| Oxytrichidae | 2348 | 0.11% | | Tardigrada_XX | 5775 | 0.19% | |
| Polar-centric-Mediophyceae | 2176 | 0.10% | | Opisthokonta_unclassified | 5614 | 0.19% | |
| Embryophyceae_XX | 2016 | 0.09% | | Centroheliozoa_X_unclassified | 4724 | 0.16% | |
| Chlamydomonadales_X | 1623 | 0.07% | | Cryptomonadales_X | 3539 | 0.12% | |
| Pyramimonadales_XX | 1346 | 0.06% | | Polar-centric-Mediophyceae | 3165 | 0.11% | |
| Arcellinida-A3 | 1293 | 0.06% | | Evaginogenida | 2752 | 0.09% | |
| Archaeplastida_unclassified | 1089 | 0.05% | | Litostomatea_XX | 2742 | 0.09% | |
| Litostomatea_XX | 1043 | 0.05% | | Cercomonadidae | 2625 | 0.09% | |
| Allapsidae | 702 | 0.03% | | Platyophryida | 2597 | 0.09% | |
| Xanthophyceae_XX | 592 | 0.03% | | Glissomonadida_unclassified | 2386 | 0.08% | |
| Spirotrichea_unclassified | 566 | 0.03% | | Pezizomycotina_unclassified | 2330 | 0.08% | |
| Glissomonadida_unclassified | 555 | 0.03% | | Acramoebidae | 1922 | 0.06% | |
| Novel-clade-2 | 484 | 0.02% | | Dino-Group-I-Clade-1 | 1695 | 0.06% | |
| Cryomonadida_unclassified | 427 | 0.02% | | Ciliophora_unclassified | 1601 | 0.05% | |
| Cercozoa_unclassified | 406 | 0.02% | | Filosa-Sarcomonadea_unclassified | 1461 | 0.05% | |
| Ciliophora_unclassified | 396 | 0.02% | | Oxytrichidae | 1335 | 0.04% | |
| Dinophyceae_unclassified | 376 | 0.02% | | Xanthophyceae_XX | 1187 | 0.04% | |
| Annelida_XX | 368 | 0.02% | | Colpodida | 1182 | 0.04% | |
| Dileptidae | 299 | 0.01% | | Enoplea_X | 1133 | 0.04% | |
| Insecta | 295 | 0.01% | | Fungi_unclassified | 1094 | 0.04% | |
| Maxillopoda | 227 | 0.01% | | Dothideomycetes | 1086 | 0.04% | |
| LOS7N/I-lineage | 167 | 0.01% | | Tremellomycetes | 1064 | 0.04% | |
| MAST-12C_X | 150 | 0.01% | | Dinophyceae_unclassified | 907 | 0.03% | |
| Lecanoromycetes | 101 | 0.00% | | Ulotrichales_X | 795 | 0.03% | |
| Dino-Group-I-Clade-1 | 86 | 0.00% | | Heterocapsaceae | 667 | 0.02% | |
| Colpodellida_unclassified | 82 | 0.00% | | Variosea_XX | 665 | 0.02% | |
| Gregarinidae | 81 | 0.00% | | Chytridiomycetes | 616 | 0.02% | |
| Eurotiomycetes | 67 | 0.00% | | Taphrinomycetes | 615 | 0.02% | |
| Endostelium-lineage | 62 | 0.00% | | Arcellinida-A3 | 575 | 0.02% | |
| Filosa-Thecofilosea_unclassified | 56 | 0.00% | | Strombidinopsidae | 562 | 0.02% | |
| Exobasidiomycetes | 39 | 0.00% | | Chlorophyta_unclassified | 492 | 0.02% | |
| Chlamydomonadales_unclassified | 24 | 0.00% | | Tubulinea_unclassified | 480 | 0.02% | |
| Spongomonadidae | 15 | 0.00% | | Lecanoromycetes | 461 | 0.02% | |
| Tetrahymenida | 9 | 0.00% | | Nassulida | 444 | 0.01% | |
| Vahlkampfiidae | 7 | 0.00% | | Ceratiaceae | 428 | 0.01% | |
| **Marble** | | | **Moraine** | | | |  |
| **Family** | **n. reads** | **% reads** | | **Family** | **n. reads** | **% reads** | |
| Heterocapsaceae | 5 | 0.00% | | Schizoplasmodiids | 414 | 0.01% | |
| Pezizomycotina_X | 5 | 0.00% | | Pseudodendromonadales_X | 402 | 0.01% | |
|  |  |  | | Glissomonadida_X | 354 | 0.01% | |
|  |  |  | | Cercomonadida_unclassified | 330 | 0.01% | |
|  |  |  | | Archosauria | 311 | 0.01% | |
|  |  |  | | Saccharomycetales | 234 | 0.01% | |
|  |  |  | | Limnofilidae | 230 | 0.01% | |
|  |  |  | | Oomycota_XX | 212 | 0.01% | |
|  |  |  | | Chlamydomonadales_unclassified | 185 | 0.01% | |
|  |  |  | | Flamella-lineage | 149 | 0.01% | |

***Table S10. Number of reads and ASVs in each module, with percentages relative to the whole network dataset, identified with the network analysis.***

|  | **n. reads** | **% of reads** | **n. ASVs** | **% of ASVs** | **n. bac ASVs** | **% bac ASVs** | **n. euk ASV** | **% euk ASVs** | **n. bac reads** | **n. euk reads** |
| --- | --- | --- | --- | --- | --- | --- | --- | --- | --- | --- |
| M1 | 225416 | 11.01% | 44 | 7.77% | 37 | 84.09% | 7 | 15.91% | 62446 | 162970 |
| M2 | 403884 | 19.74% | 60 | 10.60% | 58 | 96.67% | 2 | 3.33% | 350142 | 53742 |
| M3 | 112933 | 5.52% | 107 | 18.90% | 106 | 99.07% | 1 | 0.93% | 110336 | 2597 |
| M4 | 89470 | 4.37% | 71 | 12.54% | 62 | 87.32% | 9 | 12.68% | 51445 | 38025 |
| M5 | 8291 | 0.41% | 16 | 2.83% | 16 | 100.00% | 0 | 0.00% | 8291 | 0 |
| M6 | 188358 | 9.21% | 84 | 14.84% | 83 | 98.81% | 1 | 1.19% | 185188 | 3170 |
| M7 | 81559 | 3.99% | 72 | 12.72% | 64 | 88.89% | 8 | 11.11% | 51398 | 30161 |
| M8 | 177059 | 8.65% | 38 | 6.71% | 35 | 92.11% | 3 | 7.89% | 46924 | 690 |
| M9 | 278904 | 13.63% | 4 | 0.71% | 0 | 0.00% | 4 | 100.00% | 0 | 278904 |
| M10 | 431806 | 21.10% | 34 | 6.01% | 23 | 67.65% | 11 | 32.35% | 29466 | 402340 |
| M11 | 48510 | 2.37% | 36 | 6.36% | 32 | 88.89% | 4 | 11.11% | 402340 | 9593 |

***Table S11. Taxonomic assignment at the family level of the ASV with cumulative abundances calculated for each module (M).***

| **M1** | | | | **M2** | | | | | **M3** | | | | | | | |
| --- | --- | --- | --- | --- | --- | --- | --- | --- | --- | --- | --- | --- | --- | --- | --- | --- |
| **Family** | **n ASVs** | **n reads** | **Abund.** | **Family** | **n ASVs** | **n reads** | **Abund.** | | **Family** | | | **n ASVs** | | **n reads** | | **Abund.** |
| Nassulida | 2 | 126197 | 55.98% | Pseudonocardiaceae | 26 | 255313 | 63.21% | | Chroococcidiopsaceae | | | 25 | | 57095 | | 50.56% |
| Rotifera_XX | 2 | 34578 | 15.34% | Sandonidae | 2 | 53742 | 13.31% | | Blastocatellaceae | | | 16 | | 25513 | | 22.59% |
| Nostocaceae | 14 | 28075 | 12.45% | Blastocatellaceae | 8 | 43995 | 10.89% | | Spirosomaceae | | | 5 | | 7077 | | 6.27% |
| Phormidiaceae | 6 | 23977 | 10.64% | Solirubrobacterales_uncl. | 4 | 23784 | 5.89% | | Euzebyaceae | | | 7 | | 4380 | | 3.88% |
| Cyanobacteriales_uncl. | 1 | 3629 | 1.61% | 67-14 | 2 | 6544 | 1.62% | | Trueperaceae | | | 12 | | 3935 | | 3.48% |
| Solirubrobacteraceae | 2 | 1350 | 0.60% | Nocardioidaceae | 4 | 6127 | 1.52% | | Abditibacteriaceae | | | 6 | | 2747 | | 2.43% |
| Chytridiomycetes | 1 | 920 | 0.41% | Actinobacteria_uncl. | 3 | 2844 | 0.70% | | Opisthokonta_uncl. | | | 1 | | 2597 | | 2.30% |
| Cytophagales_uncl. | 1 | 913 | 0.41% | Thermoleophilaceae | 1 | 2810 | 0.70% | | Pseudonocardiaceae | | | 2 | | 1387 | | 1.23% |
| Chroococcidiopsaceae | 2 | 904 | 0.40% | Thermobaculaceae | 1 | 2679 | 0.66% | | Hymenobacteraceae | | | 3 | | 960 | | 0.85% |
| Sandonidae | 1 | 793 | 0.35% | Rubrobacteriaceae | 1 | 1816 | 0.45% | | AKIW781 | | | 2 | | 931 | | 0.82% |
| Cyanobacteriia_uncl. | 3 | 773 | 0.34% | KD4-96_fa | 2 | 1536 | 0.38% | | Cyanobacteriales_uncl. | | | 4 | | 827 | | 0.73% |
| Sphingomonadaceae | 2 | 625 | 0.28% | Euzebyaceae | 2 | 1106 | 0.27% | | Bacteria_uncl. | | | 5 | | 759 | | 0.67% |
| Cytophagaceae | 1 | 554 | 0.25% | Thermoleophilia_uncl. | 1 | 779 | 0.19% | | Thermoleophilia_uncl. | | | 2 | | 662 | | 0.59% |
| Spirosomaceae | 1 | 525 | 0.23% | JG30-KF-CM45 | 1 | 380 | 0.09% | | uncultured | | | 1 | | 455 | | 0.40% |
| Chilodonellidae | 1 | 482 | 0.21% | Iamiaceae | 1 | 216 | 0.05% | | Actinobacteria_uncl. | | | 1 | | 441 | | 0.39% |
| Acetobacteraceae | 2 | 453 | 0.20% | Frankiales_uncl. | 1 | 213 | 0.05% | | Pyrinomonadaceae | | | 2 | | 425 | | 0.38% |
| Nocardioidaceae | 1 | 400 | 0.18% |  |  |  |  | | Blastocatellia_uncl. | | | 2 | | 421 | | 0.37% |
| Flavobacteriaceae | 1 | 268 | 0.12% |  |  |  |  | | Acetobacteraceae | | | 3 | | 413 | | 0.37% |
|  |  |  |  |  |  |  |  | | MWH-CFBk5 | | | 1 | | 389 | | 0.34% |
|  |  |  |  |  |  |  |  | | Microscillaceae | | | 1 | | 338 | | 0.30% |
|  |  |  |  |  |  |  |  | | Nocardioidaceae | | | 1 | | 299 | | 0.26% |
|  |  |  |  |  |  |  |  | | AKYG1722 | | | 1 | | 295 | | 0.26% |
|  |  |  |  |  |  |  |  | | Intrasporangiaceae | | | 1 | | 156 | | 0.14% |
|  |  |  |  |  |  |  |  | | JG30-KF-CM45 | | | 1 | | 153 | | 0.14% |
|  |  |  |  |  |  |  |  | | Cyanobacteriia_uncl. | | | 1 | | 150 | | 0.13% |
|  |  |  |  |  |  |  |  | | Solirubrobacteraceae | | | 1 | | 128 | | 0.11% |
|  |  |  |  |  |  |  |  | |  | | |  | |  | |  |
| **M4** | | | | **M5** | | | | | **M6** | | | | | | | |
| **Family** | **n ASVs** | **n reads** | **Abund.** | **Family** | **n ASVs** | **n reads** | | **Abund.** | **Family** | | | **n ASVs** | | **n reads** | | **Abund.** |
| Nostocaceae | 25 | 33368 | 37.30% | Nostocaceae | 7 | 4277 | | 51.59% | Solirubrobacteraceae | | | 11 | | 62479 | | 33.17% |
| Watanabea-Clade_X | 1 | 12779 | 14.28% | Phormidesmiaceae | 2 | 1399 | | 16.87% | JG30-KF-CM45 | | | 14 | | 28309 | | 15.03% |
| Rotifera_XX | 1 | 9781 | 10.93% | Chroococcidiopsaceae | 2 | 1083 | | 13.06% | Actinobacteria_uncl. | | | 3 | | 19813 | | 10.52% |
| Sessilida | 1 | 4765 | 5.33% | Spirosomaceae | 1 | 720 | | 8.68% | Frankiales_uncl. | | | 4 | | 14741 | | 7.83% |
| Prasiolales_X | 2 | 4241 | 4.74% | Acetobacteraceae | 2 | 468 | | 5.64% | uncultured_fa | | | 3 | | 13818 | | 7.34% |
| Leptolyngbyaceae | 5 | 3438 | 3.84% | Cyanobacteriia_uncl. | 1 | 267 | | 3.22% | Solirubrobacterales_uncl. | | | 12 | | 12005 | | 6.37% |
| Opisthokonta_uncl. | 1 | 3424 | 3.83% | Bacteria_uncl. | 1 | 77 | | 0.93% | Bacteria_uncl. | | | 5 | | 4953 | | 2.63% |
| Solirubrobacteraceae | 4 | 3035 | 3.39% |  |  |  | |  | Blastocatellaceae | | | 2 | | 4347 | | 2.31% |
| Blastocatellaceae | 8 | 2967 | 3.32% |  |  |  | |  | Gemmatimonadaceae | | | 2 | | 4313 | | 2.29% |
| Cyanobacteriia_uncl. | 3 | 1825 | 2.04% |  |  |  | |  | Thermoleophilia_uncl. | | | 9 | | 3731 | | 1.98% |
| Tardigrada_XX | 1 | 1544 | 1.73% |  |  |  | |  | Sandonidae | | | 1 | | 3170 | | 1.68% |
| Rhogostoma-lineage | 2 | 1491 | 1.67% |  |  |  | |  | Nocardioidaceae | | | 2 | | 3005 | | 1.60% |
| Chroococcidiopsaceae | 2 | 968 | 1.08% |  |  |  | |  | uncultured | | | 2 | | 2636 | | 1.40% |
| Acetobacteraceae | 2 | 877 | 0.98% |  |  |  | |  | Intrasporangiaceae | | | 3 | | 2439 | | 1.29% |
| Sphingomonadaceae | 2 | 863 | 0.96% |  |  |  | |  | B12-WMSP1_fa | | | 2 | | 1718 | | 0.91% |
| Bacteria_uncl. | 2 | 815 | 0.91% |  |  |  | |  | Acidimicrobiia_uncl. | | | 1 | | 1635 | | 0.87% |
| Spirosomaceae | 3 | 698 | 0.78% |  |  |  | |  | Micrococcales_uncl. | | | 2 | | 1326 | | 0.70% |
| Chloroflexales_uncl. | 1 | 682 | 0.76% |  |  |  | |  | IMCC26256_fa | | | 1 | | 1276 | | 0.68% |
| Chloroflexaceae | 1 | 612 | 0.68% |  |  |  | |  | Chitinophagaceae | | | 1 | | 1130 | | 0.60% |
| Frankiales_uncl. | 1 | 519 | 0.58% |  |  |  | |  | Frankiaceae | | | 1 | | 453 | | 0.24% |
| Actinobacteria_uncl. | 1 | 326 | 0.36% |  |  |  | |  | Armatimonadales_fa | | | 1 | | 410 | | 0.22% |
| Pseudonocardiaceae | 1 | 270 | 0.30% |  |  |  | |  | Propionibacteriaceae | | | 1 | | 401 | | 0.21% |
| Chitinophagaceae | 1 | 182 | 0.20% |  |  |  | |  | Nocardiaceae | | | 1 | | 250 | | 0.13% |
|  |  |  |  |  |  |  | |  |  | | |  | |  | |  |
|  |  |  |  |  |  |  | |  |  | | |  | |  | |  |
|  |  |  |  |  |  |  | |  |  | | |  | |  | |  |
|  |  |  |  |  |  |  | |  |  | | |  | |  | |  |
|  |  |  |  |  |  |  | |  |  | | |  | |  | |  |
| **M7** | | | | **M8** | | | | | **M9** | | | | | | | |
| **Family** | **n ASVs** | **n reads** | **Abund.** | **Family** | **n ASVs** | **n reads** | | **Abund.** | **Family** | | | **n ASVs** | | **n reads** | | **Abund.** |
| Spirosomaceae | 17 | 31516 | 38.64% | Sandonidae | 3 | 95632 | | 54.01% | Prasiolales_X | | | 2 | | 152513 | | 54.68% |
| Chroococcidiopsaceae | 25 | 12044 | 14.77% | Solirubrobacterales_uncl. | 7 | 27213 | | 15.37% | Microthamniales_X | | | 2 | | 126391 | | 45.32% |
| Rhogostoma-lineage | 1 | 9415 | 11.54% | Blastocatellaceae | 9 | 20334 | | 11.48% |  | | |  | |  | |  |
| Chytridiomycetes | 1 | 7198 | 8.83% | Nocardioidaceae | 2 | 7440 | | 4.20% |  | | |  | |  | |  |
| Cercomonadidae | 1 | 5408 | 6.63% | uncultured_fa | 1 | 4926 | | 2.78% |  | | |  | |  | |  |
| Sandonidae | 2 | 3975 | 4.87% | JG30-KF-CM45 | 1 | 4457 | | 2.52% |  | | |  | |  | |  |
| Blastocatellaceae | 7 | 3076 | 3.77% | Ilumatobacteraceae | 1 | 4372 | | 2.47% |  | | |  | |  | |  |
| Opisthokonta_uncl. | 1 | 3037 | 3.72% | Propionibacteriaceae | 4 | 4294 | | 2.43% |  | | |  | |  | |  |
| Cytophagaceae | 1 | 1065 | 1.31% | Solirubrobacteraceae | 3 | 3392 | | 1.92% |  | | |  | |  | |  |
| Hymenobacteraceae | 1 | 778 | 0.95% | Chitinophagaceae | 1 | 1889 | | 1.07% |  | | |  | |  | |  |
| Acetobacteraceae | 1 | 752 | 0.92% | 67-14 | 1 | 1402 | | 0.79% |  | | |  | |  | |  |
| Tardigrada_XX | 1 | 626 | 0.77% | KD4-96_fa | 2 | 900 | | 0.51% |  | | |  | |  | |  |
| Colpodida | 1 | 502 | 0.62% | Acidimicrobiia_uncl. | 1 | 353 | | 0.20% |  | | |  | |  | |  |
| Thermoleophilaceae | 1 | 496 | 0.61% | Saccharimonadales_fa | 1 | 259 | | 0.15% |  | | |  | |  | |  |
| Cyanobacteriales_uncl. | 2 | 471 | 0.58% | LWQ8 | 1 | 196 | | 0.11% |  | | |  | |  | |  |
| AKIW781 | 1 | 411 | 0.50% |  |  |  | |  |  | | |  | |  | |  |
| Trueperaceae | 1 | 223 | 0.27% |  |  |  | |  |  | | |  | |  | |  |
| Pyrinomonadaceae | 1 | 136 | 0.17% |  |  |  | |  |  | | |  | |  | |  |
| Solirubrobacterales_uncl. | 2 | 132 | 0.16% |  |  |  | |  |  | | |  | |  | |  |
| 67-14 | 2 | 107 | 0.13% |  |  |  | |  |  | | |  | |  | |  |
| Thermoleophilia_uncl. | 1 | 106 | 0.13% |  |  |  | |  |  | | |  | |  | |  |
| Gaiellaceae | 1 | 85 | 0.10% |  |  |  | |  |  | | |  | |  | |  |
| **M10** | | | | **M11** | | | | |  |  |  | |  | |  | |
| **Family** | **n ASVs** | **n reads** | **Abund.** | **Family** | **n ASVs** | **n reads** | | **Abund.** |  | | |  | |  | |  |
| Microthamniales_X | 7 | 322569 | 74.70% | Blastocatellaceae | 9 | 24112 | | 49.71% |  | | |  | |  | |  |
| Rotifera_XX | 1 | 40036 | 9.27% | Chlorophyta_uncl. | 1 | 4399 | | 9.07% |  | | |  | |  | |  |
| Watanabea-Clade_X | 3 | 39735 | 9.20% | Chlamydomonadales_X | 2 | 3256 | | 6.71% |  | | |  | |  | |  |
|  |  |  |  |  |  |  | |  |  | | |  | |  | |  |
| **M10** | | | | **M11** | | | | |  |  |  | |  | |  | |
| **Family** | **n ASVs** | **n reads** | **Abund.** | **Family** | **n ASVs** | **n reads** | | **Abund.** |  | | |  | |  | |  |
| Chroococcidiopsaceae | 4 | 17513 | 4.06% | Bacteria_uncl. | 3 | 2329 | | 4.80% |  | | |  | |  | |  |
| Acetobacteraceae | 8 | 5108 | 1.18% | Acetobacteraceae | 3 | 1963 | | 4.05% |  | | |  | |  | |  |
| Blastocatellaceae | 4 | 4601 | 1.07% | Eukaryota_uncl. | 1 | 1938 | | 4.00% |  | | |  | |  | |  |
| Spirosomaceae | 2 | 995 | 0.23% | 67-14 | 1 | 1709 | | 3.52% |  | | |  | |  | |  |
| Gemmatimonadaceae | 1 | 358 | 0.08% | Acidobacteriaceae_(Subgroup_1) | 1 | 1585 | | 3.27% |  | | |  | |  | |  |
| Comamonadaceae | 1 | 269 | 0.06% | Hymenobacteraceae | 3 | 1248 | | 2.57% |  | | |  | |  | |  |
| Nostocaceae | 1 | 239 | 0.06% | Deinococcaceae | 1 | 1101 | | 2.27% |  | | |  | |  | |  |
| uncultured | 1 | 216 | 0.05% | Caulobacteraceae | 1 | 932 | | 1.92% |  | | |  | |  | |  |
| Hymenobacteraceae | 1 | 167 | 0.04% | Beijerinckiaceae | 2 | 790 | | 1.63% |  | | |  | |  | |  |
|  |  |  |  | Frankiaceae | 1 | 592 | | 1.22% |  | | |  | |  | |  |
|  |  |  |  | Sphingobacteriaceae | 1 | 522 | | 1.08% |  | | |  | |  | |  |
|  |  |  |  | Chitinophagaceae | 1 | 466 | | 0.96% |  | | |  | |  | |  |
|  |  |  |  | Propionibacteriaceae | 1 | 439 | | 0.90% |  | | |  | |  | |  |
|  |  |  |  | Nakamurellaceae | 1 | 309 | | 0.64% |  | | |  | |  | |  |
|  |  |  |  | Abditibacteriaceae | 1 | 299 | | 0.62% |  | | |  | |  | |  |
|  |  |  |  | Armatimonadales_fa | 1 | 264 | | 0.54% |  | | |  | |  | |  |
|  |  |  |  | Solirubrobacteraceae | 1 | 257 | | 0.53% |  | | |  | |  | |  |

***Table S12. Taxonomic assignment at the phylum level of the ASV with cumulative abundance calculated for each module (M).***

| **M1** | | | | **M2** | | | | **M3** | | | |
| --- | --- | --- | --- | --- | --- | --- | --- | --- | --- | --- | --- |
| **Phylum/Division** | **n. ASVs** | **n. reads** | **Abund.** | **Phylum/Division** | **n. ASVs** | **n. reads** | **Abund.** | **Phylum/Division** | **n. ASVs** | **n. reads** | **Abund.** |
| Ciliophora | 3 | 126679 | 56.20% | Actinobacteriota | 46 | 301552 | 74.66% | Cyanobacteriota | 30 | 58072 | 51.42% |
| Cyanobacteriota | 26 | 57358 | 25.45% | Cercozoa | 2 | 53742 | 13.31% | Acidobacteriota | 20 | 26359 | 23.34% |
| Metazoa | 2 | 34578 | 15.34% | Acidobacteriota | 8 | 43995 | 10.89% | Bacteroidota | 11 | 9219 | 8.16% |
| Bacteroidota | 4 | 2260 | 1.00% | Chloroflexota | 4 | 4595 | 1.14% | Actinobacteriota | 15 | 7453 | 6.60% |
| Actinobacteriota | 3 | 1750 | 0.78% |  |  |  |  | Deinococcota | 12 | 3935 | 3.48% |
| Pseudomonadota | 4 | 1078 | 0.48% |  |  |  |  | Abditibacteriota | 6 | 2747 | 2.43% |
| Fungi | 1 | 920 | 0.41% |  |  |  |  | Opisthokonta_uncl. | 1 | 2597 | 2.30% |
| Cercozoa | 1 | 793 | 0.35% |  |  |  |  | Chloroflexota | 4 | 1379 | 1.22% |
|  |  |  |  |  |  |  |  | Bacteria_uncl. | 5 | 759 | 0.67% |
| **M4** | | | | **M5** | | | | **M6** | | | |
| **Phylum/Division** | **n. ASVs** | **n. reads** | **Abund.** | **Phylum/Division** | **n. ASVs** | **n. reads** | **Abund.** | **Phylum/Division** | **n. ASVs** | **n. reads** | **Abund.** |
| Cyanobacteriota | 35 | 39599 | 44.26% | Cyanobacteriota | 12 | 7026 | 84.74% | Actinobacteriota | 53 | 126190 | 66.99% |
| Chlorophyta | 3 | 17020 | 19.02% | Bacteroidota | 1 | 720 | 8.68% | Chloroflexota | 16 | 30027 | 15.94% |
| Metazoa | 2 | 11325 | 12.66% | Pseudomonadota | 2 | 468 | 5.64% | Patescibacteria | 3 | 13818 | 7.34% |
| Ciliophora | 1 | 4765 | 5.33% | Bacteria_uncl. | 1 | 77 | 0.93% | Bacteria_uncl. | 5 | 4953 | 2.63% |
| Actinobacteriota | 7 | 4150 | 4.64% |  |  |  |  | Acidobacteriota | 2 | 4347 | 2.31% |
| Opisthokonta_uncl. | 1 | 3424 | 3.83% |  |  |  |  | Gemmatimonadota | 2 | 4313 | 2.29% |
| Acidobacteriota | 8 | 2967 | 3.32% |  |  |  |  | Cercozoa | 1 | 3170 | 1.68% |
| Pseudomonadota | 4 | 1740 | 1.94% |  |  |  |  | Bacteroidota | 1 | 1130 | 0.60% |
| Cercozoa | 2 | 1491 | 1.67% |  |  |  |  | Armatimonadota | 1 | 410 | 0.22% |
| Chloroflexota | 2 | 1294 | 1.45% |  |  |  |  |  |  |  |  |
| Bacteroidota | 4 | 880 | 0.98% |  |  |  |  |  |  |  |  |
| Bacteria_uncl. | 2 | 815 | 0.91% |  |  |  |  |  |  |  |  |
|  |  |  |  |  |  |  |  |  |  |  |  |
|  |  |  |  |  |  |  |  |  |  |  |  |
|  |  |  |  |  |  |  |  |  |  |  |  |
| **M7** | | | | **M8** | | | | **M9** | | | |
| **Phylum/Division** | **n. ASVs** | **n. reads** | **Abund.** | **Phylum/Division** | **n. ASVs** | **n. reads** | **Abund.** | **Phylum/Division** | **n. ASVs** | **n. reads** | **Abund.** |
| Bacteroidota | 19 | 33359 | 40.90% | Cercozoa | 3 | 95632 | 54.01% | Chlorophyta | 4 | 278904 | 100.00% |
| Cercozoa | 4 | 18798 | 23.05% | Actinobacteriota | 19 | 48466 | 27.37% |  |  |  |  |
| Cyanobacteriota | 27 | 12515 | 15.34% | Acidobacteriota | 9 | 20334 | 11.48% |  |  |  |  |
| Fungi | 1 | 7198 | 8.83% | Patescibacteria | 3 | 5381 | 3.04% |  |  |  |  |
| Acidobacteriota | 8 | 3212 | 3.94% | Chloroflexota | 3 | 5357 | 3.03% |  |  |  |  |
| Opisthokonta_uncl. | 1 | 3037 | 3.72% | Bacteroidota | 1 | 1889 | 1.07% |  |  |  |  |
| Actinobacteriota | 7 | 926 | 1.14% |  |  |  |  |  |  |  |  |
| Pseudomonadota | 1 | 752 | 0.92% |  |  |  |  |  |  |  |  |
| Metazoa | 1 | 626 | 0.77% |  |  |  |  |  |  |  |  |
| Ciliophora | 1 | 502 | 0.62% |  |  |  |  |  |  |  |  |
| Chloroflexota | 1 | 411 | 0.50% |  |  |  |  |  |  |  |  |
| Deinococcota | 1 | 223 | 0.27% |  |  |  |  |  |  |  |  |
| **M10** | | | | **M11** | | | |  |  |  |  |
| **Phylum/Division** | **n. ASVs** | **n. reads** | **Abund.** | **Phylum** | **n. ASVs** | **n. reads** | **Abund.** |  |  |  |  |
| Chlorophyta | 10 | 362304 | 83.90% | Acidobacteriota | 10 | 25697 | 52.97% |  |  |  |  |
| Metazoa | 1 | 40036 | 9.27% | Chlorophyta | 3 | 7655 | 15.78% |  |  |  |  |
| Cyanobacteriota | 5 | 17752 | 4.11% | Pseudomonadota | 6 | 3685 | 7.60% |  |  |  |  |
| Pseudomonadota | 9 | 5377 | 1.25% | Actinobacteriota | 5 | 3306 | 6.82% |  |  |  |  |
| Acidobacteriota | 4 | 4601 | 1.07% | Bacteria_uncl. | 3 | 2329 | 4.80% |  |  |  |  |
| Bacteroidota | 4 | 1378 | 0.32% | Bacteroidota | 5 | 2236 | 4.61% |  |  |  |  |
| Gemmatimonadota | 1 | 358 | 0.08% | Eukaryota_uncl. | 1 | 1938 | 4.00% |  |  |  |  |
|  |  |  |  | Deinococcota | 1 | 1101 | 2.27% |  |  |  |  |
|  |  |  |  | Abditibacteriota | 1 | 299 | 0.62% |  |  |  |  |
|  |  |  |  | Armatimonadota | 1 | 264 | 0.54% |  |  |  |  |

***Table S13. Results of the Pearson moment correlation between the cumulative abundances of the ASVs in each module (M) and environmental variables.*** *Only correlations with p<0.01 are highlighted in bold, after correction for multiple hypothesis testing.*

| **Modules** | **Factor** | **r2** | **df** | ***adj. p*** | **Modules** | **Factor** | **r2** | **df** | ***adj. p*** |
| --- | --- | --- | --- | --- | --- | --- | --- | --- | --- |
| M1 | Cond. | -0.22 | 83 | 0.0432 | M8 | **pH** | -0.32 | 83 | 0.0034 |
| M2 | **Cond.** | 0.28 | 83 | 0.0085 |  | TOC | -0.32 | 83 | 0.0032 |
|  | **TOC** | -0.24 | 83 | 0.0287 |  | **PO_4_^3-^** | -0.33 | 83 | 0.0021 |
|  | **TN** | -0.43 | 83 | 5.01E-05 | M9 | N-NH_4_^+^ | 0.46 | 83 | 1.01E-05 |
| M3 | **pH** | 0.51 | 83 | 6.83E-07 |  | P-PO_4_^3-^ | 0.26 | 83 | 0.0184 |
|  | **N-NH_4_^+^** | -.026 | 83 | 0.0168 | M10 | **TOC** | 0.56 | 83 | 2.77E-08 |
|  | N-NO_3_^-^ | -0.27 | 83 | 0.0464 |  | **N-NH_4_^+^** | 0.35 | 83 | 0.0012 |
|  | **TN** | -0.28 | 83 | 0.0005 |  | **DW** | -0.26 | 83 | 0.016 |
|  | **P-PO_4_^3-^** | 0.68 | 83 | 8.55E-05 |  | TN | -0.25 | 83 | 0.0227 |
| M4 | **Cond.** | -0.26 | 83 | 0.0168 |  | TP | -0.24 | 83 | 0.0260 |
| M5 | **TN** | 0.26 | 83 | 0.0183 | M11 | **pH** | -0.30 | 83 | 0.0048 |
| M6 | **pH** | -0.26 | 83 | 0.0157 |  | **TOC** | 0.44 | 83 | 2.70E-05 |
|  | **Cond.** | -0.26 | 83 | 0.0184 |  | **N-NH_4_^+^** | 0.46 | 83 | 8.06E-06 |
|  | **TOC** | -0.21 | 83 | 0.0489 |  | **DW** | -0.30 | 83 | 0.0055 |
|  | TN | -0.27 | 83 | 0.0144 |  | **TN** | 0.38 | 83 | 0.0003 |
| M7 | **pH** | 0.52 | 83 | 4.40E-07 |  | TP | 0.25 | 83 | 0.0203 |
|  | **N-NH_4_^+^** | -0.25 | 83 | 0.0226 |  |  |  |  |  |
|  | **P-PO_4_^3-^** | -0.46 | 83 | 1.13E-05 |  |  |  |  |  |

***Table S14. Results of the Spearman rank correlation between the cumulative abundance of the ASVs in each module (M) and environmental variables.*** *Only correlations with p<0.01 are reported, after applying the holm correction for multiple hypothesis testing.*

| **Modules** | **Factor** | **Rho, ρ** | ***adj. p*** | **Modules** | **Factor** | **Rho, ρ** | ***adj. p*** |
| --- | --- | --- | --- | --- | --- | --- | --- |
| M1 | Cond. | -0.32 | 0.0025 | M7 | pH | 0.57 | 1.43E-08 |
|  | TOC | 0.41 | 0.0001 |  | P-PO_4_^3-^ | -0.60 | 1.75E-09 |
|  | N-NH_4_^+^ | 0.33 | 0.0018 | M8 | pH | -0.41 | 9.16E-05 |
|  | TN | 0.32 | 0.0031 |  | TOC | -0.53 | 2.39E-07 |
| M2 | TOC | -0.39 | 0.0002 |  | TN | -0.68 | 1.17E-12 |
|  | N-NH_4_^+^ | -0.34 | 0.0017 |  | P-PO_4_^3-^ | 0.32 | 0.0023 |
|  | DW | 0.31 | 0.0037 | M9 | N-NH_4_^+^ | 0.46 | 1.23E-05 |
|  | TN | -0.50 | 9.75E-07 |  | TN | 0.31 | 0.0040 |
| M3 | pH | 0.63 | 1.12E-10 |  | P-PO_4_^3-^ | 0.36 | 0.0008 |
|  | DW | 0.32 | 0.0025 | M10 | pH | -0.32 | 0.0031 |
|  | TN | 0.39 | 0.0002 |  | TOC | 0.54 | 8.45E-08 |
|  | P-PO_4_^3-^ | -0.59 | 3.31E-09 |  | N-NH_4_^+^ | 0.49 | 1.93E-06 |
|  | TN | 0.30 | 0.0049 |  | DW | -0.45 | 1.66E-05 |
|  | PO4 | 0.30 | 0.0048 |  | TN | 0.37 | 0.00051 |
| M4 | Cond. | -0.38 | 0.0004 | M11 | pH | -0.30 | 0.0048 |
|  | N-NH_4_^+^ | 0.35 | 0.0010 |  | TOC | 0.44 | 2.70E-05 |
|  | TN | 0.31 | 0.0029 |  | N-NH_4_^+^ | 0.46 | 8.06E-06 |
| M5 | Cond. | -0.35 | 0.0009 |  | DW | -0.30 | 0.0055 |
|  | N-NH_4_^+^ | 0.32 | 0.0025 |  | TN | 0.38 | 0.0003 |
|  | TN | 0.01 | 0.006 |  | TP | 0.25 | 0.0204 |
| M6 | pH | -0.53 | 2.09E-07 |  |  |  |  |
|  | TOC | -0.37 | 0.0006 |  |  |  |  |
|  | P-PO_4_^3-^ | 0.32 | 0.0031 |  |  |  |  |

***Table S15. Results of the Random Forests (RF) approach to the identification of the environmental explanatory variables for the distribution of each module across the sampled substrate types.*** *The environmental variables are reported in ranking order. Thresh. var. - number of variables that passed the Important Variable threshold; Interpr. var. - number of variables identified as important in interpreting the module (M) distribution. Interpretation variables are marked in bold.*

|  |  |  | **Environmental variable importance ranking in Random Forests interpretation** | | | | | | | | |  |
| --- | --- | --- | --- | --- | --- | --- | --- | --- | --- | --- | --- | --- |
| **Modules** | **thresh. var.** | **interp. var.** | **1** | **2** | **3** | **4** | **5** | **6** | **7** | **8** | **9** | |
| M1 | 4 | 3 | **Cond.** | **DW** | **TOC** | N-NH_4_^+^ |  |  |  |  |  | |
| M2 | 9 | 4 | **TN** | **pH** | **Cond.** | **N-NH_4_^+^** | TOC | P-PO_4_^3-^ | N-NO_3_^-^ | DW | TP | |
| M3 | 8 | 3 | **pH** | **P-PO_4_^3-^** | **TN** | TP | TOC | Cond. | N-NH_4_^+^ | DW |  | |
| M4 | 6 | 5 | **Cond.** | **N-NO_3_^-^** | **TN** | N-NH_4_^+^ | pH | TOC |  |  |  | |
| M5 | 9 | 3 | **TN** | **N-NH_4_^+^** | **Cond.** | TOC | DW | TP | P-PO_4_^3-^ | pH | N-NO_3_^-^ | |
| M6 | 8 | 4 | **Cond.** | **pH** | **TN** | **TOC** | P-PO_4_^3-^ | TP | N-NH_4_^+^ | N-NO_3_^-^ |  | |
| M7 | 8 | 5 | **P-PO_4_^3-^** | **pH** | **Cond.** | **N-NH_4_^+^** | **TN** | TOC | TP | N-NO_3_^-^ |  | |
| M8 | 6 | 4 | **TN** | **pH** | **TOC** | **TP** | N-NH_4_^+^ | P-PO_4_^3-^ |  |  |  | |
| M9 | 6 | 1 | **N-NH_4_^+^** | pH | DW | TN | P-PO_4_^3-^ | TOC |  |  |  | |
| M10 | 7 | 2 | **TOC** | **P-PO_4_^3-^** | N-NH_4_^+^ | TN | pH | TP |  |  |  | |
| M11 | 8 | 3 | **N-NH_4_^+^** | **pH** | **TP** | TOC | TN | Cond. | DW | P-PO_4_^3-^ |  | |

***Table S16.*** ***Table reporting the environmental variables selected using the different techniques (scatterplot inspection, Spearman rank correlation, Pearson moment correlation, Random Forests) and the selected variable plotted in Figure 6 for each module (M).*** *In the RF column the bold variables are those selected by the model.*

|  | M1 | | | | M2 | | | | M3 | | | | M4 | | | |
| --- | --- | --- | --- | --- | --- | --- | --- | --- | --- | --- | --- | --- | --- | --- | --- | --- |
| Var. ranking | Scatter | Pearson | Spearman | RF | Scatter | Pearson | Spearman | RF | Scatter | Pearson | Spearman | RF | Scatter | Pearson | Spearman | RF |
| 1 | **Cond.** | **Cond.** | **Cond.** | **Cond.** | **TN** | Cond. | TOC | **TN** | **pH** | **pH** | **pH** | **pH** | **Cond.** | **Cond.** | **Cond.** | **Cond.** |
| 2 |  |  | TOC | DW |  | TOC | N-NH_4_^+^ | **pH** | N-NH_4_^+^ | N-NH_4_^+^ | DW | **P-PO_4_^3-^** |  |  | N-NH_4_^+^ | N-NO_3_^-^ |
| 3 |  |  | N-NH_4_^+^ | TOC |  | **TN** | DW | **Cond.** | N-NO_3_^-^ | N-NO_3_^-^ | **TN** | **TN** |  |  | TN | TN |
| 4 |  |  | TN | N-NH_4_^+^ |  |  | **TN** | N-NH_4_^+^ | **TN** | **TN** | **P-PO_4_^3-^** | TP |  |  |  | N-NH_4_^+^ |
| 5 |  |  |  |  |  |  |  | TOC | DW | **P-PO_4_^3-^** |  | TOC |  |  |  | pH |
| 6 |  |  |  |  |  |  |  | P-PO_4_^3-^ | **P-PO_4_^3-^** |  |  | Cond. |  |  |  | TOC |
| 7 |  |  |  |  |  |  |  | N-NO_3_^-^ |  |  |  | N-NH_4_^+^ |  |  |  |  |
| 8 |  |  |  |  |  |  |  | DW |  |  |  | DW |  |  |  |  |
| 9 |  |  |  |  |  |  |  | TP |  |  |  |  |  |  |  |  |
| Selected var. | Cond. | | | | TN | | | | pH, TN, P-PO_4_^3-^ | | | | Cond. | | | |
|  | M5 | | | | M6 | | | | M7 | | | | M8 | | | |
| Var. ranking | Scatter | Pearson | Spearman | RF | Scatter | Pearson | Spearman | RF | Scatter | Pearson | Spearman | RF | Scatter | Pearson | Spearman | RF |
| 1 | Cond. | **TN** | Cond. | **TN** | **pH** | **pH** | **pH** | Cond. | **pH** | **pH** | **pH** | **P-PO_4_^3-^** | **pH** | **pH** | **pH** | **TN** |
| 2 | N-NO_3_^-^ |  | N-NH_4_^+^ | **N-NH_4_^+^** | Cond. | Cond. | **TOC** | **pH** | TOC | N-NH_4_^+^ | **P-PO_4_^3-^** | **pH** | Cond. | **TOC** | **TOC** | **pH** |
| 3 | **TN** |  | **TN** | **Cond.** | **TOC** | **TOC** | **TN** | **TN** | TN | **P-PO_4_^3-^** |  | **Cond.** | **TOC** | **TN** | **TN** | **TOC** |
| 4 |  |  |  | TOC | N-NO_3_^-^ |  | P-PO_4_^3-^ | **TOC** | **P-PO_4_^3-^** |  |  | **N-NH_4_^+^** | N-NH_4_^+^ |  | P-PO_4_^3-^ | **TP** |
| 5 |  |  |  | DW | **TN** |  |  | P-PO_4_^3-^ |  |  |  | **TN** | **TN** |  |  | N-NH_4_^+^ |
| 6 |  |  |  | TP |  |  |  | TP |  |  |  | TOC |  |  |  | P-PO_4_^3-^ |
| 7 |  |  |  | P-PO_4_^3-^ |  |  |  | N-NH_4_^+^ |  |  |  | TP |  |  |  |  |
| 8 |  |  |  | pH |  |  |  | N-NO_3_^-^ |  |  |  | N-NO_3_^-^ |  |  |  |  |
| Selected var. | TN | | | | pH, TOC, TN | | | | pH, P-PO_4_^3-^ | | | | pH, TOC, TN | | | |
|  |  | | | |  | | | |  | | | |  | | | |
|  | M9 | | | | M10 | | | | M11 | | | |  |  |  |  |
| Var. ranking | Scatter | Pearson | Spearman | RF | Scatter | Pearson | Spearman | RF | Scatter | Pearson | Spearman | RF |  |  |  |  |
| 1 | **N-NH_4_^+^** | **N-NH_4_^+^** | **N-NH_4_^+^** | **N-NH_4_^+^** | **TOC** | **TOC** | pH | **TOC** | **N-NH_4_^+^** | pH | pH | **N-NH_4_^+^** |  |  |  |  |
| 2 |  | P-PO_4_^3-^ | TN | pH | TN | N-NH_4_^+^ | **TOC** | **P-PO_4_^3-^** |  | **TOC** | **TOC** | pH |  |  |  |  |
| 3 |  |  | P-PO_4_^3-^ | DW | TP | DW | N-NH_4_^+^ | N-NH_4_^+^ |  | **N-NH_4_^+^** | **N-NH_4_^+^** | TP |  |  |  |  |
| 4 |  |  |  | TN |  | **TN** | DW | TN |  | DW | DW | TOC |  |  |  |  |
| 5 |  |  |  | P-PO_4_^3-^ |  | TP | TN | pH |  | TN | TN | TN |  |  |  |  |
| 6 |  |  |  | TOC |  |  |  | TP |  | TP | TP | Cond. |  |  |  |  |
| 7 |  |  |  |  |  |  |  | Cond. |  |  |  | DW |  |  |  |  |
|  |  |  |  |  |  |  |  |  |  |  |  | P-PO_4_^3-^ |  |  |  |  |
| Selected var. | N-NH_4_^+^ | | | | TOC | | | | N-NH_4_^+^ | | | |  |  |  |  |

***Table S17. Keystone taxa candidates’ topology parameters (betweenness centrality and vertex degree) per module. Module 9 was not included because of its low node number.***

|  | Id | Kingdom | Phylum | Class | Family | Genus | Betweenness centrality | vertex degree |
| --- | --- | --- | --- | --- | --- | --- | --- | --- |
| **M1** | ASV_896 | Bacteria | Bacteroidota | Bacteroidia | Cytophagales_unclassified | Cytophagales_unclassified | 786.444105 | 27 |
|  | ASV_2043 | Bacteria | Pseudomonadota | Alphaproteobacteria | Acetobacteraceae | Acidiphilium | 786.444105 | 27 |
|  | ASV_1162 | Bacteria | Bacteroidota | Bacteroidia | Spirosomaceae | Spirosoma | 502.564893 | 39 |
|  | ASV_370 | Eukaryota | Fungi | Chytridiomycota | Chytridiomycetes | Chytridiomycetes_unclassified | 418.361635 | 50 |
|  | ASV_705 | Bacteria | Actinobacteriota | Thermoleophilia | Solirubrobacteraceae | Solirubrobacteraceae_uncl. | 418.361635 | 50 |
|  | ASV_1634 | Bacteria | Actinobacteriota | Actinobacteria | Nocardioidaceae | Kribbella | 418.361635 | 50 |
| **M2** | ASV_123 | Bacteria | Actinobacteriota | Actinobacteria | Pseudonocardiaceae | Crossiella | 2917.799535 | 22 |
|  | ASV_92 | Bacteria | Actinobacteriota | Thermoleophilia | 67-14 | 67-14_ge | 2396.116041 | 8 |
| **M3** | ASV_221 | Bacteria | Cyanobacteriota | Cyanobacteriia | Chroococcidiopsaceae | Aliterella | 5438.213756 | 10 |
|  | ASV_183 | Bacteria | Cyanobacteriota | Cyanobacteriia | Chroococcidiopsaceae | Aliterella | 4142.854019 | 41 |
|  | ASV_293 | Bacteria | Deinococcota | Deinococci | Trueperaceae | Truepera | 592.953118 | 68 |
|  | ASV_300 | Bacteria | Actinobacteriota | Actinobacteria | Euzebyaceae | uncultured | 720.380285 | 68 |
| **M4** | ASV_865 | Bacteria | Pseudomonadota | Alphaproteobacteria | Sphingomonadaceae | Polymorphobacter | 9121.593414 | 14 |
|  | ASV_419 | Bacteria | Cyanobacteriota | Cyanobacteriia | Nostocaceae | Nostocaceae_unclassified | 351.416438 | 66 |
|  | ASV_218 | Bacteria | Cyanobacteriota | Cyanobacteriia | Nostocaceae | Nostocaceae_unclassified | 351.416438 | 66 |
| **M5** | ASV_400 | Bacteria | Cyanobacteriota | Cyanobacteriia | Nostocaceae | Nostocaceae_unclassified | 1729.069272 | 11 |
|  | ASV_776 | Bacteria | Cyanobacteriota | Cyanobacteriia | Nostocaceae | Nostocaceae_unclassified | 1088.324112 | 15 |
| **M6** | ASV_156 | Bacteria | Chloroflexota | Chloroflexia | JG30-KF-CM45 | JG30-KF-CM45_ge | 3239.797823 | 16 |
|  | ASV_5 | Bacteria | Actinobacteriota | Thermoleophilia | Solirubrobacteraceae | Solirubrobacteraceae_uncl. | 3032.724051 | 15 |
|  | ASV_306 | Bacteria | Actinobacteriota | Thermoleophilia | Solirubrobacteraceae | Solirubrobacteraceae_uncl. | 470.025382 | 54 |
| **M7** | ASV_1163 | Bacteria | Cyanobacteriota | Cyanobacteriia | Chroococcidiopsaceae | Chroococcidiopsaceae_uncl. | 2738.475742 | 27 |
|  | ASV_523 | Bacteria | Cyanobacteriota | Cyanobacteriia | Chroococcidiopsaceae | uncultured | 2738.475742 | 27 |
|  | ASV_91 | Bacteria | Bacteroidota | Bacteroidia | Spirosomaceae | Spirosoma | 1101.852178 | 22 |
|  | ASV_2795 | Bacteria | Cyanobacteriota | Cyanobacteriia | Chroococcidiopsaceae | Chroococcidiopsaceae_uncl. | 654.766362 | 28 |
|  | ASV_2142 | Bacteria | Cyanobacteriota | Cyanobacteriia | Chroococcidiopsaceae | uncultured | 558.370211 | 27 |
|  |  |  |  |  |  |  |  |  |
|  | Id | Kingdom | Phylum | Class | Family | Genus | Betweenness centrality | vertex degree |
| **M8** | ASV_20 | Bacteria | Actinobacteriota | Thermoleophilia | Solirubrobacterales_unclassified | Solirubrobacteraceae_uncl. | 6184.945817 | 16 |
|  | ASV_73 | Bacteria | Patescibacteria | uncultured | uncultured_fa | uncultured_ge | 1809.283696 | 14 |
| **M10** | ASV_1328 | Bacteria | Cyanobacteriota | Cyanobacteriia | Nostocaceae | Nostoc_PCC-73102 | 8556 | 7 |
|  | ASV_245 | Bacteria | Acidobacteriota | Blastocatellia | Blastocatellaceae | Blastocatella | 4534.083333 | 8 |
|  | ASV_1433 | Bacteria | Pseudomonadota | Gammaproteobacteria | Comamonadaceae | Rhizobacter | 4534.083333 | 8 |
|  | ASV_129 | Eukaryota | Chlorophyta | Trebouxiophyceae | Microthamniales_X | Trebouxia_jamesii | 1896.735441 | 10 |
|  | ASV_70 | Eukaryota | Chlorophyta | Trebouxiophyceae | Microthamniales_X | Trebouxia_jamesii | 1282.315171 | 13 |
|  | ASV_68 | Eukaryota | Chlorophyta | Trebouxiophyceae | Microthamniales_X | Trebouxia_jamesii | 900.838217 | 12 |
| **M11** | ASV_1011 | Bacteria | Actinobacteriota | Actinobacteria | Frankiaceae | Jatrophihabitans | 2120.63113 | 6 |
|  | ASV_115 | Eukaryota | Chlorophyta | Chlorophyta_unclassified | Chlorophyta_unclassified | Chlorophyta_unclassified | 1696.519161 | 4 |
|  | ASV_191 | Bacteria | Acidobacteriota | Blastocatellia | Blastocatellaceae | Blastocatella | 1802.592833 | 8 |
|  | ASV_193 | Bacteria | Acidobacteriota | Blastocatellia | Blastocatellaceae | Blastocatella | 1319.347179 | 7 |
|  | ASV_594 | Bacteria | Deinococcota | Deinococci | Deinococcaceae | Deinococcus | 580.033951 | 12 |
|  | ASV_87 | Bacteria | Acidobacteriota | Blastocatellia | Blastocatellaceae | Blastocatella | 580.033951 | 12 |

**I.III SUPPLEMENTARY RESULTS**

***Sampling and site description (detailed) Attention that you speak of ‘bedrock’ but maybe that it means the gravel produced by the weathering of the bedrock? Can you put more structure to this long text? Number the sites, for ex.?***

The Sør Rondane Mountains (SRM; 22 - 28° E, 71°30′ - 72°40′ S) encompass a large number of nunataks and valleys covering an area of ca. 2000 km2 (Suganuma et al. 2014) within Dronning Maud Land, East Antarctica. The Belgian station Princess Elisabeth Antarctica (“PEA”) is located in the western part of the Sør Rondane Mountains, on the granitic Utsteinen ridge (71°57′ S, 23°21′ E; 1372 m a.s.l.). For the present study, cyanobacterial communities inhabiting the most diverse geological types that could be encountered within a 70 km radius around PES were sampled (Fig. 1) in 5 nunataks, 1 ridge and 3 valleys. The Utsteinen ridge (“UT”) is characterized by intermittent east-western katabatic winds and it was sampled on an altitudinal gradient from north (1359 m a.s.l.) to south (1370 m a.s.l.). A total of 10 samples encompassing a variety of community development stages were collected. They ranged from north or sky facing bare soil consisting of small gravel without any visible communities, to well-developed BSCs with visible mosses and/or lichens located in wind protected areas, especially frequent on the northern part of the ridge. Circa 20 km to the southwest from PES and close to the main mountain range, Petrellnuten (“PT”), a single granitic nunatak (72°00′ S, 22°50′ E) was sampled at the top (472 m a.s.l.) with a northern exposure and inside its windscoop (1460 m a.s.l.) facing north-east. This included sites overgrown with black lichens to mineral soil without visible biomass. The bedrock was characterized by relatively small gravel (a few mm diameter) and a total of two samples were collected from this site. The Pingvinane range is located a few kms to the east of Petrellnuten nunatak. It consists of seven granitic nunataks, two of which were sampled for the present study. A total of 7 samples were collected from the west-southwest slope of the fourth nunatak counted from the northern top (named “PA4”; 72°00′ S, 23°00′ E; 1410 m a.s.l.), within an altitude gradient of a few meters starting from the relatively flat south-facing windscoop, mostly composed of granite gravel with no big granitic boulders and characterized by relatively small but visible communities developed below and between the most superficial gravel layer where black lichens could be occasionally encountered, to a steeper slope characterized by massive rocks and less developed communities. Here the sampled bedrock consisted predominantly of small-medium size granite gravel (mm-cm range). On the sixth nunatak of the Pingvinane range (named “PA6”; 72°01′ S, 22°59′ E; 1416 m a.s.l.), a total of 5 samples were collected along an altitude gradient with different exposures (north, south and east), ranging from sites overgrown with black mosses and lichens especially encountered in the lower part of the nunatak to mineral soils without visible communities at the top. The bedrock consisted mostly of small granite gravel (few mm). The Perlebandet range is located ca. 40 km to the northwest of PES, and it is characterized by three nunataks of gneiss rocks interlayered with marble veins in the two northernmost nunataks (designated here as “Perlebandet N'' or “PB_N” and “Perlebandet S” or “PB_S”, respectively). Twenty-two samples were taken from Perlebandet N (71°50′ S, 22°50′ E) along an altitude gradient (1256 to 1310 m a.s.l.). Five samples of the gneiss bedrock include a granulometry ranging from small gravel (few mm) to big pebbles (cm order) and encompass different degrees of community development, from mineral soils to very well-developed moss and lichen forming communities. Nine samples were characterized by ‘pure’ marble ranging from small gravel (few mm) where no visible biomass occurred to small communities living underneath the first layer of gravel and where black lichen could occur on the gravel surface. Nine other samples were characterized by marble gravel (few mm) mixed with gneiss pebbles (cm order) in which no communities were visible from the gravel surface. Perlebandet S nunatak (71°53′ S, 22°45′ E, 1354 m a.s.l.) consisted of two additional samples of marble characterized by gravel (few mm) with occasional gneiss pebbles (cm order) in which communities were not particularly visible and only occurred under a first layer of gravel, and one gneiss sample in which black lichen communities were visible on the gravel surface. Furthermore, three moraine sites were sampled, namely one in the Northeastern part of Widerøefjellet (here referred to as “Dry Valley'' or “DV”), in Yûboku-dani Valley (“YO”) and in Tvetaggen valley? Plateau?(here referred to as “Austkampane'' or “AU”). Dry Valley is located around 30 km south from PEA (72°60′ S, 23°11’ E; 1666 m a.s.l.) and is characterized by very strong katabatic winds which scrub the surfaces and inhibit the development of visible microbial communities. A total of theerty-nine samples were collected from this area characterized by little to no visible biomass on the first layer of gravel. Yûboku-dani Valley (72°41′ S, 23°48’ E; 1357 m a.s.l.) is located to the south-east of Dry Valley, and is also characterized by greenschist, gneiss and quartz mineral moraines. Four soil samples were analyzed from this area which is up to now the only investigated site where lakes were discovered in this region. They are generally year-round frozen and contain conspicuous benthic cyanobacterial mats. Dried mats are also present on the shores. For this study, only mineral soil samples from the shore and from sites situated deeper inside the valley were collected. Last, Austkampane (71°40′ S, 25°90’ E; 930 m a.s.l.) is an extremely windy site (is it a valley or what?) located 70 km east from PES, characterized by amphibolite on which almost no life was visible. Three collected samples were characterized by small gravel (few mm). Pictures of the studied localities are shown in Fig. 1 and details of the collected samples are given in Table S1.

***Environmental variables***

Elevation data and the main physicochemical parameters of the investigated soils are summarized in Fig. S3 and Table S1. Samples were grouped by their bedrock substrate type (granite, gneiss, marble and moraine). Moraine soils were taken from sites with different altitudes: Dry Valley was the highest site (~ 1600 m), Yûboku-dani Valley was lower (~ 1300 m) and Austkampane (~ 900 m) was the lowest site among all sampled sites. pH values were highest in marble soils (median 9.17), whilst all the other bedrock substrate types were on average more acidic (median 6.36). The exceptional alkalinity of marble is due to the characteristic salt accumulation in such bedrock. Different amounts of total organic carbon (TOC) were measured, ranging from 0.19 % (in moraine soils) to 10.95 % (in gneiss soils), and TOC was the highest in soils with developed biocrusts (lichens or moss dominated). A reverse trend was observed for dry weight, with lowest values recorded in gneiss soils (77.04 - 99.05 %) and highest in moraine (95.64 - 99.8 %) and marble (94.48 - 99.93 %), whilst granite (88.91 - 99.87 %) had intermediate values. Total N values were particularly low in moraine soils, with an average of 0.01 % compared to the other soils which had average values comprised between 0.08 and 0.15 %. Highest values of N-NH_~~4~~_^+^ were recorded in gneiss (46 mg kg^-1^) and granite (30 mg kg^-1^) whereas the lowest values were observed in moraine (0.5 mg kg^-1^) and marble (0.7 mg kg^-1^). Conductivity did not show strong differences across the sampled sites, though very high values were found in two samples of the Dry Valley and in one of Austkampane (> 1000 μS cm^-1^). P-PO_4_^3-^ values were significantly different in all bedrock substrate types, being highest in granitic (65.3 ± 31; min: 26.4; max: 151.1 mg kg^-1^) and lowest in marble soils (8.6 ± 3.3; min: 6.5; max: 18.5 mg kg^-1^), although a very high value (337 mg kg^-1^) was found in a moraine sample from Austkampane that was characterized by an algal community. This latter sample also showed the highest N-NO_3_^-^ concentration (24 mg kg^-1^), that was, on average, below 1 mg kg^-1^ in all other samples. Total P was significantly higher in gneiss bedrock compared to the others.
